# circRNAs expressed in human peripheral blood are associated with human aging phenotypes, cellular senescence and mouse lifespan

**DOI:** 10.1007/s11357-019-00120-z

**Published:** 2019-12-06

**Authors:** Shahnaz Haque, Ryan M. Ames, Karen Moore, Luke C. Pilling, Luanne L. Peters, Stefania Bandinelli, Luigi Ferrucci, Lorna W. Harries

**Affiliations:** 1 RNA-Mediated Mechanisms of Disease Group, Institute of Biomedical and Clinical Sciences, University of Exeter Medical School, University of Exeter, RILD South, Barrack Road, Exeter, EX2 5DW UK; 2 Biosciences, University of Exeter, Exeter, UK; 3 College of Life and Environmental Sciences, University of Exeter, Exeter, UK; 4 Epidemiology and Public Health, University of Exeter Medical School, University of Exeter, Exeter, UK; 5 The Jackson Laboratory Nathan Shock Centre of Excellence in the Basic Biology of Aging, Bar Harbor, ME USA; 6 Geriatric Unit, USL Toscana Centro, Florence, Italy; 7 National Institute on Aging, Clinical Research Branch, Harbor Hospital, Baltimore, MD 21225 USA

**Keywords:** Circular RNA, Aging phenotypes, Senescence, Median strain lifespan

## Abstract

Circular RNAs (circRNAs) are an emerging class of non-coding RNA molecules that are thought to regulate gene expression and human disease. Despite the observation that circRNAs are known to accumulate in older organisms and have been reported in cellular senescence, their role in aging remains relatively unexplored. Here, we have assessed circRNA expression in aging human blood and followed up age-associated circRNA in relation to human aging phenotypes, mammalian longevity as measured by mouse median strain lifespan and cellular senescence in four different primary human cell types. We found that circRNAs *circDEF6*, c*ircEP300*, *circFOXO3* and *circFNDC3B* demonstrate associations with parental longevity or hand grip strength in 306 subjects from the InCHIANTI study of aging, and furthermore, *circFOXO3* and *circEP300* also demonstrate differential expression in one or more human senescent cell types. Finally, four circRNAs tested showed evidence of conservation in mouse. Expression levels of one of these, *circPlekhm1*, was nominally associated with lifespan. These data suggest that circRNA may represent a novel class of regulatory RNA involved in the determination of aging phenotypes, which may show future promise as both biomarkers and future therapeutic targets for age-related disease.

**Electronic supplementary material:**

The online version of this article (10.1007/s11357-019-00120-z) contains supplementary material, which is available to authorized users.

## Introduction

Aging is a multifactorial process leading to gradual deterioration of physical and physiological functionality at the cellular, tissue and organ levels. It is the primary risk factor for chronic aging pathologies such as cancer, sarcopenia, diabetes, cardiovascular disorders and neurodegenerative illnesses that account for the bulk of morbidity and mortality in both the developed as well as developing world (Kirkland 2016). Physiological parameters such as loss of muscle mass, frailty, immobility and cognitive impairment increase the risk of developing geriatric syndromes (Fabbri et al. 2016; Narici and Maffulli 2010). The molecular processes that decline with advancing age underpin the phenotypes of aging. At the cellular level, hallmarks of aging include genomic instability, telomere attrition, epigenetic alterations, loss of proteostasis, deregulated nutrient sensing, mitochondrial dysfunction, cellular senescence, stem cell exhaustion and altered intercellular communication (Lopez-Otin et al. 2013).

Changes in gene expression have been reported in many age-related diseases (Yang et al. 2015). In addition to an increase in transcriptional noise and aberrant production and maturation of mRNA transcripts (Bahar et al. 2006; Harries et al. 2011), studies report associations between gene expression and the development of age-associated syndromes of the muscle (Noren Hooten et al. 2010; Welle et al. 2004) as well as neurodegenerative conditions such as Alzheimer’s disease and Parkinson’s disease (Miller et al. 2017; Shamir et al. 2017). Differential expression of genes involved in inflammatory, mitochondrial and lysosomal degradation in aging tissues has also been reported (de Magalhaes et al. 2009). Gene expression is regulated at many levels. Changes in the regulation and pattern of alternative splicing are associated with age in several human populations and are also evident in senescent cells of different lineages, where they may drive cellular senescence, since restoration of levels reverses multiple senescence phenotypes (Latorre et al. 2017; Latorre et al. 2018a; Latorre et al. 2018b; Latorre et al. 2018c; Lye et al. 2019). Notably, non-coding RNAs also demonstrate associations with aging or senescence and may be of equal importance (Abdelmohsen et al. 2012; Boulias and Horvitz 2012; Gorospe and Abdelmohsen 2011)**.**

Circular RNAs (circRNAs) are a recently discovered class of non-coding RNA molecules that are thought to have important roles in regulation of gene expression and human disease (Haque and Harries 2017). circRNAs are formed by the back splicing of downstream exons to the 3′ acceptor splice site of upstream exons and result in a covalently closed circular structure containing one or more exons. They have been proposed to be key regulators of gene expression by various mechanisms including sequestration of RNA-binding proteins and miRNAs or by acting as a competitor of linear splicing of their cognate genes (Memczak et al. 2013). The possibility that a single circRNA could sequester several such RNA regulators suggests that this class of non-coding RNAs could modulate many cellular and physiological processes through multiple pathways. circRNAs are known to accumulate in older organisms (Gruner et al. 2016), and some have been reported to be implicated in cellular senescence (Du et al. 2017; Du et al. 2016). Despite these promising findings, their role in aging remains relatively unexplored.

We hypothesized that expression of some circRNAs may be associated with advancing age, aging phenotypes, lifespan or cellular senescence. Changes in circRNA expression over a 5-year period were assessed in relation to age, combined parental longevity score (PLS) and hand grip strength. We then assessed expression levels of 15 circRNAs in early passage and late passage primary human dermal fibroblasts, cardiomyocytes, astrocytes and vascular endothelial cells. Finally, the junction sequences of relevant exons were examined for conservation between mouse and humans and where evidence was present that the back-spliced junction, and thus, the circular RNA were conserved; we assessed expression in relation to longevity in six strains of mice with differential median strain longevities.

We present here evidence that although effects on age itself did not replicate in the wider sample set, the expression levels of *circEP300* (*β* = − 0.065, *P* = 0.001) and *circFOXO3* (*β* = − 0.060, *P* = 0.002) were negatively associated with parental longevity score. *circDEF6* was positively associated with parental longevity score (*β* = 0.070, *P* = 0.024) although this did not reach multiple testing thresholds. *circFNDC3B* was also nominally associated with hand grip strength (*β* = 0.004, *P* = 0.039). circRNAs (7/12 (58%)) expressed in senescent human primary astrocytes, endothelial cells, fibroblasts or cardiomyocytes also demonstrated dysregulated expression in one or more cell types. Comparative sequence analysis suggested that four circRNAs may be conserved in mice. When assessed, c*ircPlekhm1* transcript level in spleen was also demonstrated to be positively associated with mouse median strain lifespan (*β* = 0.0025; *P* = 0.017). These results suggest that some age-related circRNAs may play roles in molecular drivers of aging such as cellular senescence, and hence may represent potential contributors to lifespan or other human aging phenotypes.

## Methods

### InCHIANTI cohort and selection of participants

The InCHIANTI study of Aging is a population study of aging (Ferrucci et al. 2000). Participants undertook detailed assessment of health and lifestyle parameters at baseline, and again at three subsequent follow-ups (FU2 2004–2006, FU3 2007–2010 and FU4 2012–2014). The present study used participants from the third and fourth follow-up visits (FU3 and FU4). RNA samples and clinical/phenotypic data were already available for 698 participants at FU3. The collection of the FU4 samples and data comprise part of this study. During the FU4 interviews in 2012/2013, blood and clinical/phenotypic data were collected from 455 study participants. These data were cross-checked against RNA samples and clinical/phenotypic data already held from FU3, to ensure that sample and phenotypic data was available from both collections. Sample-associated data included measures of potential confounding factors such as BMI, sex, level of education (none, elementary, secondary, high school and university), study site, smoking and white blood counts (neutrophil, lymphocyte, monocyte, eosinophil percentages). Characteristics of the study population are given in Table 1. Informed consent was obtained from all participants. Ethical approval was obtained from the Instituto Nazionale Riposo e Cura Anziani institutional review board, Italy.

**Table 1:** Participant demographics, population demographics and clinical characteristics of InCHIANTI study participants assessed in this work, (A) demographics and (B) clinical characteristics

A				Number	Percentage
Participants				306	100
Age (years)					
30–39				24	7.84
40–49				37	12.09
50–59				31	10.13
60–69				32	10.46
70–79				116	37.91
80–89				63	20.59
90–100				3	0.98
Gender					
Male				143	46.73
Female				163	53.27
Pack years smoked (lifetime)					
None				164	53.59
< 20				79	25.82
20–39				43	14.05
40+				20	6.54
Study site					
Greve				146	47.71
Bagno				160	52.29
Education level attained					
Nothing				22	7.19
Elementary				124	40.52
Secondary				56	18.30
High school				50	16.34
Professional school				34	11.11
University or equivalent				20	6.54
B	*n*	Mean	SD	Min	Max
Age (years)	306	66.96	16.06	30.00	94.00
BMI	305	27.15	4.35	15.01	42.99
White blood cell count (*n*, K/μLs)	305	6.40	1.59	2.10	13.00
Neutrophils (%)	305	56.59	8.35	34.20	81.20
Lymphocytes (%)	304	31.69	7.67	9.80	51.20
Monocytes (%)	304	8.04	2.20	3.90	21.30
Eosinophils (%)	304	3.18	2.17	0.00	21.50
Parental longevity score	206	− 0.02	0.81	− 2.46	1.71
Mean hand-grip strength (kg)					
Follow-up 3	305	29.65	12.49	2.50	70.75
Follow-up 4	291	28.66	12.30	5.00	65.50

### Generation of circRNA profiles from old and young human peripheral blood

Circular RNA profiles were initially generated in parallel from two sets of pooled peripheral blood total RNA samples using a modified ‘CircleSeq’ procedure (Lopez-Jimenez et al. 2018). 2 μg RNA (RNA integrity number (RIN) = 6.4) was assessed in two separate pools from 20 ‘young’ samples (median age = 33 years, range 30–36 years, 55% female, 45% male; RIN 5.6) and 20 ‘old’ samples (median age 87 years, range 86–95 years, 90% female 10% male, RIN 7.7). Each pooled sample was divided into two aliquots, one of which was treated with 20 units RNAse R (Epicentre, Madison, USA) at 30 °C for 30 min to remove linear RNA, the other sample being mock-treated using 1 μL RNase-free water in place of the enzyme. Both aliquots were cleaned and concentrated using 2 volumes of RNA clean beads (Beckman Coulter, Indianapolis, USA) to remove the enzyme. The results of the RNase R treatment were confirmed on a high-sensitivity RNA screentape (Agilent, Santa Clara, USA). Ribosomal RNA was removed, and indexed sequencing libraries made using the libraries were determined by qPCR and adjusted for size using Tapestation D1000 analysis (Agilent, Santa Clara, USA). Ribosomal RNA was removed, and indexed sequencing libraries made using the Illumina RNASeq protocol. The library concentrations were determined by qPCR and adjusted for size using the data from the Tapestation D1000 analysis. Libraries were pooled in equimolar quantities, denatured and diluted to 12.0 pM + 1% PhiX for clustering and then underwent 125 paired-end Illumina sequencing in four lanes using TruSeq SBS reagents (V3).

### Analysis of circRNA profiles

RNase R and mock-treated sequence data were assembled, and putative circular RNAs were identified using PTESFinder (Izuogu et al. 2016) with the human genome (hg19) reference files provided with the software, a segment size of 65 and a uniqueness score of 7. The remaining parameters were left to default settings. To calculate a comparable measure of circular RNA abundance between samples, we used a measure termed back-spliced reads per million mapped reads (bpm) for each circular RNA defined as

$$ {\mathrm{bpm}}_i=\left(\frac{j_i}{\sum_{a=1}^n{j}_a+{\sum}_{b=1}^n{c}_b\ }\right)\times {10}^6 $$

where *J*_*i*_*i*s the number of reads mapped to the back-spliced junction of the circular RNA, *c* is the number of reads mapped to canonical sites of the gene with the circular RNA and *n* is the number of circular RNAs identified. This measure is designed to be similar to the commonly used reads per kilobase per million mapped reads (RPKM) metric used regularly to estimate gene expression from RNA-Seq data.

In addition to circular RNA detection using PTESFinder, reads from all samples were also mapped to the human genome reference (hg19) obtained from iGenomes using Tophat v2.1.0 with the pre-set sensitive alignment parameters in paired-end mode (Trapnell et al. 2009). The number of reads mapping to each exon of each gene was then calculated using FeatureCounts v2.0.0 with parameters for unstranded alignment, paired reads, count multimapping reads and assigning reads to overlapping features (Liao et al. 2013; Liao et al. 2014). Counts were used to calculate RPKM per exon using the standard method to compare the expression of each exon across samples.

### Pathway analysis of differentially regulated circRNA host genes

circRNAs showing expression differences between the pooled old and the pooled young samples were ranked by RPKM and fold change. To assess whether circRNAs demonstrating expression differences between young and old pools were enriched in genes derived from specific molecular or biochemical function groups, we carried out a Cytoscape version 2.5.2 plug-in ClueGO analysis. This platform queries over-representation of query genes in specific KEGG, REACTOME and WikiPathways (Bindea et al. 2009). The linear genes hosting the top 10% most abundantly expressed circRNAs in young and old pools for the circRNA profile were queried against KEGG_20.11.2017, REACTOME_Pathways_20.11.2017 and WikiPathways_20.11.2017. Outputs were selected based on ‘enrichment/depletion’ through a two-sided hypergeometric test with Bonferroni step down for *P* value correction with the selected ontology reference set of chosen genes. The GO terms were used to group functional pathways, and the leading functional grouping was based on highest significant kappa score.

### Design of qPCR assays for circRNA validation

Levels of individual circRNA in young and old pools were ranked by abundance. circRNAs demonstrating evidence of altered expression with age fell into three classes: those expressed exclusively in old, those expressed exclusively in young, and those expressed in both young and old, but with evidence that levels were different between the pools. We selected five circRNAs exclusively expressed in young (*circITGAX*, *circPLEKHM1*, *circDEF6*, *circATP6V0A1* and *circASAP1*), five exclusively expressed in the old (*circFOXO3*, *circFNDC3B*, *circAFF1*, *circCDYL* and *circXPO7)*, as well as five expressed in both pools but demonstrating evidence of altered expression (*circMIB1*, *circMETTL3*, c*ircBCL11B*, *circZC3H18* and *circEP300*), where sequence and assay design constraints allowed for to design specific assays to unique back-spliced junction for qRTPCR follow-up.

### circRNA probe design

Custom-designed qRTPCR assays for quantification of relative expression were designed to unique back-spliced circRNA junctions (Thermo Fisher, Foster City, USA), the sequences of which are given in Online Resource 1. Each target sequence was checked for the presence of single nucleotide polymorphisms in potential primer or probe binding regions prior to ordering. Assays were ordered as custom single-tube assays from Thermo Fisher (Foster City, USA). Each circRNA probe was validated using standard curve analysis using 1:10 serial dilutions of synthetic oligonucleotides homologous to the back-spliced junctions.

### Assessment of associations between circRNA expression and aging phenotypes in the InCHIANTI cohort

RNA samples and phenotypic data were available from 306 individuals at both follow-up 3 (FU3) and follow-up 4 (FU4) of the InCHIANTI study of aging. Characteristics of participants are given in Table 1. We assessed the expression of 15 age-associated circRNAs demonstrating the most marked differential expression with age between young and old pools as described above. Aging parameters assessed were age itself, parental longevity score (PLS) and hand grip strength. Participants aged 65 + years were categorised for PLS based on the age at death of their parents. Short, intermediate and long-lived cut-offs were calculated separately for mothers and fathers based on the normal distribution of age at death in the cohort, as described in Dutta et al. (2013a). Mothers and fathers aged < 49 years or < 52 years at death respectively were classed as premature and excluded. To standardize parental age of death, a *Z* score was generated for combined maternal and paternal measures of parental longevity. Hand-grip strength was measured in kilograms using a dynamometer, with repeated measurements at both FU3 and FU4.

### Reverse transcription and pre-amplification of circRNAs in human peripheral blood RNA

cDNA synthesis was carried out using 100 ng total RNA using the High-Capacity cDNA Reverse Transcription Kit (Thermo Fisher, Foster City, USA) according to manufacturer’s instructions (Fisher Scientific, New Hampshire, USA) in a final reaction volume of 10.0 μL per sample. Reactions (samples in 96-well plates) were run at 25 °C for 10 min, 37 °C for 120 min, 85 °C for 5 min followed by an inactivation period for 95 °C for 10 min. Pre-amplification of circRNA expression was carried out using 5 μL TaqMan PreAmp master mix (Thermo Fisher, Foster City, USA), 2.5 μL pooled assay mix and 2.5 μL cDNA in a final reaction volume of 10 μL per sample. Cycling conditions were one cycle of 95 °C for 10 min followed by 14 cycles of 95 °C for 15 s with 60 °C for 4 min followed by 95 °C for 10 min. Pre-amplified samples were then diluted 1:10 and maintained on ice prior to analysis.

### Assessment of associations between circRNA expression in peripheral blood RNA and human aging phenotypes

The expression profiles of selected circRNAs were then measured in total peripheral blood mRNA using custom-designed OpenArray plates on the Thermo Fisher 12K Flex platform (Thermo Fisher, Foster City, USA). Reaction mixes contained 2.5 μL 2✕ OpenArray Real-Time Master Mix, diluted pre-amplified cDNA (1.2 μL) and RNase-free dH_2_O (1.3 μL) (Thermo Fisher, Foster City, USA). circRNA expression was measured relative to the geometric mean of the entire set of transcripts, with the expression of each individual circRNA normalised to the global mean of expression of that circRNA across the samples. Samples were run in three technical triplicates. Association of circRNAs with age in InCHIANTI was carried out by multivariate linear regression, adjusted for potential confounders BMI, sex, level of education (none, elementary, secondary, high school and university), study site, smoking and white blood counts (neutrophil, lymphocyte, monocyte, eosinophil percentages) while age was additionally adjusted for all other measures of association in the aging human cohort. We assessed association of circRNA with hand grip strength and parental longevity score (PLS) (Dutta et al. 2013b; Dutta et al. 2013c) as a proxy measure of longevity in humans. Statistical analysis was completed using StataSE15 (StataCorp, TX, USA). Figures were generated using GraphPad Prism 8.1.2 (GraphPad Software, San Diego, USA).

### Assessment of circRNA expression in human primary senescent cells of different lineages

The expression levels of the 15 candidate circRNAs analysed above were also assessed in relation to cellular senescence, in senescent and early passage primary human primary fibroblasts, endothelial cells, astrocytes and cardiomyocytes using high-throughput qRTPCR on the 12K Flex OpenArray platform (Thermo Fisher, Foster City, USA)*.* Samples were run in three biological replicates and three technical replicates. Senescent cells had been generated and characterised in previous work by our group, and culture conditions and details of assessment of senescence are reported elsewhere (Latorre et al. 2017; Latorre et al. 2018a; Latorre et al. 2018b; Latorre et al. 2018c; Lye et al. 2019). RNA samples from this work were available for use. circRNA levels were assessed in three biological and three technical replicates from early and late passage human primary cells of four different cell types. Early passage young cells were at populati*o*n doubling (PD) of 24 for astrocytes, 28 for cardiomyocytes, 24 for endothelial cells and 25 for fibroblasts, whilst late passage senescent cells were at PD = 84 for astrocytes, 75 for cardiomyocytes, 65 for endothelial cells and 63 for fibroblasts. Senescent cell load in these samples was ~ 75% for fibroblasts, ~ 55% for endothelial cells, ~ 38% for cardiomyocytes and ~ 36% for cardiomyocytes (Latorre et al. 2017; Latorre et al. 2018a; Latorre et al. 2018b; Latorre et al. 2018c; Lye et al. 2019). In all cases, growth of the culture had slowed to less than 0.5 PD/week. Differential circRNA expression in senescent cells was then assessed by one-way ANOVA using StataSE15 (StataCorp, TX, USA). Figures were generated using GraphPad Prism 8.1.2 (GraphPad Software, San Diego, USA).

### Assessment of circRNA conservation between mouse and human

We assessed whether the 15 circRNAs identified in our human study were likely to be conserved in mouse by aligning the mouse and human exon junction sequences using the Blat tool in the UCSC genome browser (https://genome.ucsc.edu). Quantitative real-time PCR assays were developed to unique back-spliced junctions of conserved circRNAs. Probe and primer sequences are given in Online Resource 2. circRNA expression was then measured in mouse spleen and muscle tissue and assessed in relation to lifespan by analysis of levels in six strains of male mice (A/J, NOD.B10Sn-H2^b^/J, PWD/PhJ, 129S1/SvlmJ, C57BL/6J and WSB/EiJ) selected on the basis of divergent median strain longevity (Yuan et al. 2009). Animal husbandry, handling, animal characteristics and sample preparation protocols have been previously described (Lee et al. 2016). Tissue samples were obtained from cross-sectional study conducted in the same compartment and in the same period of time as described in Yuan et al. (2009). Spleen and quadricep muscle tissues were excised immediately after sacrifice and shipped from the Jackson Laboratory using RNAlater-ICE Collection protocol (Life Technologies, Carlsbad, CA). In this method, tissues are submerged in RNAlater stabilization solution; an aqueous tissue storage reagent used to rapidly permeate tissues and stabilize RNA from fresh specimens and stored at – 20 °C or below for later use.

### RNA extraction and reverse transcription from mouse tissues

Total RNA was extracted using the TRI Reagent/chloroform phase separation according to manufacturer’s instructions. Briefly, tissues stored in RNA later were drained, and then placed in 1 mL TRI Reagent solution containing 10 mm MgCl_2_. Samples were homogenized for 15 min (spleen) or 30 min (muscle) using bead mills (Retsch Technology GmbH, Haan, Germany). This was followed by a phase separation using chloroform. Total RNAs in the separated RNAs were precipitated from the aqueous phase through overnight incubation with isopropanol at − 20 °C. The following morning, RNA pellets were washed twice with ethanol and resuspended in RNase-free dH_2_O. Complementary DNA (cDNA) was generated from 100 ng RNA using the Evocript Universal cDNA Master Synthesis kit according to the manufacturer’s instructions (Roche, Switzerland).

### Assessment of circRNA expression in mouse spleen and muscle

circRNAs selected on the basis of interspecies sequence conservation were validated in mouse spleen and muscle tissue. Expression levels of conserved circRNAs were assessed in relation to median strain lifespan by relative quantification. Quantitative qRTPCR was carried out for circRNAs (*circFoxo3*, *circMib1*, *circPlekhm1* and *circXpo7)* in relation to the *Pol2ra*, *Trfc* and *Ipo8* endogenous control genes, selected on the basis of lack of age association in a previous study (Harries et al. 2011). Reaction mixes contained cDNA (0.5 μL), TaqMan Universal PCR mastermix II (2.5 μL, no AmpErase UNG (Thermo Fisher, Foster City, USA), dH_2_O (1.75 μL, Fisher Scientific, USA), and TaqMan gene assay (0.25 μL, Thermo Fisher, Foster City, USA) in a 5 μL final reaction volume. The reaction mixes were centrifuged at 3000 rpm, vortexed and centrifuged again at 3000 rpm and transferred to 384-well qRTPCR plates. qRTPCR was run at 50 °C for 2 min, 95 °C for 10 min and 50 cycles of 15 s at 95 °C for 30 s and 1 min at 60 °C. Each sample assay was conducted in three technical triplicates. Expression levels of circRNAs in young and old mouse tissues were measured relative to the geometric mean of the entire set of transcripts, with the expression of each individual circRNA normalised to the global mean of expression of each circRNA across the samples. Linear regression analysis was carried out to assess the association of expression of circRNA using StataSE15 (StataCorp, TX, USA).

## Results

### circRNA profile in peripheral blood of aging humans

One hundred sixty-six to 167M reads were obtained from the RNAse R-treated pools and 157–163M reads from the mock-treated pools with a mean *Q* score of 34.6–35.1 and total error rate of 0.47–0.53%. A total of 2207 circRNAs were expressed in human peripheral blood. Of these, 184 circRNAs were found in both the young and old samples, 431 were exclusively expressed in the young sample pool and 1592 were exclusively expressed in the old sample pool (Online Resource 3). We selected 15 circRNAs for further analysis: 5 expressed exclusively in the young pool, 5 expressed exclusively in the old pool and 5 expressed in both pools but showing the most discrepant expression for further study. These were *circITGAX*, *circPLEKHM1*, *circDEF6*, *circATP6V0A1* and *circASAP1* which showed exclusive expression in the young; *circFOXO3*, *circFNDC3B*, *circAFF1*, *circCDYL* and *circXPO7* which showed exclusive expression in the old; and *circMIB1*, *circMETTL3*, *circEP300*, *circZC3H18* and *circBCL11B* that were expressed, but differentially so in both sample pools.

### Pathway analysis of circRNA expressed in aging humans

Pathway enrichment for the genes hosting the top 10% most abundant circRNAs in each of young and old pooled peripheral blood samples was performed using ClueGO cytoscape (Bindea et al. 2009). In the young peripheral blood, the top 10% most abundant circRNAs derived from genes associated with negative regulation of ATP metabolic processes and in transmission of synaptic signals. The leading edge genes hosting circRNAs for negative regulation of ATP processes were *SNCA*, *STAT3* and *UFSP2*, whilst those associated with synaptic vesicle endocytosis were *FCH02*, *PICALM*, *PIP5K1C* and *SNCA*. Genes hosting circRNAs were primarily localised in pathways involved in phagocytosis, circadian regulation, cancer pathways and golgi-associated vesicle budding in the blood from aged donors (Table 2).

**Table 2:** Pathways enriched in age-associated circRNAs

Pathway	*p* value	Number Of Genes	Genes
Expressed only in old			
Fc gamma R-mediated phagocytosis	0.005	4	*ARPC1B, ASAP1, PIP5K1C, VASP*
Exercise-induced Circadian Regulation	0.006	3	*CRY2, NCOA4, TAB2*
Pathways Affected in Adenoid Cystic Carcinoma	0.018	4	*ERBB2, FOXO3, KANSL1, MGA*
Endometrial cancer	0.041	3	*AXIN1, ERBB2, FOXO3*
trans-Golgi Network Vesicle Budding	0.035	3	*DNAJC6, IGF2R, PICALM*
Clathrin derived vesicle budding	0.049	3	*DNAJC6, IGF2R, PICALM*
Golgi Associated Vesicle Biogenesis	0.069	3	*DNAJC6, IGF2R, PICALM*
Cargo recognition for clathrin-mediated endocytosis	0.062	5	*FCHO2, IGF2R, PICALM, REPS1, UBQLN1*
Clathrin-mediated endocytosis	0.049	8	*DNAJC6, FCHO2, GAPVD1, IGF2R, PICALM, PIP5K1C, REPS1, UBQLN1*
Expressed only in young			
Negative regulation of ATP metabolic process	0.004	3	*SNCA, STAT3, UFSP2*
Synaptic vesicle recycling	0.009	4	*FCHO2, PICALM, PIP5K1C, SNCA*
Presynaptic endocytosis	0.018	4	*FCHO2, PICALM, PIP5K1C, SNCA*
Synaptic vesicle endocytosis	0.017	4	*FCHO2, PICALM, PIP5K1C, SNCA*
Expressed in both old and young, but demonstrating differential expression			
Huntington’s disease_Homo sapiens_hsa05016	0.014	2	*ATP5C1, EP300*
Pyruvate metabolism_Homo sapiens_hsa00620	0.037	1	*HAGH*
Notch signaling pathway_Homo sapiens_hsa04330	0.045	1	*EP300*

The ClueGo pathway results for pathways potentially targeted by genes generating the top 10% of circRNAs differentially expressed with age are presented here aligned to the hg19 genome alignment. Number of genes = number of differentially expressed genes in each pathway

### *circPLEKHM1*, *circMETTL* and *circFNDC3B* expression levels are associated with aging phenotypes in humans

**Fig. 1:**
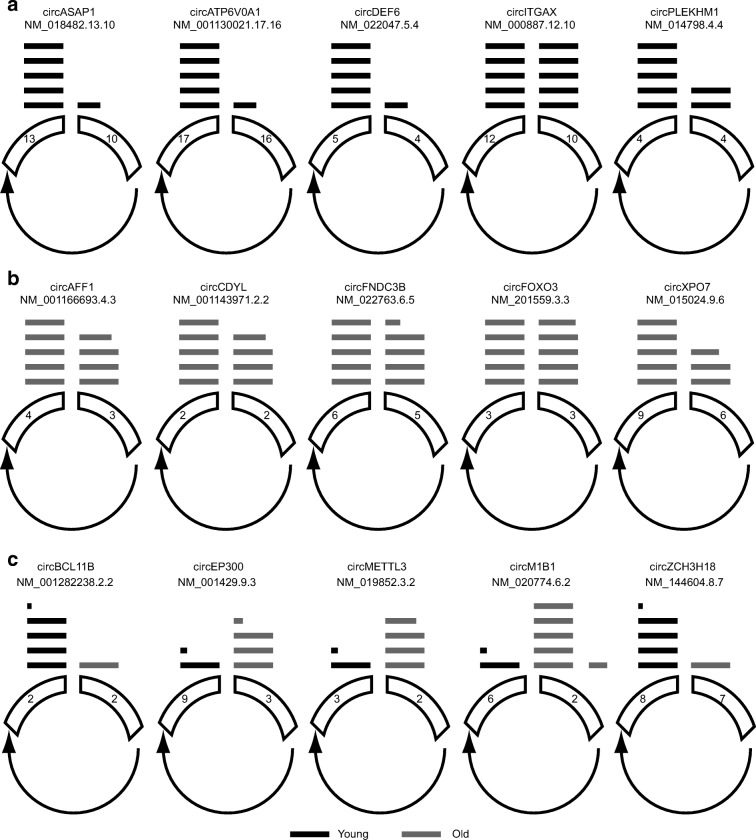
Circular RNA junction schematics for the top 5 most abundant circular RNAs uniquely found in young (**a**) and old samples (**b**). Also shown are junction schematics for the top 2 and 3 most abundant common circular RNAs found in young and old samples respectively (**c**). Each schematic shows the identified back-spliced exon or exons. The relative read depth at each back-spliced junction is shown by the number of bars above each junction and is scaled by linear interpolation, where the back-spliced junctions with 1 and 10 bars represent the junctions with the lowest and highest read depth respectively. Black and grey bars show relative read depth at junctions in young and old samples respectively

**Fig. 2:**
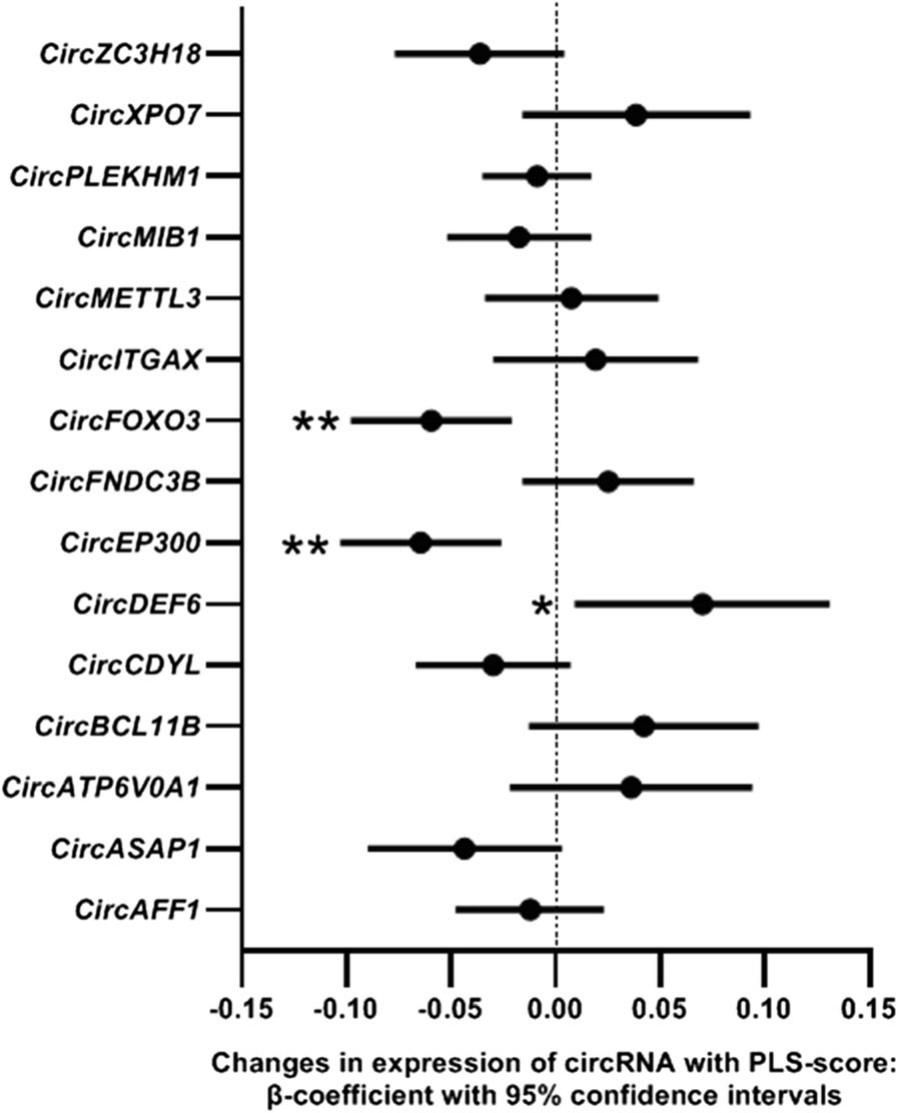
circRNA expression is associated with combined parental longevity. Forest plot illustrating the association between peripheral blood circRNA expression and combined human parental longevity score (PLS) in participants from the InCHIANTI study of aging. *N* = 306 individuals. The beta-coefficient of the association is given on the *X*-axis, and the identity of the gene is given on the *Y*-axis. Lines attached to each data point represent 95% confidence intervals (95% CI). Statistical significance is indicated by stars, *< 0.05, **< 0.005

**Fig. 3:**
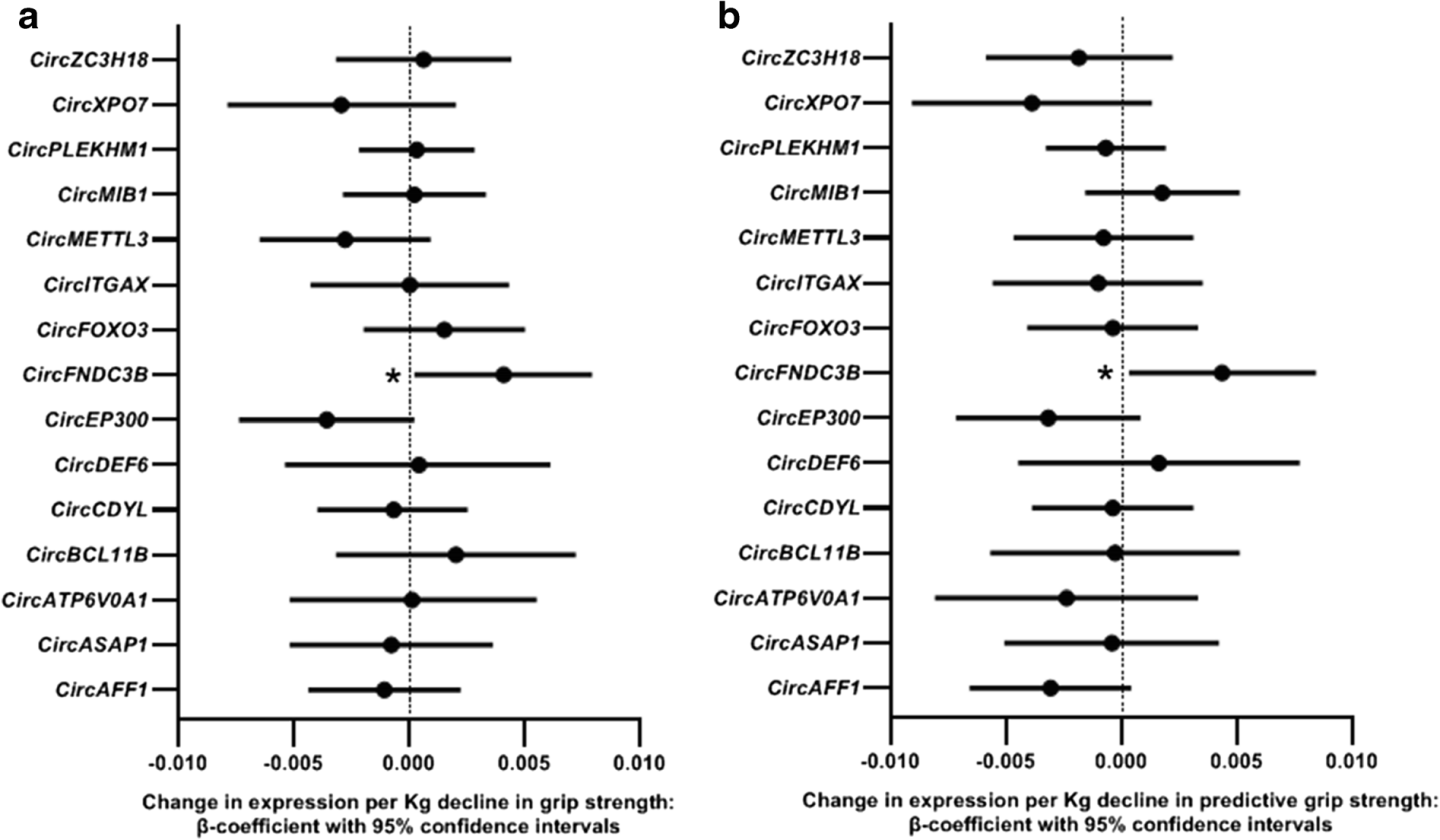
Peripheral blood *circFNDC3B* expression is nominally associated with hand grip strength Forest plot illustrating the association between circRNA expression and hand grip strength in participants from the InCHIANTI study of aging. Associations with grip strength are shown both **a** cross-sectionally from follow-up 3 (FU3) and **b** longitudinally, from follow-up 4 (FU4). *N* = 306 individuals. The beta-coefficient of the association is given on the *X*-axis, and the identity of the gene is given on the *Y*-axis. Lines attached to each data point represent 95% confidence intervals (95% CI). Statistical significance is indicated by stars, *< 0.05, **< 0.005

The structures of the 15 circRNAs selected for follow-up were predicted based on the sequencing read depth for each exon and are presented in Fig. 1. Exon structures presented as read depth plots are given in Online Resource 4. Although we demonstrated no associations with age itself, we did identify associations between some circRNAs and human aging phenotypes. *circEP300* and *circFOXO3* both demonstrated negative associations with combined parental longevity score (*β* = − 0.065 and − 0.060; *P* = 0.001 and 0.002 respectively), after adjustment for multiple testing. *circDEF6* was positively correlated with parental longevity scores but demonstrated nominal significance only (*β* = 0.070, *P* = 0.024) (Table 3, Fig. 2). A positive association was also identified both cross-sectionally (*β* = 0.004, *P* = 0.039) and longitudinally (*β* = 0.004, *P* = 0.038) between *circFNDC3B* expression and hand grip strength (Table 4, Fig. 3), although these were nominal only.

**Table 3:** circRNA expression in relation to combined parental longevity score

circRNA	β-Coefficient	*p* value	95% CI
*circAFF1*	− 0.012	0.485	− 0.048–0.023
*circASAP1*	− 0.044	0.064	− 0.090–0.003
*circATP6V0A1*	0.036	0.223	− 0.022–0.094
*circBCL11B*	0.042	0.136	− 0.013–0.097
*circCDYL*	− 0.030	0.109	− 0.067–0.007
*circDEF6*	*0.070*	*0.024*	*0.009*–*0.131*
*circEP300*	**− 0.065**	**0.001**	**− 0.103–− 0.026**
*circFNDC3B*	0.025	0.239	− 0.016–0.066
*circFOXO3*	**− 0.060**	**0.002**	**− 0.098–− 0.021**
*circITGAX*	0.019	0.440	− 0.030–0.068
*circMETTL3*	0.007	0.730	− 0.034–0.049
*circMIB1*	− 0.018	0.310	− 0.052–0.017
*circPLEKHM1*	− 0.009	0.493	− 0.035–0.017
*circXPO7*	0.038	0.162	− 0.016–0.093
*circZC3H18*	− 0.036	0.078	− 0.077–0.004

Beta coefficients, *p* values and 95% confidence intervals (95% CI) are given for associations between circRNAs expression and combined parental longevity (PLS) score. Two hundred ninety-one samples were assessed. Genes demonstrating statistically significant results below the multiple testing limit of 0.003 are indicated in italics, whilst those demonstrating nominal associations only are given in bold type

**Table 4:** circRNA expression in relation to grip strength

circRNA	Grip strength	β-Coefficient	*p* value	95% CI
*circAFF1*	Cross-sectional	− 0.001	0.508	− 0.004–0.002
	Longitudinal	− 0.003	0.081	− 0.007–0.000
*circASAP1*	Cross-sectional	− 0.001	0.713	− 0.005–0.004
	Longitudinal	0.000	0.854	− 0.005–0.004
*circATP6V0A1*	Cross-sectional	0.000	0.965	− 0.005–0.005
	Longitudinal	− 0.002	0.403	− 0.008–0.003
*circBCL11B*	Cross-sectional	0.002	0.443	− 0.003–0.007
	Longitudinal	0.000	0.914	− 0.006–0.005
*circCDYL*	Cross-sectional	− 0.001	0.665	− 0.004–0.003
	Longitudinal	0.000	0.828	− 0.004–0.003
*circDEF6*	Cross-sectional	0.000	0.903	− 0.005–0.006
	Longitudinal	0.002	0.599	− 0.004–0.008
*circEP300*	Cross-sectional	− 0.004	0.060	− 0.007–0.000
	Longitudinal	− 0.003	0.112	− 0.007–0.001
*circFNDC3B*	*Cross-sectional*	**0.004**	**0.039**	**0.000**–**0.008**
	*Longitudinal*	**0.004**	**0.038**	**0.000**–**0.008**
*circFOXO3*	Cross-sectional	0.002	0.402	− 0.002–0.005
	Longitudinal	0.000	0.834	− 0.004–0.003
*circITGAX*	Cross-sectional	0.000	0.997	− 0.004–0.004
	Longitudinal	− 0.001	0.658	− 0.006–0.004
*circMETTL3*	Cross-sectional	− 0.003	0.139	− 0.007–0.001
	Longitudinal	− 0.001	0.680	− 0.005–0.003
*circMIB1*	Cross-sectional	0.000	0.906	− 0.003–0.003
	Longitudinal	0.002	0.305	− 0.002–0.005
*circPLEKHM1*	Cross-sectional	0.000	0.799	− 0.002–0.003
	Longitudinal	− 0.001	0.614	− 0.003–0.002
*circXPO7*	Cross-sectional	− 0.003	0.236	− 0.008–0.002
	Longitudinal	− 0.004	0.139	− 0.009–0.001
*circZC3H18*	Cross-sectional	0.001	0.761	− 0.003–0.004
	Longitudinal	− 0.002	0.374	− 0.006–0.002

Beta coefficients, *p* values and 95% confidence intervals (95% CI) are given for associations between circRNA expression and hand grip strength. Three hundred six individuals were assessed. Associations were assessed cross-sectionally (expression data FU3 and clinical outcome FU3) and longitudinally (expression data FU3, clinical outcome FU4). All associations identified here were nominal only and are given in bold

### circRNAs are differentially expressed in early passage and late passage cells

Twelve of 15 circRNAs tested were expressed in astrocytes, endothelial cells, fibroblasts or astrocytes. Seven (58%) of these demonstrated differential expression between early and late passage cells of one or more cell type (Table 5). *circAFF1* and *circFOXO3* demonstrated associations in more than one cell type although direction of effect was concordant only for *circFOXO3* (in cardiomyocytes and fibroblasts). *circCDYL*, *circEP300*, *circMIB1*, *circZC3H18* and *circMETTL3* were differentially expressed in only one cell type. *circBCL11B*, *circDEF6* and *circITGAX* were not expressed in any cell type tested*.*

**Table 5:** circRNA expression in early and late passage primary human cells

circRNA	Median (IQR)		*p* value
	Early passage	Late passage	
Astrocytes			
*circAFF1*	**0.58 (0.55–0.68)**	**0.84 (0.79–1.09)**	**0.040**
*circASAP1*	1.39 (0.97–1.48)	1.22 (1.18–1.36)	0.878
*circATP6V0A1*	1.60 (1.14–1.87)	1.14 (1.05–1.41)	0.229
*circCDYL*	**0.71 (0.67–0.74)**	**0.90 (0.90–0.93)**	**0.001**
*circEP300*	1.01 (0.95–1.04)	1.05 (1.00–1.07)	0.329
*circFNDC3B*	0.96 (0.85–1.10)	1.38 (1.20–1.48)	0.059
*circFOXO3*	0.88 (0.80–0.89)	0.89 (0.80–0.98)	0.646
*circMETTL3*	0.97(0.92–1.08)	0.69 (0.66–1.02)	0.180
*circMIB1*	**0.71(0.69–0.86)**	**1.03 (0.99–1.04)**	**0.008**
*circPLEKHM1*	1.05 (1.00–1.09)	0.76 (0.61–1.17)	0.306
*circXPO7*	1.25 (1.12–1.58)	1.54 (0.81–1.62)	0.987
*circZC3H18*	1.50 (0.67–2.24)	0.88 (1.00–1.07)	0.346
Cardiomyocytes			
*circAFF1*	1.15 (1.09–1.26)	1.42 (1.04–1.52)	0.357
*circASAP1*	0.74 (0.71–1.05)	0.84 (0.83–1.02)	0.643
*circATP6V0A1*	0.57 (0.44–0.80)	0.41 (0.39–0.54)	0.249
*circCDYL*	1.47 (1.29–1.48)	1.42 (1.25–1.64)	0.855
*circEP300*	1.27 (1.02–1.48)	1.10 (0.84–1.45)	0.596
*circFNDC3B*	1.03 (0.83–1.09)	1.93 (0.96–1.97)	0.139
*circFOXO3*	**1.00(0.99–1.07)**	**0.82 (0.79–0.92)**	**0.015**
*circMETTL3*	0.88(0.69–0.99)	0.66 (0.63–0.79)	0.186
*circMIB1*	0.96 (0.81–1.02)	1.16 (0.97–1.25)	0.129
*circPLEKHM1*	0.85 (0.84–1.05)	0.82(0.71–1.22)	0.983
*circXPO7*	0.89 (0.74–0.94)	1.32 (0.75–1.63)	0.227
*circZC3H18*	0.83 (0.63–1.43)	0.85 (0.77–1.12)	0.862
Endothelial cells			
*circAFF1*	0.94 (0.91–1.27)	1.07 (0.49–1.11)	0.548
*circASAP1*	1.03 (0.94–1.28)	1.69 (0.68–1.76)	0.467
*circATP6V0A1*	0.37 (0.16–0.58)	0.48 (0.48–0.48)	0.821
*circCDYL*	0.90 (0.76–1.11)	0.89 (0.84–1.02)	0.942
*circEP300*	0.99 (.75–1.45)	0.58 (0.53–0.80)	0.128
*circFNDC3B*	1.74 (1.49–3.18)	7.84 (3.18–9.97)	0.080
*circFOXO3*	0.38 (0.20–1.98)	0.14 (0.04–0.22)	0.275
*circMETTL3*	1.02 (0.54–1.08)	0.39 (0.39–0.56)	0.072
*circMIB1*	1.36 (0.98–1.54)	1.11 (1.03–1.43)	0.640
*circPLEKHM1*	1.02 (0.99–1.42)	1.47(0.83–4.85)	0.380
*circXPO7*	0.97 (0.72–1.18)	0.87 (0.31–1.22)	0.620
*circZC3H18*	**1.02 (0.98–1.15)**	**1.51 (1.51–1.51)**	**0.047**
Fibroblasts			
*circAFF1*	**1.06 (0.95–1.16)**	**0.58 (0.52–0.65)**	**0.003**
*circASAP1*	0.51 (0.38–1.07)	1.05 (0.85–1.10)	0.196
*circATP6V0A1*	1.39 (1.00–1.41)	1.10 (0.46–1.35)	0.375
*circCDYL*	1.13 (0.72–1.17)	0.90 (0.81–1.06)	0.640
*circEP300*	**0.96 (0.78–0.98)**	**0.38 (0.38–0.69)**	**0.023**
*circFNDC3B*	0.50(0.48–0.94)	0.90 (0.85–0.91)	0.182
*circFOXO3*	**1.91 (1.72–2.01)**	**1.60 (1.47–1.61)**	**0.025**
*circMETTL3*	**1.23(1.00–1.26)**	**1.39 (1.58–1.66)**	**0.030**
*circMIB1*	1.20 (1.14–1.47)	0.85 (0.69–1.11)	0.072
*circPLEKHM1*	1.00(0.90–1.00)	0.84 (0.79–1.14)	0.716
*circXPO7*	1.03(0.48–1.08)	0.57 (0.39–1.18)	0.645
*circZC3H18*	0.93 (0.72–1.21)	0.74 (0.53–0.94)	0.432

Results reaching statistical significance are indicated in bold typeface

*IQR* interquartile range

### Differential expression of circRNAs between mice of different median strain longevities

In silico analyses suggested that four circRNAs (*circFoxo3*, *circMib1*, *circPlekhm1* and *circXpo7*) may have conserved back-spliced junction in the mouse. Associations with longevity were then assessed in spleen and muscle tissue from young (6 months) and old (20–22 months) mouse strains of six different median strain longevities. *circMib1* and *circXpo7* were expressed only in spleen, whereas *circFoxo3 and circPlekhm1* were expressed in both tissues (Table 6). The expression of *circPlekhm1* demonstrated a nominal positive correlation with median lifespan in young and old (*β* = 0.0013, *P* = 0.016) as well as in spleen of young mice (*β* = 0.0025, *P* = 0.017), although these were not significant after adjustment for multiple testing (threshold *P* = 0.013). No associations were seen between muscle circRNA expression levels and median strain longevity.

**Table 6:** Differential expression of conserved circRNAs in mice of differential median strain longevities

circRNA	Tissue	β-Coefficient	*p* value	95% CI	
*circFoxo3*	Muscle	0.00	0.403	− 0.0010	0.0024
	Young (muscle)	0.0001	0.936	− 0.0028	0.0031
	Old (muscle)	0.0008	0.478	− 0.0015	0.0031
	Spleen	− 0.0003	0.815	− 0.0027	0.0021
	Young (spleen)	0.0002	0.922	− 0.0039	0.0043
	Old (spleen)	− 0.0005	0.757	− 0.0037	0.0027
*circMib1*	Muscle	ND	ND	ND	ND
	Young (muscle)	ND	ND	ND	ND
	Old (muscle)	ND	ND	ND	ND
	Spleen	0.0001	0.924	− 0.0023	0.0026
	Young (spleen)	− 0.0018	0.150	− 0.0044	0.0008
	Old (spleen)	0.0021	0.299	− 0.0020	0.0062
*circPlekhm1*	Muscle	0.0003	.813	− 0.0022	0.0028
	Young (muscle)	− 0.0022	0.161	− 0.0054	0.0010
	Old (muscle)	0.0016	0.365	− 0.0020	0.0053
	*Spleen*	**0.0013**	**0.016**	**0.0002**	**0.0024**
	*Young (spleen)*	**0.0025**	**0.017**	**0.0005**	**0.0046**
	Old (spleen)	0.00001	0.967	− 0.0008	0.0009
*circXpo7*	Muscle	ND	ND	ND	ND
	Young (muscle)	ND	ND	ND	ND
	Old (muscle)	ND	ND	ND	ND
	Spleen	0.0009	0.509	− 0.0019	0.0038
	Young (spleen)	0.0003	0.894	− 0.0040	0.0045
	Old (spleen)	0.0020	0.333	− 0.0023	0.0063

circRNA expression is reported here in relation to median strain longevity. Data are assessed separately for young and old animals of each strain. *N* = 67 (muscle); 90 (spleen). Results reaching statistical significance are indicated in bold typeface

*IQR* interquartile range, *ND* not detected

## Discussion

Circular RNAs (circRNAs) are an emerging class of regulatory RNA molecule thought to play a role in human disease (Haque and Harries 2017). These molecules have no free ends, and as such are exonuclease resistant. circRNAs accumulate in aged organisms (Gruner et al. 2016) and have been suggested to play a role in cellular senescence (Du et al. 2017; Du et al. 2016). We hypothesised that the human circRNAome may differ in aged humans compared with younger subjects and that these changes may also be associated with cellular senescence or with longevity in animal models. We identified > 2000 circRNAs in total RNA from human blood, some of which were expressed exclusively in samples from older donors. GSEA pathways enrichment analysis of genes hosting the top 10% most abundant circRNAs in elderly donors suggested that pathways involved in phagocytosis, circadian regulation, cancer pathways and golgi-associated vesicles were the most enriched in these genes. We demonstrated that three circRNAs (*circDEF6*, *circFOXO3* and *circEP300*) were associated with measures of parental longevity, and one (*circFNDC3B*) was associated with hand grip strength both longitudinally and cross-sectionally. Furthermore, 7 of 12 circRNAs expressed in human senescent cells of different cell types demonstrated dysregulated expression in one or more cell type and 1 of 4 circRNAs demonstrating conserved expression were associated with median strain longevity in spleen tissue from young mice. These findings are consistent with the hypothesis that some circRNAs have roles in molecular aging and the determination of mammalian aging phenotypes.

circRNAs generated from the *FOXO3* and *EP300* genes were negatively associated with measures of human parental longevity and also demonstrated dysregulated expression in human senescent cells. circRNAs deriving from the *FOXO3* gene have previously been demonstrated to regulate cell cycle when manipulated by gene knockdown in mouse embryonic fibroblasts, cardiac fibroblasts or mammary cancer cell lines (Du et al. 2016). Furthermore, *FOXO3* circular RNAs also demonstrate elevated expression and association with cellular senescence in the heart tissue of mice and humans (Du et al. 2017). It is not clear whether the previously reported circular *FOXO3* transcripts have the same structure as the one we have identified, since previous studies do not give its exon structure. A circRNA from the *FOXO3* gene identical to the one we have identified has also previously been demonstrated to inhibit myoblast differentiation in mouse cells (Li et al. 2019). Genetic variation in the *FOXO3* gene itself has previously been associated with extreme longevity (Flachsbart et al. 2017; Fuku et al. 2016) and has also been associated with maintenance of telomere length (Davy et al. 2018).

circRNAs deriving from the *EP300* gene have not been previously reported. *EP300* encodes the repressor histone acetyltransferase protein p300, which also has roles as a transcriptional corepressor protein. EP300 has been implicated in modulation of *FOXO3* activity (Mahmud et al. 2019) and in antagonism of the FOX03a/SIRT1 signalling axis (Jeung et al. 2016). Inhibition of EP300 has been shown to mimic calorific restriction in human and mouse cells (Pietrocola et al. 2018); calorific restriction is of course a well-known modifier of lifespan in many species (Austad 1989; Hansen et al. 2008; Kapahi et al. 2004; Mitchell et al. 2010). This protein is also a master regulator of autophagy, which is a pivotal factor in stem cell maintenance and evasion of cellular senescence (Vijayakumar and Cho 2019).

*circFNDC3B* was positively associated with hand grip strength. Although these associations were nominal only, they were present both cross-sectionally and longitudinally. An average person may lose ~ 20–40% of skeletal muscle mass as well as muscle strength from by the time they reach 80 years of age (Carmeli et al. 2002; Doherty 2003) and decline in skeletal muscle strength is predictive of disability and mortality in humans (Giampaoli et al. 1999; Rantanen et al. 1999; Rantanen et al. 2012). Circular RNAs originating from this gene have been reported previously, and suggested to possess tumour suppressor activity (Liu et al. 2018).

The results generated from our mouse data suggest that *circPlekhm1*, which was associated with median strain longevity, may drive longevity, rather than being consequential to it, since the associations are present in the spleen RNA of young mice alone. The *Plekhm1* gene encodes a multivalent adaptor protein that integrates endocytic and autophagic pathways at the lysosome (McEwan and Dikic 2015). Its role in lifespan may therefore stem from moderation of lysosomal trafficking since lysosomes play a critical part in successful aging and longevity (Carmona-Gutierrez et al. 2016; Simonsen et al. 2007).

Our study has both strengths and weaknesses. It represents one of the first circRNA profiles in aging human peripheral blood and provides data not only population-level epidemiological evidence for a role in human aging phenotypes, or mammalian lifespan, but also in vitro evidence that some circRNA may influence cell senescence phenotypes. Weaknesses include a relatively low power to detect effects of in the population study, which might be attributed to the biological variation in circRNA levels and limitations in samples size and power. Nevertheless, we were able to identify some interesting associations, which likely represent the largest effects. Future work could include validation of epidemiological data in larger sample sets and also functional delineation of the molecular effects of the circRNA in question. Our data provide evidence that circRNAs may play an important role in the determination of mammalian aging phenotypes. circRNAs are inherently stable, due to their exonuclease resistance, and are found not only in tissues relevant to human diseases, but also in the circulation, raising the possibility that they may prove useful as biomarkers of disease or targets for molecular therapies in the future.

## Electronic supplementary material

ESM 1 (DOCX 13 kb)

ESM 2 (DOCX 12 kb)

ESM 3 (PDF 2209 kb)

ESM 4 (PNG 6293 kb)

High Resolution Image (TIF 1267 kb)
