# Supplementary material for: circRNAs expressed in human peripheral blood are associated with human aging phenotypes, cellular senescence and mouse lifespan
Source: GeroScience. 2019 Dec 6;42(1):183–99. doi: 10.1007/s11357-019-00120-z (PMC7031184; doi:10.1007/s11357-019-00120-z)
Supplement: Supplementary file 1 — (DOCX 13 kb) [file 11357_2019_120_MOESM1_ESM.docx]

**Online Resource 1: Assay information for age-associated circRNA assessed in this study.**

|  | |  |  |  |
| --- | --- | --- | --- | --- |
|  |  | |  |  |
| **CircRNA** | **Forward primer** | | **Probe** | **Reverse primer** |
| *CircAFF1* | CCTGCCAAAGCCAAGCT | | TCTCAGTCAGTTGAGTTTGT | AGCAGGTTTCTGTCGTCATTGT |
| *CircASAP1* | AGGAGGAAGTGTTCAGTCAAGAATG | | CACATGCCACATTTCT | ACTTATCAGCTGTTTTCAAGCCATCT |
| *CircATP6V0A1* | CGCCGTCAGTATTTGAGGAGAAA | | CTTCTTGAAATAGCAAATGC | AGCCAAACAAAGAGGTCATGAAGAT |
| *CircBCL11B* | AAAGGCATCTGTCCCAAGCA | | CAGCCTCTGCAATGTT | GCGGCCTCCACATGGT |
| *CircCDYL* | CATGGCCACAGGCTTAGCT | | CAATCCTTTCAACCTTTCCC | CGAACCAAATACTCTGTCTTCCCTTTT |
| *CircDEF6* | GGGAGTGAAGAGTGCAAAGAGAAA | | TCCACCTCCACGCAGCAG | GCTGAGTACCTTTTTCAGCAGGTAT |
| *CircEP300* | GTTGCATATGCTCGGAAAGTTGAAG | | CATTCCCATTCGATTGTTTG | GCTGTCCAGGATTCTGAGTATATGG |
| *CircFNDC3B* | AGCCCAAAGTCGAATGATTCAGA | | TTGCAAGGTGATTGAAGATA | CCGGCGGACTCCAGTAC |
| *CircFOXO3* | AGGCTGAAGGATCACTGAGGAA | | ATGGAGTTCTGCTTTGCC | CGACTATGCAGTGACAGGTTGT |
| *CircITGAX* | GAGGATGAAGGCCGAAGTCA | | CCGTACCTGAGTCCCC | TCGAAGGAGCTACTGCTTGTG |
| *CircMETTL3* | GAACCAACAGTCCACTAAGGAACAA | | CAGAGCAAGAAGATCTAC | ACAATGCTGCCTCTGGATTCC |
| *CircM1B1* | GGCATTGATGAAGATCATGACATTGT | | ATGCTTGATGCCTATTGCC | TTGCTGGCGGCAGGTAT |
| *CircPLEKHM1* | CTGGGCACAGCAAATGCT | | CTGCAAGAACACATCATC | CGTCAGGTGCTCCAACTCT |
| *CircXPO7* | TGTTGATGGTGTTAAACGAATACTGGAA | | CCCACAGGCAGACACC | AGAGGCTATTTTTCTGTGCTTGGT |
| *CircZCH3H18* | GGAGCGGGAGAAGGAGAAG | | CAGCCGCCAAGACTCG | CCAAAACCGCTCAATTTCATAGTCATAA |
|  |  | |  |  |
|  |  | |  |  |
| **Endogenous controls** | **Assay ID** | | **Supplier** |  |
| *IP08* | Hs00183533_m1 | | Thermofisher Scientific |  |
| *P0L2RA* | Hs00172187_m1 | | Thermofisher Scientific |  |
| *TFRC* | Hs00174609_m1 | | Thermofisher Scientific |  |
