# Supplementary material for: circRNAs expressed in human peripheral blood are associated with human aging phenotypes, cellular senescence and mouse lifespan
Source: GeroScience. 2019 Dec 6;42(1):183–99. doi: 10.1007/s11357-019-00120-z (PMC7031184; doi:10.1007/s11357-019-00120-z)
Supplement: Supplementary file 2 — (DOCX 12 kb) [file 11357_2019_120_MOESM2_ESM.docx]

**Online Resource 2: Assay details for mouse circRNAs assessed in this work.**

| **CircRNA** | **Forward primer** | **Probe** | **Reverse primer** |
| --- | --- | --- | --- |
| *CircMib1* | AACTACAACTCGAACCGTCTG | CCAAGTGGCAATAGGCATCAAGCA | CGGCAGGTATCACACATAGTT |
| *CircPlekhm1* | TCTGAGGAACCCATGTCCTAT | CCGACAGGTCTCTGCAAGAACACA | AAGACCAGGTGCTCCAAATC |
| *CircXpo7* | GGCCAACTTTCTCTCTCATCTT | TCCACAGGCAGACACAACTCATCC | GTCTCGGAAAGAAGAGGCTATTT |
| *CircFoxo3* | CTGAAGGATCACTGAGGAAAGG | TGGAGTTCTGCTTGCCCATTTCC | TCATTCTGAACGCGCATGA |
|  |  |  |  |
| **Endogenous controls** | **Assay ID** | **Supplier** |  |
| *Ip08* | Mm.PT.39a.22214844 | Integrated DNA Technologies |  |
| *Pol2ra* | Mm.PT.39a.22214849 | Integrated DNA Technologies |  |
| *Tfrc* | MM.PT.39a.22214833.g | Integrated DNA Technologies |  |
