# Supplementary material for: circRNAs expressed in human peripheral blood are associated with human aging phenotypes, cellular senescence and mouse lifespan
Source: GeroScience. 2019 Dec 6;42(1):183–99. doi: 10.1007/s11357-019-00120-z (PMC7031184; doi:10.1007/s11357-019-00120-z)
Supplement: Supplementary file 3 — (PDF 2209 kb) [file 11357_2019_120_MOESM3_ESM.pdf]

<u>Online Resource 3: CircRNA profile in ageing human peripheral blood.</u> CircRNA profiles were generated from 2 pooled samples of human peripheral blood. Pool 1 consisted of RNA samples from 20 young individuals (median age 33 years) and 20 aged individuals (median age 87 years) using CircleSeq. The table gives the host transcript identity, the host transacript NM_ number, the chromosomal position and the ranked abundance of each from those expressed exclusively in the young (rows 4 to 434), those expressed in both old and young ranked by fold change (rows 436 to 619) and those expressed exclusively in old samples (rows 621 to 2122). Assays to unique circRNA backspliced junctions were designed to transcripts marked in bold type. These represented the top 5 most abunant circRNA expressed exclusively in young, the top 5 circRNA expressed exclusively in old, and 5 which demonstrated the most dysregulation between young and old pools. In some cases, we were ubnable to design assays to circRNAs in these categories, because of sequence and assay design constraints. In these cases, we designed assays to the next most abundent/dysregulated circRNA.

Transcript Id	Gene Id	chromosome	start	stop	raw_count (young)	BPM (young)	raw_count Old	BPA (old)	Fold change
**NM_000887.12.10**	**ITGAX**	**chr16**	**31373156**	**31374074**	**13**	**357.6646**	**N/A**	**N/A**	**N/A**
**NM_014798.4.4**	**PLEKHM1**	**chr17_ctg5_hap1**	**167528**	**168155**	**9**	**247.614**	**N/A**	**N/A**	**N/A**
NM_007166.12.9	*PICALM*	*chr11*	*85707868*	*85714494*	*9*	*247.614*	*N/A*	*N/A*	*N/A*
**NM_022047.5.4**	**DEF6**	**chr6**	**35280078**	**35280549**	**7**	**192.5886**	**N/A**	**N/A**	**N/A**
**NM_001130021.17.16**	**ATP6V0A1**	**chr17**	**40652724**	**40653322**	**7**	**192.5886**	**N/A**	**N/A**	**N/A**
**NM_018482.13.10**	**ASAP1**	**chr8**	**131164981**	**131181313**	**7**	**192.5886**	**N/A**	**N/A**	**N/A**
NM_000333.4.3	ATXN7	chr3	63898263	63898901	6	165.076	N/A	N/A	N/A
NM_032959.8.7	POLR2J2	chr7	102279160	102279646	6	165.076	N/A	N/A	N/A
NM_004824.2.2	CDYL	chr6	4891946	4892613	6	165.076	N/A	N/A	N/A
NR_002939.9.6	-	chr16	29367107	29376380	5	137.5633	N/A	N/A	N/A
NM_001242357.4.3	NCR1	chr19	55420603	55421425	5	137.5633	N/A	N/A	N/A
NM_016004.8.7	IFT52	chr20	42242489	42247666	5	137.5633	N/A	N/A	N/A
NM_152487.6.2	TMEM56	chr1	95609446	95639445	5	137.5633	N/A	N/A	N/A
NM_001146032.20.16	FCHO2	chr5	72354259	72373320	5	137.5633	N/A	N/A	N/A
NM_198925.4.2	SEMA4B	chr15	90760670	90763123	5	137.5633	N/A	N/A	N/A
NM_001195733.5.4	PIP5K1C	chr19	3660963	3661999	5	137.5633	N/A	N/A	N/A
NM_001282679.17.16	GAPVD1	chr9	128099296	128099870	5	137.5633	N/A	N/A	N/A
NM_002881.4.2	RALB	chr2	121036193	121047333	4	110.0507	N/A	N/A	N/A
NM_007280.4.3	OIP5	chr15	41605470	41611978	4	110.0507	N/A	N/A	N/A
NM_020765.9.8	UBR4	chr1	19519926	19523759	4	110.0507	N/A	N/A	N/A

NM_022039.6.2	FBXW4	chr10	103384501	103436193	4	110.0507	N/A	N/A	N/A
NM_018449.12.9	UBAP2	chr9	33953282	33963789	4	110.0507	N/A	N/A	N/A
NM_030802.5.4	FAM117A	chr17	47797121	47797798	4	110.0507	N/A	N/A	N/A
NM_000124.7.6	ERCC6	chr10	50708583	50714058	4	110.0507	N/A	N/A	N/A
NM_015179.6.4	RRP12	chr10	99150179	99153517	4	110.0507	N/A	N/A	N/A
NM_014282.5.2	HABP4	chr9	99220660	99233376	4	110.0507	N/A	N/A	N/A
NM_001167681.4.3	FLI1	chr11	128628009	128638167	4	110.0507	N/A	N/A	N/A
NM_213662.4.2	STAT3	chr17	40497576	40500557	4	110.0507	N/A	N/A	N/A
NM_001146032.20.19	FCHO2	chr5	72370568	72373320	4	110.0507	N/A	N/A	N/A
NM_001130420.25.23	SMARCC2	chr12	56563312	56563992	4	110.0507	N/A	N/A	N/A
NM_001202504.4.2	CTDP1	chr18	77455224	77457988	4	110.0507	N/A	N/A	N/A
NR_028075.10.9	-	chr17	1540002	1540356	4	110.0507	N/A	N/A	N/A
NM_016293.10.10	BIN2	chr12	51685374	51686128	4	110.0507	N/A	N/A	N/A
NM_023018.7.6	NADK	chr1	1686812	1687782	4	110.0507	N/A	N/A	N/A
NM_000293.7.2	PHKB	chr16	47531309	47581459	4	110.0507	N/A	N/A	N/A
NM_015226.18.11	CLEC16A	chr16	11114049	11154879	4	110.0507	N/A	N/A	N/A
NM_207123.10.7	GAB1	chr4	144378832	144387378	4	110.0507	N/A	N/A	N/A
NM_152415.4.2	VPS37A	chr8	17123415	17126465	4	110.0507	N/A	N/A	N/A
NR_103473.6.5	-	chr1	59811918	59844509	4	110.0507	N/A	N/A	N/A
NM_001144072.4.2	UBAC2	chr13	99890680	99896878	4	110.0507	N/A	N/A	N/A
NM_001146054.4.2	SNCA	chr4	90743396	90756843	4	110.0507	N/A	N/A	N/A
NM_018359.4.3	UFSP2	chr4	186339594	186339924	3	82.53799	N/A	N/A	N/A
NM_001002762.5.4	DNAJB12	chr10	74100546	74100928	3	82.53799	N/A	N/A	N/A
NM_016581.4.3	ECSIT	chr19	11623870	11625036	3	82.53799	N/A	N/A	N/A
NM_001144823.19.15	DENND4A	chr15	65993388	65998562	3	82.53799	N/A	N/A	N/A
NM_174917.8.2	ACSF3	chr16	89164998	89199670	3	82.53799	N/A	N/A	N/A
NM_020774.10.2	MIB1	chr18	19345732	19383975	3	82.53799	N/A	N/A	N/A
NM_080627.14.2	SOGA1	chr20	35421651	35467844	3	82.53799	N/A	N/A	N/A
NM_001008660.7.5	PICALM	chr11	85722072	85726006	3	82.53799	N/A	N/A	N/A
NM_016426.9.8	GTSE1	chr22	46719086	46722551	3	82.53799	N/A	N/A	N/A
NM_004286.9.5	GTPBP1	chr22	39117746	39123353	3	82.53799	N/A	N/A	N/A
NM_018062.5.2	FANCL	chr2	58449076	58459247	3	82.53799	N/A	N/A	N/A
NM_015435.4.3	RNF19A	chr8	101287180	101300495	3	82.53799	N/A	N/A	N/A

NM_013448.15.12	BAZ1A	chr14	35252967	35262127	3	82.53799	N/A	N/A	N/A
NM_002061.6.3	GCLM	chr1	94360169	94367225	3	82.53799	N/A	N/A	N/A
NM_001172219.10.9	SCMH1	chr1	41536266	41541123	3	82.53799	N/A	N/A	N/A
NM_101395.4.3	DYRK1A	chr21	38792600	38845182	3	82.53799	N/A	N/A	N/A
NM_001242599.5.4	RBM39	chr20	34317233	34320057	3	82.53799	N/A	N/A	N/A
NM_001009905.4.2	B3GNTL1	chr17	80992910	81006661	3	82.53799	N/A	N/A	N/A
NR_037615.4.3	-	chr12	70687850	70704797	3	82.53799	N/A	N/A	N/A
NM_001258273.14.13	MLH1	chr3	37081676	37083822	3	82.53799	N/A	N/A	N/A
NM_138341.9.4	TMEM116	chr12	112370389	112381173	3	82.53799	N/A	N/A	N/A
NM_022836.3.3	DCLRE1B	chr1	114450630	114450813	3	82.53799	N/A	N/A	N/A
NM_032412.2.2	CYSTM1	chr5	139574030	139574237	3	82.53799	N/A	N/A	N/A
NM_007010.13.10	DDX52	chr17	35979812	35981565	3	82.53799	N/A	N/A	N/A
NM_001429.5.3	EP300	chr22	41521867	41526007	3	82.53799	N/A	N/A	N/A
NM_006561.9.7	CELF2	chr10	11312628	11330515	3	82.53799	N/A	N/A	N/A
NM_020314.14.13	C16orf62	chr16	19627435	19628130	3	82.53799	N/A	N/A	N/A
NM_017643.6.3	MBTD1	chr17	49294694	49302570	3	82.53799	N/A	N/A	N/A
NM_014972.12.6	TCF25	chr16	89958600	89967202	3	82.53799	N/A	N/A	N/A
NM_001283032.23.19	SCFD1	chr14	31185129	31204064	3	82.53799	N/A	N/A	N/A
NM_001282886.3.2	EIF4E3	chr3	71759562	71777774	3	82.53799	N/A	N/A	N/A
NM_015335.4.3	MED13L	chr12	116534473	116549317	3	82.53799	N/A	N/A	N/A
NM_015033.15.12	FNBP1	chr9	132662243	132671278	3	82.53799	N/A	N/A	N/A
NM_024874.6.4	KIAA0319L	chr1	35936463	35944813	3	82.53799	N/A	N/A	N/A
NM_001127183.8.2	CFLAR	chr2	201994451	202014558	3	82.53799	N/A	N/A	N/A
NM_005819.7.2	STX6	chr1	180953812	180974599	3	82.53799	N/A	N/A	N/A
NM_024561.9.3	NAA16	chr13	41892941	41910892	3	82.53799	N/A	N/A	N/A
NM_006836.22.10	GCN1	chr12	120599293	120614020	3	82.53799	N/A	N/A	N/A
NM_001164759.4.2	PRKAR1B	chr7	716865	751164	3	82.53799	N/A	N/A	N/A
NM_005993.7.3	TBCD	chr17	80721840	80739597	3	82.53799	N/A	N/A	N/A
NM_001265612.42.40	CNOT1	chr16	58565861	58568299	3	82.53799	N/A	N/A	N/A
NM_015013.8.3	KDM1A	chr1	23376879	23385660	3	82.53799	N/A	N/A	N/A
NM_032206.35.32	NLRC5	chr16	57099123	57101740	3	82.53799	N/A	N/A	N/A
NR_073585.4.2	-	chr17	61655830	61657304	3	82.53799	N/A	N/A	N/A
NM_024948.8.2	FAM188A	chr10	15875628	15889942	3	82.53799	N/A	N/A	N/A

NM_198896.6.4	RAB6A	chr11	73418464	73429763	3	82.53799	N/A	N/A	N/A
NM_014688.14.11	USP6NL	chr10	11523768	11527910	3	82.53799	N/A	N/A	N/A
NM_001271635.4.3	CNOT9	chr2	219447693	219449444	3	82.53799	N/A	N/A	N/A
NM_032959.8.5	POLR2J2	chr7	102279160	102281220	3	82.53799	N/A	N/A	N/A
NM_001163735.15.13	MYO19	chr17	34864900	34867294	3	82.53799	N/A	N/A	N/A
NM_032156.12.9	CAPRIN2	chr12	30873744	30879022	3	82.53799	N/A	N/A	N/A
NM_000086.6.3	CLN3	chr16	28498776	28500707	3	82.53799	N/A	N/A	N/A
NM_032213.8.5	ELMOD3	chr2	85595808	85598685	3	82.53799	N/A	N/A	N/A
NM_001256865.5.3	DNAJC6	chr1	65830317	65831879	3	82.53799	N/A	N/A	N/A
NM_003262.7.3	SEC62	chr3	169694733	169706147	2	55.02533	N/A	N/A	N/A
NM_004814.5.3	SNRNP40	chr1	31754220	31764853	2	55.02533	N/A	N/A	N/A
NM_001178137.7.4	CPEB3	chr10	93870832	93940776	2	55.02533	N/A	N/A	N/A
NM_001243249.7.6	NPRL3	chr16	148142	150507	2	55.02533	N/A	N/A	N/A
NM_024789.9.8	MFSD13A	chr10	104233326	104233769	2	55.02533	N/A	N/A	N/A
NR_033676.3.2	-	chrX	106410878	106418423	2	55.02533	N/A	N/A	N/A
NM_007368.4.4	RASA3	chr13	114806475	114806570	2	55.02533	N/A	N/A	N/A
NM_001127391.11.10	ALS2CR12	chr2	202172241	202173973	2	55.02533	N/A	N/A	N/A
NM_001135190.5.3	ARAP1	chr11	72423240	72424288	2	55.02533	N/A	N/A	N/A
NM_001172639.15.13	CELF1	chr11	47493742	47497073	2	55.02533	N/A	N/A	N/A
NM_000052.13.11	ATP7A	chrX	77270158	77275895	2	55.02533	N/A	N/A	N/A
NM_014173.8.7	BABAM1	chr19	17387303	17387718	2	55.02533	N/A	N/A	N/A
NM_184234.11.7	RBM39	chr20	34302106	34313077	2	55.02533	N/A	N/A	N/A
NM_001080432.8.5	FTO	chr16	53907697	53968021	2	55.02533	N/A	N/A	N/A
NM_001097615.4.2	POLR2J3	chr7	102207443	102210371	2	55.02533	N/A	N/A	N/A
NM_024074.3.2	TMEM38A	chr19	16790794	16791392	2	55.02533	N/A	N/A	N/A
NM_001009.4.3	RPS5	chr19	58904342	58904854	2	55.02533	N/A	N/A	N/A
NM_014949.10.8	KIAA0907	chr1	155891165	155893478	2	55.02533	N/A	N/A	N/A
NM_025090.7.6	USP36	chr17	76818018	76823429	2	55.02533	N/A	N/A	N/A
NM_002973.18.16	ATXN2	chr12	111923074	111924628	2	55.02533	N/A	N/A	N/A
NM_198435.6.3	AURKA	chr20	54956488	54961589	2	55.02533	N/A	N/A	N/A
NM_003161.12.10	RPS6KB1	chr17	58012553	58013902	2	55.02533	N/A	N/A	N/A
NM_032410.5.3	HOOK3	chr8	42780699	42798588	2	55.02533	N/A	N/A	N/A
NM_173083.10.9	LIN9	chr1	226453233	226454033	2	55.02533	N/A	N/A	N/A

NM_002209.15.14	ITGAL	chr16	30507417	30507887	2	55.02533	N/A	N/A	N/A
NM_004728.2.2	DDX21	chr10	70719561	70720005	2	55.02533	N/A	N/A	N/A
NM_002756.9.2	MAP2K3	chr17	21201724	21208440	2	55.02533	N/A	N/A	N/A
NM_005761.4.2	PLXNC1	chr12	94562928	94580249	2	55.02533	N/A	N/A	N/A
NM_053043.5.3	RBM33	chr7	155465560	155473602	2	55.02533	N/A	N/A	N/A
NM_212554.4.2	METTL10	chr10	126463243	126478907	2	55.02533	N/A	N/A	N/A
NM_020122.6.4	KCMF1	chr2	85270686	85276771	2	55.02533	N/A	N/A	N/A
NM_001013837.3.2	MAD1L1	chr7	2269618	2270359	2	55.02533	N/A	N/A	N/A
NM_139057.6.3	ADAMTS17	chr15	100801683	100871259	2	55.02533	N/A	N/A	N/A
NM_001110781.8.5	SLC35E2B	chr1	1599765	1603068	2	55.02533	N/A	N/A	N/A
NM_001243403.18.13	CLEC16A	chr16	11133610	11154879	2	55.02533	N/A	N/A	N/A
NM_001134335.6.3	EIF2AK1	chr7	6085701	6089676	2	55.02533	N/A	N/A	N/A
NM_032226.2.2	ZCCHC7	chr9	37126308	37126939	2	55.02533	N/A	N/A	N/A
NM_001144823.3.2	DENND4A	chr15	66048477	66053776	2	55.02533	N/A	N/A	N/A
NM_001005366.9.7	KDM2B	chr12	121958787	121972495	2	55.02533	N/A	N/A	N/A
NM_001029880.10.5	SFMBT2	chr10	7269816	7327916	2	55.02533	N/A	N/A	N/A
NM_138384.9.7	MTG1	chr10	135215032	135216277	2	55.02533	N/A	N/A	N/A
NM_138571.4.3	HINT3	chr6	126293404	126296119	2	55.02533	N/A	N/A	N/A
NM_001040033.4.3	CD53	chr1	111434013	111435155	2	55.02533	N/A	N/A	N/A
NR_103729.8.4	-	chr2	173429230	173435552	2	55.02533	N/A	N/A	N/A
NM_001037144.13.13	CNTROB	chr17	7849045	7849304	2	55.02533	N/A	N/A	N/A
NM_004618.8.4	TOP3A	chr17	18205198	18210280	2	55.02533	N/A	N/A	N/A
NM_145648.2.2	SLC15A4	chr12	129299319	129299615	2	55.02533	N/A	N/A	N/A
NM_020732.4.4	ARID1B	chr6	157222509	157222659	2	55.02533	N/A	N/A	N/A
NM_025000.7.4	DCAF17	chr2	172305190	172314585	2	55.02533	N/A	N/A	N/A
NM_015172.12.8	PRRC2C	chr1	171492359	171502100	2	55.02533	N/A	N/A	N/A
NM_001286057.2.2	ADCY7	chr16	50321822	50322261	2	55.02533	N/A	N/A	N/A
NR_024573.8.7	-	chr17	40342202	40342775	2	55.02533	N/A	N/A	N/A
NM_003831.7.3	RIOK3	chr18	21043930	21047490	2	55.02533	N/A	N/A	N/A
NM_001271.26.21	CHD2	chr15	93521463	93528903	2	55.02533	N/A	N/A	N/A
NM_003502.5.3	AXIN1	chr16	354303	364683	2	55.02533	N/A	N/A	N/A
NM_001002264.9.2	EPSTI1	chr13	43491676	43544806	2	55.02533	N/A	N/A	N/A
NM_194313.6.5	KIF24	chr9	34286614	34290387	2	55.02533	N/A	N/A	N/A

NM_033000.25.23	GTF2I	chr7	74162351	74163788	2	55.02533	N/A	N/A	N/A
NM_001184856.4.2	KLHDC4	chr16	87788799	87795646	2	55.02533	N/A	N/A	N/A
NM_021644.6.2	HNRNPH3	chr10	70096955	70099312	2	55.02533	N/A	N/A	N/A
NM_030938.7.6	VMP1	chr17	57842331	57851246	2	55.02533	N/A	N/A	N/A
NR_037663.4.3	-	chr1	202126866	202127376	2	55.02533	N/A	N/A	N/A
NM_015132.5.2	SNX13	chr7	17929985	17937069	2	55.02533	N/A	N/A	N/A
NM_199420.7.6	POLQ	chr3	121248491	121252056	2	55.02533	N/A	N/A	N/A
NM_001286760.11.10	CERCAM	chr9	131196380	131196892	2	55.02533	N/A	N/A	N/A
NM_001136123.12.10	SLF2	chr10	102697158	102703885	2	55.02533	N/A	N/A	N/A
NM_004333.8.4	BRAF	chr7	140494107	140508795	2	55.02533	N/A	N/A	N/A
NM_020401.12.11	NUP107	chr12	69107509	69109520	2	55.02533	N/A	N/A	N/A
NM_001725.10.10	BPI	chr20	36954666	36954834	2	55.02533	N/A	N/A	N/A
NM_017652.2.2	ZNF586	chr19	58287910	58288037	2	55.02533	N/A	N/A	N/A
NM_001145160.6.5	TPM4	chr19	16198836	16199930	2	55.02533	N/A	N/A	N/A
NM_015172.25.23	PRRC2C	chr1	171537385	171544267	2	55.02533	N/A	N/A	N/A
NM_030647.5.3	KDM7A	chr7	139827241	139833456	2	55.02533	N/A	N/A	N/A
NM_001100399.9.5	PDS5A	chr4	39915230	39927553	2	55.02533	N/A	N/A	N/A
NM_194332.2.2	RNF38	chr9	36375930	36376124	2	55.02533	N/A	N/A	N/A
NM_001278716.7.5	FBXL4	chr6	99347143	99365595	2	55.02533	N/A	N/A	N/A
NM_015446.29.28	AHCTF1	chr1	247024200	247025455	2	55.02533	N/A	N/A	N/A
NM_001288955.5.4	TTC7A	chr2	47202111	47206046	2	55.02533	N/A	N/A	N/A
NM_001080543.2.2	CACTIN	chr19	3623685	3624160	2	55.02533	N/A	N/A	N/A
NM_001142936.5.3	DAGLB	chr7	6465618	6476164	2	55.02533	N/A	N/A	N/A
NR_037947.5.4	-	chr5	6623326	6625782	2	55.02533	N/A	N/A	N/A
NM_203447.18.16	DOCK8	chr9	370229	372286	2	55.02533	N/A	N/A	N/A
NM_015865.8.7	SLC14A1	chr18	43319127	43319627	2	55.02533	N/A	N/A	N/A
NM_001128210.5.5	SPRED2	chr2	65543867	65544017	2	55.02533	N/A	N/A	N/A
NM_022897.5.2	RANBP17	chr5	170305100	170323119	2	55.02533	N/A	N/A	N/A
NM_014671.5.2	UBE3C	chr7	156956503	156967728	2	55.02533	N/A	N/A	N/A
NM_001242837.16.15	AP2A2	chr11	1000431	1003804	2	55.02533	N/A	N/A	N/A
NM_005356.12.10	LCK	chr1	32745271	32745811	2	55.02533	N/A	N/A	N/A
NM_000081.44.41	LYST	chr1	235872390	235880078	2	55.02533	N/A	N/A	N/A
NM_183004.9.4	EIF5	chr14	103803013	103806140	2	55.02533	N/A	N/A	N/A

NM_001265612.18.17	CNOT1	chr16	58592376	58594266	2	55.02533	N/A	N/A	N/A
NM_001205206.5.3	SLC19A1	chr21	46945730	46952062	2	55.02533	N/A	N/A	N/A
NM_173797.13.6	PAPD4	chr5	78936673	78964851	2	55.02533	N/A	N/A	N/A
NM_020761.20.18	RPTOR	chr17	78865519	78867665	2	55.02533	N/A	N/A	N/A
NM_025150.13.11	TARS2	chr1	150470977	150471769	2	55.02533	N/A	N/A	N/A
NM_001093.46.45	ACACB	chr12	109696072	109696913	2	55.02533	N/A	N/A	N/A
NM_001136196.9.5	TNPO2	chr19	12825634	12826553	2	55.02533	N/A	N/A	N/A
NM_001109662.55.44	HECTD4	chr12	112638441	112664562	2	55.02533	N/A	N/A	N/A
NM_001256399.6.3	ELOVL1	chr1	43830212	43831047	2	55.02533	N/A	N/A	N/A
NM_014960.10.9	ARSG	chr17	66381204	66391334	2	55.02533	N/A	N/A	N/A
NM_003370.3.2	VASP	chr19	46020920	46021352	2	55.02533	N/A	N/A	N/A
NM_152586.5.4	USP54	chr10	75302560	75302889	2	55.02533	N/A	N/A	N/A
NM_001024210.3.2	S100A13	chr1	153603486	153604716	2	55.02533	N/A	N/A	N/A
NM_001002860.4.4	BTBD7	chr14	93730130	93730339	2	55.02533	N/A	N/A	N/A
NM_025160.7.4	WDR26	chr1	224599128	224612356	2	55.02533	N/A	N/A	N/A
NM_001080432.5.4	FTO	chr16	53878066	53907777	2	55.02533	N/A	N/A	N/A
NM_001080449.3.2	DNA2	chr10	70227879	70229920	2	55.02533	N/A	N/A	N/A
NM_001145811.7.4	SOX6	chr11	16133348	16256217	2	55.02533	N/A	N/A	N/A
NM_001145811.7.5	SOX6	chr11	16133348	16208501	2	55.02533	N/A	N/A	N/A
NM_004529.8.6	MLLT3	chr9	20360739	20365742	2	55.02533	N/A	N/A	N/A
NM_006312.13.8	NCOR2	chr12	124911167	124941708	2	55.02533	N/A	N/A	N/A
NM_001282983.7.5	FARP2	chr2	242346951	242352836	2	55.02533	N/A	N/A	N/A
NM_001417.8.5	EIF4B	chr12	53415589	53421972	2	55.02533	N/A	N/A	N/A
NM_194260.6.2	UBE2I	chr16	1364020	1370518	2	55.02533	N/A	N/A	N/A
NM_006854.4.4	KDELR2	chr7	6505701	6505954	2	55.02533	N/A	N/A	N/A
NM_007368.3.2	RASA3	chr13	114817526	114839312	1	27.51266	N/A	N/A	N/A
NM_024541.8.3	C10orf76	chr10	103783233	103793096	1	27.51266	N/A	N/A	N/A
NM_030581.13.3	WDR59	chr16	74949767	74990508	1	27.51266	N/A	N/A	N/A
NM_138572.7.6	TAF8	chr6	42034049	42036345	1	27.51266	N/A	N/A	N/A
NM_016316.4.2	REV1	chr2	100065797	100081447	1	27.51266	N/A	N/A	N/A
NM_013236.6.2	ATXN10	chr22	46085591	46114373	1	27.51266	N/A	N/A	N/A
NM_001376.69.63	DYNC1H1	chr14	102506572	102509085	1	27.51266	N/A	N/A	N/A
NM_003489.2.2	NRIP1	chr21	16415815	16415895	1	27.51266	N/A	N/A	N/A

NM_018706.6.2	DHTKD1	chr10	12123470	12133683	1	27.51266	N/A	N/A	N/A
NM_004939.13.8	DDX1	chr2	15743315	15747437	1	27.51266	N/A	N/A	N/A
NM_000188.15.13	HK1	chr10	71146078	71152064	1	27.51266	N/A	N/A	N/A
NM_005848.28.25	DENND4A	chr15	65959786	65962526	1	27.51266	N/A	N/A	N/A
NM_002074.7.4	GNB1	chr1	1735857	1749314	1	27.51266	N/A	N/A	N/A
NM_001256827.3.2	SMG6	chr17	2139785	2148013	1	27.51266	N/A	N/A	N/A
NR_052013.12.9	-	chr2	15629017	15674765	1	27.51266	N/A	N/A	N/A
NM_014826.22.21	CDC42BPA	chr1	227227831	227235711	1	27.51266	N/A	N/A	N/A
NM_006600.6.5	NUDC	chr1	27269150	27269556	1	27.51266	N/A	N/A	N/A
NM_014826.22.20	CDC42BPA	chr1	227227831	227257554	1	27.51266	N/A	N/A	N/A
NM_012455.10.3	PSD4	chr2	113942533	113951525	1	27.51266	N/A	N/A	N/A
NM_002830.13.12	PTPN4	chr2	120677644	120684242	1	27.51266	N/A	N/A	N/A
NM_004796.11.8	NRXN3	chr14	79423584	79434689	1	27.51266	N/A	N/A	N/A
NR_073136.12.11	-	chr22	26853824	26854543	1	27.51266	N/A	N/A	N/A
NR_104486.13.9	-	chr13	21955572	21965993	1	27.51266	N/A	N/A	N/A
NM_001242599.9.6	RBM39	chr20	34304661	34313077	1	27.51266	N/A	N/A	N/A
NM_001136554.12.3	TLK1	chr2	171884848	171939362	1	27.51266	N/A	N/A	N/A
NM_015578.6.5	LSM14A	chr19	34706028	34706566	1	27.51266	N/A	N/A	N/A
NM_015255.42.39	UBR2	chr6	42643793	42647570	1	27.51266	N/A	N/A	N/A
NM_006283.8.7	TACC1	chr8	38693679	38696064	1	27.51266	N/A	N/A	N/A
NM_001009993.3.2	FAM168B	chr2	131829427	131840230	1	27.51266	N/A	N/A	N/A
NM_013438.6.2	UBQLN1	chr9	86292641	86301070	1	27.51266	N/A	N/A	N/A
NM_006401.4.2	ANP32B	chr9	100756912	100767435	1	27.51266	N/A	N/A	N/A
NM_174916.46.41	UBR1	chr15	43242459	43258484	1	27.51266	N/A	N/A	N/A
NM_022153.4.2	C10orf54	chr10	73515115	73521783	1	27.51266	N/A	N/A	N/A
NM_001282873.54.48	UBR5	chr8	103276701	103285000	1	27.51266	N/A	N/A	N/A
NM_024622.11.9	FASTKD1	chr2	170394522	170401345	1	27.51266	N/A	N/A	N/A
NM_001080495.21.20	TNRC18	chr7	5363844	5364879	1	27.51266	N/A	N/A	N/A
NM_018442.4.3	DCAF6	chr1	167935866	167944253	1	27.51266	N/A	N/A	N/A
NM_015327.7.6	SMG5	chr1	156242110	156243241	1	27.51266	N/A	N/A	N/A
NM_001655.5.2	ARCN1	chr11	118451960	118455359	1	27.51266	N/A	N/A	N/A
NM_003101.9.7	SOAT1	chr1	179310162	179312078	1	27.51266	N/A	N/A	N/A
NM_001130028.12.5	CLK3	chr15	74914834	74921373	1	27.51266	N/A	N/A	N/A

NM_017508.8.7	SOX6	chr11	16119154	16133469	1	27.51266	N/A	N/A	N/A
NM_032389.15.11	ARFGAP2	chr11	47187818	47189802	1	27.51266	N/A	N/A	N/A
NM_057175.9.8	NAA15	chr4	140272344	140272765	1	27.51266	N/A	N/A	N/A
NM_005993.19.15	TBCD	chr17	80842020	80861354	1	27.51266	N/A	N/A	N/A
NM_001009905.6.2	B3GNTL1	chr17	80962990	81006661	1	27.51266	N/A	N/A	N/A
NR_073029.6.2	-	chr10	51606987	51620382	1	27.51266	N/A	N/A	N/A
NM_005548.3.2	KARS	chr16	75674081	75675621	1	27.51266	N/A	N/A	N/A
NM_001099432.23.21	BCAS3	chr17	59152280	59161925	1	27.51266	N/A	N/A	N/A
NM_001281734.6.4	ZFYVE1	chr14	73445568	73460065	1	27.51266	N/A	N/A	N/A
NM_199141.4.3	CARM1	chr19	11018714	11019883	1	27.51266	N/A	N/A	N/A
NM_020120.40.38	UGGT1	chr2	128941248	128945188	1	27.51266	N/A	N/A	N/A
NM_002792.4.2	PSMA7	chr20	60714130	60716000	1	27.51266	N/A	N/A	N/A
NM_000218.6.3	KCNQ1	chr11	2591857	2594216	1	27.51266	N/A	N/A	N/A
NM_003905.11.5	NAE1	chr16	66850483	66857503	1	27.51266	N/A	N/A	N/A
NM_018622.9.8	PARL	chr3	183551279	183551613	1	27.51266	N/A	N/A	N/A
NM_001288733.2.2	TEX2	chr17	62289933	62291602	1	27.51266	N/A	N/A	N/A
NM_004713.26.17	NEMF	chr14	50262508	50281575	1	27.51266	N/A	N/A	N/A
NM_016733.4.2	LIMK2	chr22	31654276	31656063	1	27.51266	N/A	N/A	N/A
NM_001128148.16.14	TFRC	chr3	195785154	195787118	1	27.51266	N/A	N/A	N/A
NM_001242614.4.3	CD99L2	chrX	149983334	149984551	1	27.51266	N/A	N/A	N/A
NM_001104546.13.12	RPP30	chr10	92661999	92663033	1	27.51266	N/A	N/A	N/A
NR_033265.7.5	-	chr17	80361812	80364358	1	27.51266	N/A	N/A	N/A
NM_002741.9.8	PKN1	chr19	14568842	14569187	1	27.51266	N/A	N/A	N/A
NM_153207.4.2	AEBP2	chr12	19615443	19646920	1	27.51266	N/A	N/A	N/A
NM_016823.2.2	CRK	chr17	1339913	1340449	1	27.51266	N/A	N/A	N/A
NM_133259.30.28	LRPPRC	chr2	44139570	44145537	1	27.51266	N/A	N/A	N/A
NM_001242361.9.6	NRDC	chr1	52289335	52299842	1	27.51266	N/A	N/A	N/A
NM_019009.5.2	TOLLIP	chr11	1307231	1317024	1	27.51266	N/A	N/A	N/A
NM_001429.10.6	EP300	chr22	41527391	41537226	1	27.51266	N/A	N/A	N/A
NM_183009.6.3	KIAA1429	chr8	95543190	95550574	1	27.51266	N/A	N/A	N/A
NM_001190440.39.37	NCOR1	chr17	15960827	15961913	1	27.51266	N/A	N/A	N/A
NM_024959.11.9	SLC8B1	chr12	113753139	113754806	1	27.51266	N/A	N/A	N/A
NM_139244.4.2	STXBP5	chr6	147527106	147560406	1	27.51266	N/A	N/A	N/A

NM_002501.8.3	NFIX	chr19	13183860	13192669	1	27.51266	N/A	N/A	N/A
NM_001277323.2.2	NPIPA3	chr16	14849048	14849177	1	27.51266	N/A	N/A	N/A
NM_139179.9.8	DAGLB	chr7	6461357	6464466	1	27.51266	N/A	N/A	N/A
NM_001130007.11.10	GSPT1	chr16	11976871	11979131	1	27.51266	N/A	N/A	N/A
NM_016377.5.3	AKAP7	chr6	131481198	131490413	1	27.51266	N/A	N/A	N/A
NM_000254.8.2	MTR	chr1	236966727	236979843	1	27.51266	N/A	N/A	N/A
NM_001397.10.8	ECE1	chr1	21571481	21582631	1	27.51266	N/A	N/A	N/A
NM_001242879.8.6	ELP2	chr18	33724901	33726344	1	27.51266	N/A	N/A	N/A
NM_203500.3.3	KEAP1	chr19	10602252	10602938	1	27.51266	N/A	N/A	N/A
NM_018449.13.9	UBAP2	chr9	33948371	33963789	1	27.51266	N/A	N/A	N/A
NR_104293.6.2	-	chr16	89164998	89180895	1	27.51266	N/A	N/A	N/A
NM_032482.19.17	DOT1L	chr19	2213537	2214595	1	27.51266	N/A	N/A	N/A
NM_001256.8.5	CDC27	chr17	45232037	45235669	1	27.51266	N/A	N/A	N/A
NM_005028.3.2	PIP4K2A	chr10	22896855	22898646	1	27.51266	N/A	N/A	N/A
NM_024946.6.2	FAM192A	chr16	57197912	57207781	1	27.51266	N/A	N/A	N/A
NM_024959.10.9	SLC8B1	chr12	113754330	113754806	1	27.51266	N/A	N/A	N/A
NM_002689.17.14	POLA2	chr11	65061623	65063461	1	27.51266	N/A	N/A	N/A
NM_199185.6.3	NPM1	chr5	170818308	170819982	1	27.51266	N/A	N/A	N/A
NM_001206702.8.3	SP100	chr2	231307651	231314970	1	27.51266	N/A	N/A	N/A
NM_003611.12.9	OFD1	chrX	13767545	13773361	1	27.51266	N/A	N/A	N/A
NM_001001890.4.3	RUNX1	chr21	36206706	36231875	1	27.51266	N/A	N/A	N/A
NM_001130955.4.4	ARHGEF18	chr19	7509089	7509340	1	27.51266	N/A	N/A	N/A
NM_001429.6.3	EP300	chr22	41521867	41527637	1	27.51266	N/A	N/A	N/A
NM_002745.6.4	MAPK1	chr22	22142545	22153417	1	27.51266	N/A	N/A	N/A
NR_002824.8.3	-	chr15	23330886	23356220	1	27.51266	N/A	N/A	N/A
NM_013318.2.1	PRRC2B	chr9	134305476	134308181	1	27.51266	N/A	N/A	N/A
NM_001159643.13.8	MCTP2	chr15	94899365	94928754	1	27.51266	N/A	N/A	N/A
NM_018449.12.2	UBAP2	chr9	33953282	34017187	1	27.51266	N/A	N/A	N/A
NM_013438.3.2	UBQLN1	chr9	86297865	86301070	1	27.51266	N/A	N/A	N/A
NM_032204.4.2	ASCC2	chr22	30221075	30228331	1	27.51266	N/A	N/A	N/A
NM_017964.5.3	SLC30A6	chr2	32399131	32409407	1	27.51266	N/A	N/A	N/A
NM_021813.4.2	BACH2	chr6	90916287	90981660	1	27.51266	N/A	N/A	N/A
NM_018263.7.5	ASXL2	chr2	25990451	25994409	1	27.51266	N/A	N/A	N/A

NM_001166007.11.10	EPB41	chr1	29362337	29365938	1	27.51266	N/A	N/A	N/A
NM_021639.10.7	GPBP1L1	chr1	46099160	46108171	1	27.51266	N/A	N/A	N/A
NM_022140.4.2	EPB41L4A	chr5	111611022	111643187	1	27.51266	N/A	N/A	N/A
NM_032206.2.1	NLRC5	chr16	57050985	57054919	1	27.51266	N/A	N/A	N/A
NM_001083893.16.13	STRN3	chr14	31371741	31380368	1	27.51266	N/A	N/A	N/A
NM_015016.20.17	MAST3	chr19	18246549	18252825	1	27.51266	N/A	N/A	N/A
NM_139132.14.14	NUP98	chr11	3752620	3752808	1	27.51266	N/A	N/A	N/A
NM_005154.5.5	USP8	chr15	50751196	50751359	1	27.51266	N/A	N/A	N/A
NM_002270.6.5	TNPO1	chr5	72157634	72161556	1	27.51266	N/A	N/A	N/A
NM_014946.7.4	SPAST	chr2	32323864	32341281	1	27.51266	N/A	N/A	N/A
NM_024821.5.2	CCDC134	chr22	42204878	42209449	1	27.51266	N/A	N/A	N/A
NR_003655.4.2	-	chr7	44053191	44056122	1	27.51266	N/A	N/A	N/A
NM_001202504.12.11	CTDP1	chr18	77488906	77496521	1	27.51266	N/A	N/A	N/A
NM_014756.26.21	CKAP5	chr11	46791512	46800149	1	27.51266	N/A	N/A	N/A
NM_014991.5.2	WDFY3	chr4	85771054	85853516	1	27.51266	N/A	N/A	N/A
NM_001097616.7.2	-	chr1	145884854	145919047	1	27.51266	N/A	N/A	N/A
NM_001167681.6.3	FLI1	chr11	128628009	128651918	1	27.51266	N/A	N/A	N/A
NM_007348.4.2	ATF6	chr1	161748033	161753886	1	27.51266	N/A	N/A	N/A
NM_001145819.13.8	SOX6	chr11	16036487	16119234	1	27.51266	N/A	N/A	N/A
NM_001039570.7.6	KREMEN1	chr22	29533329	29534770	1	27.51266	N/A	N/A	N/A
NM_145648.7.2	SLC15A4	chr12	129283803	129299615	1	27.51266	N/A	N/A	N/A
NM_001105562.17.15	UBE4B	chr1	10192426	10197263	1	27.51266	N/A	N/A	N/A
NR_002612.8.5	-	chr13	50571209	50618904	1	27.51266	N/A	N/A	N/A
NM_001134651.6.3	EIF4E3	chr3	71739160	71748859	1	27.51266	N/A	N/A	N/A
NM_001161346.12.11	CHFR	chr12	133428203	133430159	1	27.51266	N/A	N/A	N/A
NR_003187.4.3	-	chr7	74582341	74582682	1	27.51266	N/A	N/A	N/A
NM_018231.11.7	SLC38A7	chr16	58704016	58710250	1	27.51266	N/A	N/A	N/A
NR_023361.3.2	-	chr15	90431752	90432372	1	27.51266	N/A	N/A	N/A
NM_018107.4.2	RBM23	chr14	23375403	23380612	1	27.51266	N/A	N/A	N/A
NM_001286577.9.6	C2CD3	chr11	73829272	73844602	1	27.51266	N/A	N/A	N/A
NM_005093.6.5	CBFA2T2	chr20	32207322	32211102	1	27.51266	N/A	N/A	N/A
NM_007348.12.10	ATF6	chr1	161816238	161823113	1	27.51266	N/A	N/A	N/A
NM_001684.18.12	ATP2B4	chr1	203680004	203691817	1	27.51266	N/A	N/A	N/A

NM_002439.12.8	MSH3	chr5	79974745	80040434	1	27.51266	N/A	N/A	N/A
NM_020925.20.19	CACHD1	chr1	65139030	65141244	1	27.51266	N/A	N/A	N/A
NM_005845.19.14	ABCC4	chr13	95813442	95822882	1	27.51266	N/A	N/A	N/A
NR_003187.5.3	-	chr7	74580928	74582682	1	27.51266	N/A	N/A	N/A
NM_198433.8.6	AURKA	chr20	54956488	54959380	1	27.51266	N/A	N/A	N/A
NM_138714.15.15	NFAT5	chr16	69729038	69729282	1	27.51266	N/A	N/A	N/A
NM_005561.4.2	LAMP1	chr13	113960799	113965182	1	27.51266	N/A	N/A	N/A
NM_002398.9.7	MEIS1	chr2	66691240	66775151	1	27.51266	N/A	N/A	N/A
NM_133174.3.2	APBB3	chr5	139943178	139943500	1	27.51266	N/A	N/A	N/A
NM_024857.19.11	ATAD5	chr17	29192721	29214390	1	27.51266	N/A	N/A	N/A
NM_001784.11.10	ADGRE5	chr19	14513408	14515374	1	27.51266	N/A	N/A	N/A
NM_003627.11.9	SLC43A1	chr11	57258696	57259335	1	27.51266	N/A	N/A	N/A
NM_001261401.4.2	TSN	chr2	122514815	122519100	1	27.51266	N/A	N/A	N/A
NM_001256356.2.2	UBE2L3	chr22	21965145	21965332	1	27.51266	N/A	N/A	N/A
NM_006184.7.5	NUCB1	chr19	49414405	49416821	1	27.51266	N/A	N/A	N/A
NM_015168.9.7	ZC3H4	chr19	47587601	47588671	1	27.51266	N/A	N/A	N/A
NM_152564.29.27	VPS13B	chr8	100515063	100523740	1	27.51266	N/A	N/A	N/A
NM_001033553.14.8	SPECC1	chr17	20149238	20209395	1	27.51266	N/A	N/A	N/A
NM_000713.3.2	BLVRB	chr19	40964061	40964452	1	27.51266	N/A	N/A	N/A
NM_004371.8.6	COPA	chr1	160293220	160302347	1	27.51266	N/A	N/A	N/A
NM_022662.9.9	ANAPC1	chr2	112621251	112621472	1	27.51266	N/A	N/A	N/A
NM_152713.6.4	STT3A	chr11	125472197	125474142	1	27.51266	N/A	N/A	N/A
NM_138392.12.10	SHKBP1	chr19	41088256	41089623	1	27.51266	N/A	N/A	N/A
NM_016334.7.2	GPR89B	chr1	147408740	147426634	1	27.51266	N/A	N/A	N/A
NM_024770.5.4	METTL8	chr2	172193962	172196064	1	27.51266	N/A	N/A	N/A
NM_012318.11.7	LETM1	chr4	1821064	1827410	1	27.51266	N/A	N/A	N/A
NM_152609.2.2	CNST	chr1	246754813	246755243	1	27.51266	N/A	N/A	N/A
NM_013379.3.2	DPP7	chr9	140008694	140009028	1	27.51266	N/A	N/A	N/A
NM_001114380.9.6	ITGAL	chr16	30495147	30500707	1	27.51266	N/A	N/A	N/A
NM_147782.4.2	CTSB	chr8	11710118	11721972	1	27.51266	N/A	N/A	N/A
NM_001284401.7.6	TAMM41	chr3	11849306	11851156	1	27.51266	N/A	N/A	N/A
NM_007194.4.3	CHEK2	chr22	29120964	29121355	1	27.51266	N/A	N/A	N/A
NM_016172.7.5	UBAC1	chr9	138836873	138838217	1	27.51266	N/A	N/A	N/A

NR_048548.4.4	-	chr1	44474024	44474295	1	27.51266	N/A	N/A	N/A
NM_005415.5.4	SLC20A1	chr2	113405229	113410375	1	27.51266	N/A	N/A	N/A
NM_173822.5.4	FAM126B	chr2	201881713	201887655	1	27.51266	N/A	N/A	N/A
NM_003105.5.3	SORL1	chr11	121348826	121360819	1	27.51266	N/A	N/A	N/A
NM_001289.3.2	CLIC2	chrX	154528097	154528458	1	27.51266	N/A	N/A	N/A
NM_194460.4.2	RNF126	chr19	651610	652884	1	27.51266	N/A	N/A	N/A
NM_173822.5.3	FAM126B	chr2	201881713	201888762	1	27.51266	N/A	N/A	N/A
NM_001145440.5.5	TYW1B	chr7	72277658	72277949	1	27.51266	N/A	N/A	N/A
NM_022140.9.2	EPB41L4A	chr5	111594925	111643187	1	27.51266	N/A	N/A	N/A
NM_054027.9.3	ANKH	chr5	14716814	14758707	1	27.51266	N/A	N/A	N/A
NM_018449.6.2	UBAP2	chr9	33986757	34017187	1	27.51266	N/A	N/A	N/A
NM_005192.5.2	CDKN3	chr14	54866611	54878424	1	27.51266	N/A	N/A	N/A
NM_021994.2.2	ZNF277	chr7	111926927	111927129	1	27.51266	N/A	N/A	N/A
NM_012138.8.5	AATF	chr17	35343915	35348156	1	27.51266	N/A	N/A	N/A
NM_003234.4.3	TFRC	chr3	195800800	195802231	1	27.51266	N/A	N/A	N/A
NM_001033553.12.8	SPECC1	chr17	20149238	20163607	1	27.51266	N/A	N/A	N/A
NM_172070.10.2	UBR3	chr2	170728745	170762674	1	27.51266	N/A	N/A	N/A
NM_001080.5.2	ALDH5A1	chr6	24502750	24515538	1	27.51266	N/A	N/A	N/A
NM_001159644.9.5	MCTP2	chr15	94927250	94945248	1	27.51266	N/A	N/A	N/A
NM_016589.5.2	TIMMDC1	chr3	119219541	119232566	1	27.51266	N/A	N/A	N/A
NM_001280.6.2	CIRBP	chr19	1270926	1272050	1	27.51266	N/A	N/A	N/A
NM_015140.9.6	TTLL12	chr22	43569720	43572402	1	27.51266	N/A	N/A	N/A
NM_007051.6.5	FAF1	chr1	51204534	51210447	1	27.51266	N/A	N/A	N/A
NM_001137550.8.4	LRRFIP1	chr2	238626404	238636578	1	27.51266	N/A	N/A	N/A
NM_015358.6.2	MORC3	chr21	37705943	37713844	1	27.51266	N/A	N/A	N/A
NM_001923.10.8	DDB1	chr11	61089066	61090566	1	27.51266	N/A	N/A	N/A
NR_037659.10.7	-	chr12	89853414	89861462	1	27.51266	N/A	N/A	N/A
NM_003922.40.39	HERC1	chr15	63961736	63964893	1	27.51266	N/A	N/A	N/A
NM_003756.5.3	EIF3H	chr8	117668094	117671219	1	27.51266	N/A	N/A	N/A
NM_022918.13.9	TMEM135	chr11	87016977	87029277	1	27.51266	N/A	N/A	N/A
NR_027655.4.2	-	chr21	30693541	30702014	1	27.51266	N/A	N/A	N/A
NM_178314.4.3	RILPL1	chr12	123983090	123984083	1	27.51266	N/A	N/A	N/A
NM_020935.7.3	USP37	chr2	219411641	219425606	1	27.51266	N/A	N/A	N/A

NM_001282728.6.4	PXYLP1	chr3	140997183	141006295	1	27.51266	N/A	N/A	N/A
NM_003908.7.4	EIF2S2	chr20	32681480	32686439	1	27.51266	N/A	N/A	N/A
NM_014283.6.2	SUCO	chr1	172520651	172526934	1	27.51266	N/A	N/A	N/A
NM_001410.5.4	MEGF8	chr19	42839186	42839537	1	27.51266	N/A	N/A	N/A
NM_001282663.3.3	MICAL2	chr11	12183625	12183966	1	27.51266	N/A	N/A	N/A
NM_001080539.15.14	CCDC150	chr2	197559769	197565904	1	27.51266	N/A	N/A	N/A
NM_000208.9.3	INSR	chr19	7163042	7184648	1	27.51266	N/A	N/A	N/A
NM_016316.5.2	REV1	chr2	100058778	100081447	1	27.51266	N/A	N/A	N/A
NM_003478.16.14	CUL5	chr11	107965117	107966418	1	27.51266	N/A	N/A	N/A
NM_001284305.10.9	TBC1D22A	chr22	47370185	47393605	1	27.51266	N/A	N/A	N/A
NM_001135771.2.2	RPN2	chr20	35812582	35812776	1	27.51266	N/A	N/A	N/A
NM_005923.7.4	MAP3K5	chr6	137015277	137019820	1	27.51266	N/A	N/A	N/A
NM_001278792.6.4	RFC2	chr7	73654267	73661093	1	27.51266	N/A	N/A	N/A
NM_001115113.8.4	WDR26	chr1	224592131	224612356	1	27.51266	N/A	N/A	N/A
NM_001282224.9.7	ATP7A	chrX	77264598	77267171	1	27.51266	N/A	N/A	N/A
**NM_144604.8.7**	**ZC3H18**	**chr16**	**88675341**	**88677944**	**5**	**137.5633**	**1**	**20.925**	**-2.7168**
**NM_001282238.2.2**	**BCL11B**	**chr14**	**99723807**	**99724173**	**5**	**137.5633**	**1**	**20.925**	**-2.7168**
NM_001287816.6.5	UBAP2L	chr1	154207066	154207767	5	137.5633	1	20.925	-2.7168
NM_015431.5.2	TRIM58	chr1	248023918	248031365	5	137.5633	1	20.925	-2.7168
NM_144643.9.6	SCLT1	chr4	129913321	129925031	5	137.5633	1	20.925	-2.7168
NM_017458.5.4	MVP	chr16	29845053	29845387	9	247.614	2	41.85	-2.5648
NR_024448.3.2	-	chr22	24056373	24057381	4	110.0507	1	20.925	-2.3949
NM_006527.7.3	SLBP	chr4	1696500	1705427	4	110.0507	1	20.925	-2.3949
NM_020901.4.2	PHRF1	chr11	581491	587464	4	110.0507	1	20.925	-2.3949
NM_015015.9.6	KDM4B	chr19	5047486	5082515	4	110.0507	1	20.925	-2.3949
NM_138392.12.11	SHKBP1	chr19	41089303	41089623	15	412.6899	4	83.7	-2.3018
NM_001243403.12.10	CLEC16A	chr16	11096930	11118778	3	82.53799	1	20.925	-1.9798
NM_201277.4.3	CNN2	chr19	1032390	1032695	3	82.53799	1	20.925	-1.9798
NM_030767.14.12	AKNA	chr9	117118204	117120447	3	82.53799	1	20.925	-1.9798
NM_001042734.5.4	SEC24B	chr4	110412482	110416012	6	165.076	2	41.85	-1.9798
NM_032341.8.3	DDI2	chr1	15956819	15978390	3	82.53799	1	20.925	-1.9798
NM_139015.6.4	SPPL3	chr12	121220457	121222396	3	82.53799	1	20.925	-1.9798

NM_003298.12.11	NR2C2	chr3	15073885	15076316	3	82.53799	1	20.925	-1.9798
NM_001244713.5.2	RAD23B	chr9	110062421	110074018	3	82.53799	1	20.925	-1.9798
NM_018449.8.7	UBAP2	chr9	33971648	33973235	3	82.53799	1	20.925	-1.9798
NR_024497.7.6	-	chr10	38734344	38736649	3	82.53799	1	20.925	-1.9798
NM_015097.6.2	CLASP2	chr3	33725850	33738425	3	82.53799	1	20.925	-1.9798
NM_015161.3.2	ARL6IP1	chr16	18809246	18810156	8	220.1013	3	62.775	-1.8099
NM_003922.25.22	HERC1	chr15	63998978	64008672	2	55.02533	1	20.925	-1.3949
NM_012327.29.22	PIGN	chr18	59739905	59763183	2	55.02533	1	20.925	-1.3949
NM_001278438.13.9	FNDC3A	chr13	49741379	49749677	2	55.02533	1	20.925	-1.3949
NM_001876.9.7	CPT1A	chr11	68560782	68564401	2	55.02533	1	20.925	-1.3949
NM_022458.4.2	LMBR1	chr7	156619298	156629579	2	55.02533	1	20.925	-1.3949
NM_001202543.21.21	CUX1	chr7	101870646	101870949	4	110.0507	2	41.85	-1.3949
NM_000265.5.3	NCF1	chr7	74193427	74195181	2	55.02533	1	20.925	-1.3949
NM_007111.4.3	TFDP1	chr13	114265310	114277601	8	220.1013	4	83.7	-1.3949
NM_001109662.52.45	HECTD4	chr12	112645677	112657293	2	55.02533	1	20.925	-1.3949
NM_002027.3.2	FNTA	chr8	42914234	42919358	2	55.02533	1	20.925	-1.3949
NR_001591.6.5	-	chr22	17117929	17119630	2	55.02533	1	20.925	-1.3949
NM_001164315.22.16	ANKRD36	chr2	97823843	97833504	2	55.02533	1	20.925	-1.3949
NR_037615.4.2	-	chr12	70671911	70704797	2	55.02533	1	20.925	-1.3949
NM_019034.4.3	RHOF	chr12	122218777	122219098	4	110.0507	2	41.85	-1.3949
NM_001080398.33.30	KIAA0368	chr9	114148656	114154104	4	110.0507	2	41.85	-1.3949
NM_015016.26.26	MAST3	chr19	18258252	18258434	2	55.02533	1	20.925	-1.3949
NM_000578.8.4	SLC11A1	chr2	219249869	219252678	2	55.02533	1	20.925	-1.3949
NM_199203.5.3	TMEM189-UBE2V1	chr20	48744511	48747484	2	55.02533	1	20.925	-1.3949
NM_025219.4.2	DNAJC5	chr20	62559687	62562375	6	165.076	3	62.775	-1.3949
NM_020478.4.3	ANK1	chr8	41518947	41519459	5	137.5633	3	62.775	-1.1318
NM_006306.15.14	SMC1A	chrX	53430497	53430825	5	137.5633	3	62.775	-1.1318
NM_024947.6.5	PHC3	chr3	169863210	169867032	19	522.7406	12	251.1	-1.0578
NM_002945.4.2	RPA1	chr17	1746096	1747980	3	82.53799	2	41.85	-0.9798
NM_001247996.5.4	ASAP1	chr8	131249167	131370389	3	82.53799	2	41.85	-0.9798
NM_174937.3.2	TCERG1L	chr10	133106473	133107562	3	82.53799	2	41.85	-0.9798
NM_031417.10.9	MARK4	chr19	45781180	45781873	3	82.53799	2	41.85	-0.9798
NM_007345.9.2	ZNF236	chr18	74561481	74593468	3	82.53799	2	41.85	-0.9798

NM_014681.6.6	DHX34	chr19	47865732	47865950	3	82.53799	2	41.85	-0.9798
NM_021639.8.7	GPBP1L1	chr1	46105881	46108171	7	192.5886	5	104.62	-0.8803
NM_001280.6.4	CIRBP	chr19	1271327	1272050	7	192.5886	5	104.62	-0.8803
NM_001417.8.7	EIF4B	chr12	53421565	53421972	4	110.0507	3	62.775	-0.8099
NM_012181.6.5	FKBP8	chr19	18648410	18649246	33	907.9179	25	523.12	-0.7954
NM_024757.25.19	EHMT1	chr9	140705912	140712590	5	137.5633	4	83.7	-0.7168
NM_018482.13.8	ASAP1	chr8	131164981	131193126	17	467.7153	15	313.87	-0.5754
NM_014615.2.2	GSE1	chr16	85667519	85667738	143	3934.311	134	2803.9	-0.4887
NM_198925.7.2	SEMA4B	chr15	90760670	90764997	6	165.076	6	125.55	-0.3949
NM_001012614.5.2	CTBP1	chr4	1219147	1235307	3	82.53799	3	62.775	-0.3949
NM_001134937.12.10	FIP1L1	chr4	54280781	54294350	3	82.53799	3	62.775	-0.3949
NM_018463.9.5	ITFG2	chr12	2929251	2930958	3	82.53799	3	62.775	-0.3949
NM_012203.4.2	GRHPR	chr9	37424841	37426651	5	137.5633	5	104.62	-0.3949
NM_015622.14.13	CCZ1	chr7	5963017	5963593	1	27.51266	1	20.925	-0.3949
NM_004964.6.5	HDAC1	chr1	32792539	32793278	1	27.51266	1	20.925	-0.3949
NM_018449.13.13	UBAP2	chr9	33948371	33948585	2	55.02533	2	41.85	-0.3949
NM_001145819.9.5	SOX6	chr11	16117541	16208501	1	27.51266	1	20.925	-0.3949
NM_174902.5.5	LDLRAD3	chr11	36248634	36248980	1	27.51266	1	20.925	-0.3949
NM_001080475.3.3	PLEKHM3	chr2	208841374	208842310	1	27.51266	1	20.925	-0.3949
NM_053043.7.2	RBM33	chr7	155457868	155499762	1	27.51266	1	20.925	-0.3949
NM_001247996.6.4	ASAP1	chr8	131226801	131370389	7	192.5886	7	146.47	-0.3949
NM_006750.5.5	SNTB2	chr16	69317950	69318147	1	27.51266	1	20.925	-0.3949
NM_016026.6.5	RDH11	chr14	68151731	68157138	1	27.51266	1	20.925	-0.3949
NM_012387.8.7	PADI4	chr1	17668437	17668897	1	27.51266	1	20.925	-0.3949
NM_005124.5.3	NUP153	chr6	17675135	17676001	1	27.51266	1	20.925	-0.3949
NM_001008738.12.7	FNIP1	chr5	131013395	131044965	1	27.51266	1	20.925	-0.3949
NM_001039670.7.6	IFFO1	chr12	6657590	6657991	1	27.51266	1	20.925	-0.3949
NM_004339.5.3	PTTG1IP	chr21	46275124	46281186	2	55.02533	2	41.85	-0.3949
NM_001282752.6.2	PUM2	chr2	20507738	20527139	1	27.51266	1	20.925	-0.3949
NM_001130825.3.3	SUPT5H	chr19	39943995	39944161	1	27.51266	1	20.925	-0.3949
NM_032773.17.16	LRCH3	chr3	197592293	197593090	1	27.51266	1	20.925	-0.3949
NM_006996.2.2	SLC19A2	chr1	169446392	169446995	1	27.51266	1	20.925	-0.3949
NM_173834.6.2	YIPF6	chrX	67731690	67742759	2	55.02533	2	41.85	-0.3949

NM_017698.3.2	TMEM164	chrX	109310574	109352374	1	27.51266	1	20.925	-0.3949
NM_198097.14.13	CCZ1B	chr7	6840578	6841154	1	27.51266	1	20.925	-0.3949
NR_002824.4.4	-	chr15	23338852	23339028	1	27.51266	1	20.925	-0.3949
NM_001128148.18.14	TFRC	chr3	195780288	195787118	1	27.51266	1	20.925	-0.3949
NM_004741.5.2	NOLC1	chr10	103916775	103917971	1	27.51266	1	20.925	-0.3949
NM_001257386.10.8	ABCG2	chr4	89028335	89036210	1	27.51266	1	20.925	-0.3949
NM_001083536.3.2	FGD3	chr9	95737519	95738991	2	55.02533	2	41.85	-0.3949
NM_001163636.22.19	GTF2I	chr7	74157787	74160750	1	27.51266	1	20.925	-0.3949
NM_015148.3.3	PASK	chr2	242079935	242080168	1	27.51266	1	20.925	-0.3949
NM_003922.2.2	HERC1	chr15	64066892	64067848	1	27.51266	1	20.925	-0.3949
NM_024821.4.2	CCDC134	chr22	42204878	42206295	2	55.02533	2	41.85	-0.3949
NM_007202.4.4	AKAP10	chr17	19861326	19861884	1	27.51266	1	20.925	-0.3949
NM_000051.37.35	ATM	chr11	108172374	108175579	1	27.51266	1	20.925	-0.3949
NM_001282560.5.4	EYA3	chr1	28365349	28369161	1	27.51266	1	20.925	-0.3949
NM_012179.2.2	FBXO7	chr22	32874967	32875262	2	55.02533	2	41.85	-0.3949
NM_001191022.8.7	CTCF	chr16	67662272	67663436	1	27.51266	1	20.925	-0.3949
NM_004753.4.3	DHRS3	chr1	12638745	12639440	1	27.51266	1	20.925	-0.3949
NM_053043.9.7	RBM33	chr7	155499553	155511137	1	27.51266	1	20.925	-0.3949
NM_145912.2.2	NFAM1	chr22	42807412	42807742	1	27.51266	1	20.925	-0.3949
NM_001109662.3.2	HECTD4	chr12	112752260	112757546	1	27.51266	1	20.925	-0.3949
NM_138799.4.2	MBOAT2	chr2	9048750	9098771	10	275.1266	11	230.17	-0.2574
NM_007166.19.14	PICALM	chr11	85685750	85695016	5	137.5633	6	125.55	-0.1318
NM_001145808.14.13	ITGAM	chr16	31308834	31309275	15	412.6899	19	397.57	-0.0538
NM_000265.7.6	NCF1	chr7	74197281	74197975	3	82.53799	4	83.7	0.0202
NM_006662.27.26	SRCAP	chr16	30740286	30740893	3	82.53799	4	83.7	0.0202
NM_001286646.1.1	SLC45A4	chr8	142264087	142264728	3	82.53799	4	83.7	0.0202
NM_004380.2.2	CREBBP	chr16	3900297	3901010	6	165.076	8	167.4	0.0202
NM_016464.4.3	TMEM138	chr11	61133516	61135470	10	275.1266	14	292.95	0.0906
NM_014313.6.4	TMEM50A	chr1	25678116	25683344	2	55.02533	3	62.775	0.1901
NM_138957.3.2	MAPK1	chr22	22160138	22162135	2	55.02533	3	62.775	0.1901
NM_001282529.9.8	UBAP2	chr9	33948371	33953472	2	55.02533	3	62.775	0.1901
NM_015603.7.6	CCDC9	chr19	47767859	47768203	2	55.02533	3	62.775	0.1901
NM_017590.13.12	ZC3H7B	chr22	41738532	41739580	2	55.02533	3	62.775	0.1901

NM_002372.3.2	MAN2A1	chr5	109049220	109051965	2	55.02533	3	62.775	0.1901
NM_031490.14.13	LONP2	chr16	48381417	48382201	2	55.02533	3	62.775	0.1901
NM_138799.3.2	MBOAT2	chr2	9083315	9098771	8	220.1013	12	251.1	0.1901
NM_138576.2.2	BCL11B	chr14	99723807	99724176	5	137.5633	8	167.4	0.2832
NR_003186.4.4	-	chr7	72643600	72644294	3	82.53799	6	125.55	0.6051
NM_018107.3.2	RBM23	chr14	23378691	23380612	3	82.53799	6	125.55	0.6051
NM_005898.12.8	CAPRIN1	chr11	34104371	34111003	1	27.51266	2	41.85	0.6051
NM_020193.14.10	EMSY	chr11	76224429	76239510	1	27.51266	2	41.85	0.6051
NM_003434.6.2	ZNF133	chr20	18278628	18287037	1	27.51266	2	41.85	0.6051
NM_015622.8.5	CCZ1	chr7	5941287	5949747	1	27.51266	2	41.85	0.6051
NM_007198.4.2	PROSC	chr8	37623043	37623873	2	55.02533	4	83.7	0.6051
NM_001040153.5.2	SLAIN1	chr13	78293666	78327493	1	27.51266	2	41.85	0.6051
NM_001145819.9.8	SOX6	chr11	16117541	16119234	2	55.02533	4	83.7	0.6051
NM_015164.7.4	PLEKHM2	chr1	16044387	16047883	1	27.51266	2	41.85	0.6051
NM_001136053.3.2	TPRA1	chr3	127298582	127299006	1	27.51266	2	41.85	0.6051
NM_016955.9.2	SEPSECS	chr4	25128885	25160729	1	27.51266	2	41.85	0.6051
NM_015092.41.39	SMG1	chr16	18852886	18856973	1	27.51266	2	41.85	0.6051
NM_004514.4.2	FOXK2	chr17	80521229	80529746	1	27.51266	2	41.85	0.6051
NM_001008566.3.3	TPST2	chr22	26936754	26937684	1	27.51266	2	41.85	0.6051
NM_130807.3.3	MOB3A	chr19	2078138	2078678	1	27.51266	2	41.85	0.6051
NM_020677.4.3	NMRAL1	chr16	4516153	4519466	1	27.51266	2	41.85	0.6051
NM_001282861.15.14	GON4L	chr1	155747412	155753880	1	27.51266	2	41.85	0.6051
NM_212535.5.4	PRKCB	chr16	24043456	24046868	1	27.51266	2	41.85	0.6051
NM_001114380.15.14	ITGAL	chr16	30510394	30510810	1	27.51266	2	41.85	0.6051
NM_053002.4.2	MED12L	chr3	150834124	150845771	1	27.51266	2	41.85	0.6051
NM_001271.31.29	CHD2	chr15	93540186	93541851	1	27.51266	2	41.85	0.6051
NM_005534.6.2	IFNGR2	chr21	34787194	34805178	1	27.51266	2	41.85	0.6051
NM_001085400.6.4	RELL1	chr4	37633006	37640126	4	110.0507	8	167.4	0.6051
NM_033326.9.7	SOX6	chr11	16117541	16133469	1	27.51266	2	41.85	0.6051
NM_001097615.6.5	POLR2J3	chr7	102181539	102182109	1	27.51266	2	41.85	0.6051
NM_018237.8.8	CCAR1	chr10	70507130	70507323	1	27.51266	2	41.85	0.6051
NM_000110.8.6	DPYD	chr1	98144650	98165103	1	27.51266	2	41.85	0.6051
NM_004461.7.5	FARSA	chr19	13039155	13039661	8	220.1013	17	355.72	0.6926

NM_001167608.5.4	RHBDD1	chr2	227729319	227732034	4	110.0507	9	188.32	0.7751
NM_014661.4.4	FAM53B	chr10	126370175	126370948	4	110.0507	9	188.32	0.7751
NM_181838.6.3	UBE2D2	chr5	138979956	138994551	2	55.02533	5	104.62	0.9271
NM_001105214.9.6	ASH2L	chr8	37971709	37976881	2	55.02533	5	104.62	0.9271
NM_005908.5.2	MANBA	chr4	103635594	103647840	2	55.02533	5	104.62	0.9271
NM_003120.4.2	SPI1	chr11	47380394	47397280	2	55.02533	5	104.62	0.9271
NM_014059.4.3	RGCC	chr13	42040958	42042974	2	55.02533	5	104.62	0.9271
NM_018449.13.11	UBAP2	chr9	33948371	33956144	3	82.53799	8	167.4	1.0202
NM_152487.5.2	TMEM56	chr1	95609446	95616975	37	1017.969	101	2113.4	1.0539
NM_001005404.2.2	YPEL2	chr17	57430575	57430887	1	27.51266	3	62.775	1.1901
NM_001286249.6.2	HAGH	chr16	1866893	1873038	1	27.51266	3	62.775	1.1901
NM_018449.12.11	UBAP2	chr9	33953282	33956144	1	27.51266	3	62.775	1.1901
NM_152924.3.2	ABHD2	chr15	89656955	89659752	2	55.02533	6	125.55	1.1901
NM_181701.5.2	QSOX2	chr9	139115608	139118720	1	27.51266	3	62.775	1.1901
NM_012181.4.3	FKBP8	chr19	18650180	18650530	1	27.51266	3	62.775	1.1901
NM_001159643.1.1	MCTP2	chr15	94841429	94841959	1	27.51266	3	62.775	1.1901
NM_005848.11.6	DENND4A	chr15	66021409	66031213	2	55.02533	6	125.55	1.1901
NR_026566.8.6	-	chr10	48941193	48943556	1	27.51266	3	62.775	1.1901
NM_006836.31.29	GCN1	chr12	120592773	120593523	2	55.02533	6	125.55	1.1901
NM_015447.3.2	CAMSAP1	chr9	138773478	138774924	3	82.53799	9	188.32	1.1901
NM_022039.5.2	FBXW4	chr10	103427642	103436193	1	27.51266	3	62.775	1.1901
NM_024947.7.5	PHC3	chr3	169854206	169867032	4	110.0507	14	292.95	1.4125
NM_003439.3.2	ZKSCAN1	chr7	99621041	99621930	3	82.53799	11	230.17	1.4796
NM_014899.7.6	RHOBTB3	chr5	95091099	95099324	1	27.51266	4	83.7	1.6051
NM_080840.5.4	PTPRA	chr20	2944917	2945848	2	55.02533	8	167.4	1.6051
NR_073062.3.2	-	chr10	74474868	74475660	1	27.51266	4	83.7	1.6051
NM_006135.7.4	CAPZA1	chr1	113196219	113202401	1	27.51266	4	83.7	1.6051
NM_005174.8.3	ATP5C1	chr10	7839009	7844817	1	27.51266	4	83.7	1.6051
NM_012335.19.16	MYO1F	chr19	8601135	8604912	1	27.51266	5	104.62	1.9271
NM_005028.4.2	PIP4K2A	chr10	22880557	22898646	1	27.51266	5	104.62	1.9271
NM_013396.3.2	USP25	chr21	17135209	17138460	1	27.51266	5	104.62	1.9271
**NM_001429.9.3**	**EP300**	**chr22**	**41521867**	**41536261**	**1**	**27.51266**	**5**	**104.62**	**1.9271**
**NM_019852.3.2**	**METTL3**	**chr14**	**21971315**	**21972024**	**1**	**27.51266**	**6**	**125.55**	**2.1901**

**NM_020774.6.2**	**MIB1**	**chr18**	**19345732**	**19359646**	**1**	**27.51266**	**9**	**188.32**	**2.7751**
NM_004145.2.2	*MYO9B*	*chr19*	*17212469*	*17213367*	*1*	*27.5127*	*10*	*209.25*	*2.9271*
NM_001135610.6.2	PRDM2	chr1	14042035	14075982	N/A	N/A	1	20.925	N/A
NM_138492.5.2	PRELID2	chr5	145197456	145205763	N/A	N/A	1	20.925	N/A
NM_001195071.3.2	TMEM184B	chr22	38641940	38644025	N/A	N/A	1	20.925	N/A
NM_203364.15.12	CAPRIN1	chr11	34110941	34113603	N/A	N/A	1	20.925	N/A
NR_102264.4.3	-	chr10	70227879	70229920	N/A	N/A	1	20.925	N/A
NM_001286723.3.2	FAM120A	chr9	96233422	96238620	N/A	N/A	1	20.925	N/A
NM_153638.6.2	PANK2	chr20	3888572	3899443	N/A	N/A	1	20.925	N/A
NM_020824.7.5	ARHGAP21	chr10	24918924	24924032	N/A	N/A	1	20.925	N/A
NM_001046.15.8	SLC12A2	chr5	127474288	127488497	N/A	N/A	1	20.925	N/A
NR_037918.3.2	-	chr12	11187380	11199787	N/A	N/A	1	20.925	N/A
NM_017994.3.2	TMEM248	chr7	66406834	66410248	N/A	N/A	1	20.925	N/A
NM_001130679.5.3	EIF4E	chr4	99808229	99812483	N/A	N/A	1	20.925	N/A
NM_145177.5.4	DHRSX	chrY	2134780	2159644	N/A	N/A	1	20.925	N/A
NM_020133.2.2	AGPAT4	chr6	161653067	161653334	N/A	N/A	1	20.925	N/A
NM_014415.7.2	ZBTB11	chr3	101374947	101391057	N/A	N/A	1	20.925	N/A
NM_001005362.11.9	DNM2	chr19	10906047	10909248	N/A	N/A	1	20.925	N/A
NM_007357.9.4	COG2	chr1	230798886	230810870	N/A	N/A	1	20.925	N/A
NM_015226.21.19	CLEC16A	chr16	11214471	11220003	N/A	N/A	1	20.925	N/A
NM_015156.10.8	RCOR1	chr14	103180759	103187714	N/A	N/A	1	20.925	N/A
NM_012387.3.2	PADI4	chr1	17657463	17660504	N/A	N/A	1	20.925	N/A
NM_001195102.8.5	GSR	chr8	30546677	30560757	N/A	N/A	1	20.925	N/A
NM_001247996.8.4	ASAP1	chr8	131199481	131370389	N/A	N/A	1	20.925	N/A
NM_001163034.20.18	RPTOR	chr17	78896523	78899280	N/A	N/A	1	20.925	N/A
NM_013449.18.14	BAZ2A	chr12	56997142	56999111	N/A	N/A	1	20.925	N/A
NM_001085399.6.5	RELL1	chr4	37633006	37636745	N/A	N/A	1	20.925	N/A
NM_015555.5.4	ZNF451	chr6	56989531	56993638	N/A	N/A	1	20.925	N/A
NM_145759.3.2	TRAF5	chr1	211526580	211527809	N/A	N/A	1	20.925	N/A
NM_001199777.8.2	POC1B	chr12	89860546	89891119	N/A	N/A	1	20.925	N/A
NM_001281512.4.2	HADHB	chr2	26477114	26486347	N/A	N/A	1	20.925	N/A
NM_001134364.8.6	MAP4	chr3	47956306	47960331	N/A	N/A	1	20.925	N/A

NM_016426.9.7	GTSE1	chr22	46711928	46722551	N/A	N/A	1	20.925	N/A
NM_001122731.15.1	MICAL3	chr22	18368643	18389652	N/A	N/A	1	20.925	N/A
NM_024541.15.2	C10orf76	chr10	103761727	103799882	N/A	N/A	1	20.925	N/A
NM_001126103.18.15	RACGAP1	chr12	50384466	50386438	N/A	N/A	1	20.925	N/A
NM_001278945.5.3	PBK	chr8	27679835	27685715	N/A	N/A	1	20.925	N/A
NM_002662.16.14	PLD1	chr3	171404474	171406666	N/A	N/A	1	20.925	N/A
NM_001256045.3.2	PIK3CB	chr3	138413627	138417937	N/A	N/A	1	20.925	N/A
NM_012257.6.2	HBP1	chr7	106820323	106827126	N/A	N/A	1	20.925	N/A
NM_152641.20.16	ARID2	chr12	46254583	46287504	N/A	N/A	1	20.925	N/A
NM_175624.3.2	RAB3IP	chr12	70149163	70150443	N/A	N/A	1	20.925	N/A
NM_001109662.41.38	HECTD4	chr12	112667501	112670889	N/A	N/A	1	20.925	N/A
NM_203447.14.12	DOCK8	chr9	336581	340321	N/A	N/A	1	20.925	N/A
NM_031966.7.6	CCNB1	chr5	68470703	68471364	N/A	N/A	1	20.925	N/A
NM_018309.10.4	TBC1D23	chr3	100002450	100018175	N/A	N/A	1	20.925	N/A
NM_005921.6.2	MAP3K1	chr5	56152426	56161804	N/A	N/A	1	20.925	N/A
NM_018426.22.20	TMEM63B	chr6	44120329	44121925	N/A	N/A	1	20.925	N/A
NM_015313.36.31	ARHGEF12	chr11	120343758	120348235	N/A	N/A	1	20.925	N/A
NM_001289396.27.25	SMARCA2	chr9	2115821	2123937	N/A	N/A	1	20.925	N/A
NM_000032.10.7	ALAS2	chrX	55039918	55044098	N/A	N/A	1	20.925	N/A
NM_001191028.8.5	TNPO3	chr7	128637442	128645213	N/A	N/A	1	20.925	N/A
NM_003101.9.5	SOAT1	chr1	179306971	179312078	N/A	N/A	1	20.925	N/A
NM_002631.5.4	PGD	chr1	10463127	10464336	N/A	N/A	1	20.925	N/A
NM_005720.7.5	ARPC1B	chr7	98987527	98988876	N/A	N/A	1	20.925	N/A
NM_005720.7.6	ARPC1B	chr7	98988515	98988876	N/A	N/A	1	20.925	N/A
NM_001278387.4.2	SLC38A1	chr12	46633461	46648719	N/A	N/A	1	20.925	N/A
NM_181335.7.3	ARHGAP8	chr22	45197956	45221473	N/A	N/A	1	20.925	N/A
NM_014889.10.8	PITRM1	chr10	3201105	3202522	N/A	N/A	1	20.925	N/A
NM_152420.3.2	CARNMT1	chr9	77631183	77632364	N/A	N/A	1	20.925	N/A
NM_023034.2.2	WHSC1L1	chr8	38205014	38205733	N/A	N/A	1	20.925	N/A
NM_005338.9.6	HIP1	chr7	75197502	75211467	N/A	N/A	1	20.925	N/A
NM_005601.2.2	NKG7	chr19	51875414	51875561	N/A	N/A	1	20.925	N/A
NM_016287.8.2	HP1BP3	chr1	21091869	21107033	N/A	N/A	1	20.925	N/A
NM_015938.9.6	NMD3	chr3	160952514	160956614	N/A	N/A	1	20.925	N/A

NM_001257158.9.4	CEP41	chr7	130040547	130052081	N/A	N/A	1	20.925	N/A
NM_001270940.15.13	XPO6	chr16	28132990	28143724	N/A	N/A	1	20.925	N/A
NM_002880.7.6	RAF1	chr3	12645634	12647798	N/A	N/A	1	20.925	N/A
NM_005848.7.2	DENND4A	chr15	66030044	66053776	N/A	N/A	1	20.925	N/A
NM_021813.5.2	BACH2	chr6	90798679	90981660	N/A	N/A	1	20.925	N/A
NM_000263.5.3	NAGLU	chr17	40690356	40693224	N/A	N/A	1	20.925	N/A
NM_016284.17.17	CNOT1	chr16	58594115	58594266	N/A	N/A	1	20.925	N/A
NM_001135187.5.2	AGFG1	chr2	228356262	228389631	N/A	N/A	1	20.925	N/A
NM_015024.8.3	XPO7	chr8	21826993	21835354	N/A	N/A	1	20.925	N/A
NM_021627.10.3	SENP2	chr3	185316199	185331196	N/A	N/A	1	20.925	N/A
NM_001271287.6.3	ZNF512	chr2	27822449	27824309	N/A	N/A	1	20.925	N/A
NM_001278531.9.3	ERMARD	chr6	170155378	170162627	N/A	N/A	1	20.925	N/A
NM_005848.7.6	DENND4A	chr15	66030044	66031213	N/A	N/A	1	20.925	N/A
NM_018263.8.5	ASXL2	chr2	25982350	25994409	N/A	N/A	1	20.925	N/A
NM_014708.30.21	KNTC1	chr12	123052807	123061576	N/A	N/A	1	20.925	N/A
NM_014661.2.2	FAM53B	chr10	126395204	126395456	N/A	N/A	1	20.925	N/A
NM_014109.24.21	ATAD2	chr8	124346117	124350061	N/A	N/A	1	20.925	N/A
NM_001243403.17.11	CLEC16A	chr16	11114049	11145498	N/A	N/A	1	20.925	N/A
NR_024054.4.2	-	chr5	69493081	69517842	N/A	N/A	1	20.925	N/A
NM_153051.9.2	MTMR3	chr22	30353024	30398982	N/A	N/A	1	20.925	N/A
NM_014109.24.20	ATAD2	chr8	124346117	124351686	N/A	N/A	1	20.925	N/A
NM_018993.4.3	RIN2	chr20	19937258	19941455	N/A	N/A	1	20.925	N/A
NM_024294.3.2	C6orf106	chr6	34614377	34622556	N/A	N/A	1	20.925	N/A
NM_001278194.3.2	GANAB	chr11	62406454	62407203	N/A	N/A	1	20.925	N/A
NM_004252.3.2	SLC9A3R1	chr17	72758150	72759659	N/A	N/A	1	20.925	N/A
NM_181783.12.5	TMTC3	chr12	88553890	88584399	N/A	N/A	1	20.925	N/A
NM_003234.16.7	TFRC	chr3	195785154	195796439	N/A	N/A	1	20.925	N/A
NM_032310.3.2	CARD19	chr9	95869955	95873003	N/A	N/A	1	20.925	N/A
NM_001287822.6.4	ZWILCH	chr15	66811216	66813543	N/A	N/A	1	20.925	N/A
NM_015155.3.2	LARP4B	chr10	930386	931700	N/A	N/A	1	20.925	N/A
NM_001253912.17.15	TBC1D1	chr4	38091552	38104778	N/A	N/A	1	20.925	N/A
NM_152419.16.11	HGSNAT	chr8	43037287	43052885	N/A	N/A	1	20.925	N/A
NM_014997.5.4	KLHDC10	chr7	129760588	129762042	N/A	N/A	1	20.925	N/A

NM_014698.17.16	TMEM63A	chr1	226044352	226044717	N/A	N/A	1	20.925	N/A
NM_004798.5.3	KIF3B	chr20	30904020	30904677	N/A	N/A	1	20.925	N/A
NM_001286157.11.8	DYNC1LI2	chr16	66759730	66763002	N/A	N/A	1	20.925	N/A
NM_001285448.4.3	PDXDC1	chr16	15091609	15092262	N/A	N/A	1	20.925	N/A
NM_001097613.11.9	GPR89A	chr1	145787771	145791170	N/A	N/A	1	20.925	N/A
NM_033655.9.5	CNTNAP3	chr9	39165929	39178357	N/A	N/A	1	20.925	N/A
NM_012213.4.2	MLYCD	chr16	83940591	83945972	N/A	N/A	1	20.925	N/A
NM_003286.15.14	TOP1	chr20	39741421	39742795	N/A	N/A	1	20.925	N/A
NM_018957.10.8	SH3BP1	chr22	38040643	38041517	N/A	N/A	1	20.925	N/A
NM_175709.4.3	CBX7	chr22	39534640	39537441	N/A	N/A	1	20.925	N/A
NM_012290.8.3	TLK1	chr2	171910270	171939362	N/A	N/A	1	20.925	N/A
NM_006306.9.7	SMC1A	chrX	53435992	53438851	N/A	N/A	1	20.925	N/A
NM_001256.8.2	CDC27	chr17	45232037	45259003	N/A	N/A	1	20.925	N/A
NM_001005361.18.15	DNM2	chr19	10922939	10935897	N/A	N/A	1	20.925	N/A
NM_001286715.22.17	AGTPBP1	chr9	88200377	88211364	N/A	N/A	1	20.925	N/A
NM_004544.9.6	NDUFA10	chr2	240929490	240951113	N/A	N/A	1	20.925	N/A
NM_014709.16.11	USP34	chr2	61570949	61577828	N/A	N/A	1	20.925	N/A
NM_004360.10.3	CDH1	chr16	68835572	68849662	N/A	N/A	1	20.925	N/A
NR_033814.9.4	-	chr14	75355797	75361112	N/A	N/A	1	20.925	N/A
NM_013233.10.2	STK39	chr2	168986050	169038600	N/A	N/A	1	20.925	N/A
NM_015889.8.5	MED15	chr22	20909222	20922918	N/A	N/A	1	20.925	N/A
NM_032305.3.2	POLR3GL	chr1	145459651	145460263	N/A	N/A	1	20.925	N/A
NR_103836.7.4	-	chr6	99323290	99353546	N/A	N/A	1	20.925	N/A
NM_003223.4.2	TFAP4	chr16	4311779	4312702	N/A	N/A	1	20.925	N/A
NM_032782.5.3	HAVCR2	chr5	156522316	156531760	N/A	N/A	1	20.925	N/A
NM_014810.6.2	CEP350	chr1	179955303	179966310	N/A	N/A	1	20.925	N/A
NM_001017373.9.6	SAMD3	chr6	130475969	130505768	N/A	N/A	1	20.925	N/A
NM_032933.14.7	TMEM241	chr18	20889643	20953727	N/A	N/A	1	20.925	N/A
NM_001048166.16.14	STIL	chr1	47725960	47735538	N/A	N/A	1	20.925	N/A
NM_014868.6.2	RNF10	chr12	120984207	120995485	N/A	N/A	1	20.925	N/A
NM_001127391.3.2	ALS2CR12	chr2	202215433	202216174	N/A	N/A	1	20.925	N/A
NM_080650.8.4	DPH6	chr15	35665721	35747021	N/A	N/A	1	20.925	N/A
NM_014868.6.5	RNF10	chr12	120995084	120995485	N/A	N/A	1	20.925	N/A

NM_139179.7.3	DAGLB	chr7	6465618	6476164	N/A	N/A	1	20.925	N/A
NM_015239.18.12	AGTPBP1	chr9	88211276	88261333	N/A	N/A	1	20.925	N/A
NM_017746.11.6	TEX10	chr9	103082546	103092451	N/A	N/A	1	20.925	N/A
NM_145037.6.5	NXPE3	chr3	101520078	101525970	N/A	N/A	1	20.925	N/A
NM_001083893.10.8	STRN3	chr14	31382729	31398517	N/A	N/A	1	20.925	N/A
NM_001178056.7.4	PARP8	chr5	50055476	50059076	N/A	N/A	1	20.925	N/A
NM_015282.2.2	CLASP1	chr2	122363276	122363756	N/A	N/A	1	20.925	N/A
NM_005575.8.5	LNPEP	chr5	96328718	96333849	N/A	N/A	1	20.925	N/A
NM_003035.12.11	STIL	chr1	47745912	47748131	N/A	N/A	1	20.925	N/A
NM_139015.6.2	SPPL3	chr12	121220457	121248689	N/A	N/A	1	20.925	N/A
NM_005908.4.2	MANBA	chr4	103644027	103647840	N/A	N/A	1	20.925	N/A
NM_139015.6.3	SPPL3	chr12	121220457	121229360	N/A	N/A	1	20.925	N/A
NM_016824.13.10	ADD3	chr10	111883774	111890244	N/A	N/A	1	20.925	N/A
NM_012199.11.9	AGO1	chr1	36367074	36367938	N/A	N/A	1	20.925	N/A
NM_001282925.9.3	ABI2	chr2	204244930	204267457	N/A	N/A	1	20.925	N/A
NM_001109662.45.42	HECTD4	chr12	112657189	112666615	N/A	N/A	1	20.925	N/A
NM_025219.3.2	DNAJC5	chr20	62559687	62560878	N/A	N/A	1	20.925	N/A
NM_001169107.5.3	FAM21C	chr10	46224309	46235694	N/A	N/A	1	20.925	N/A
NM_018449.10.7	UBAP2	chr9	33960823	33973235	N/A	N/A	1	20.925	N/A
NM_020978.9.2	AMY2B	chr1	104108056	104118162	N/A	N/A	1	20.925	N/A
NM_006336.8.2	ZER1	chr9	131512894	131517938	N/A	N/A	1	20.925	N/A
NM_001987.5.5	ETV6	chr12	12022357	12022903	N/A	N/A	1	20.925	N/A
NM_001261826.3.2	AP3D1	chr19	2137725	2138713	N/A	N/A	1	20.925	N/A
NM_014976.27.26	PDCD11	chr10	105197771	105198565	N/A	N/A	1	20.925	N/A
NM_032581.10.2	FAM126A	chr7	22999874	23030758	N/A	N/A	1	20.925	N/A
NM_020820.10.6	PREX1	chr20	47305194	47324959	N/A	N/A	1	20.925	N/A
NM_007186.28.25	CEP250	chr20	34084405	34087998	N/A	N/A	1	20.925	N/A
NM_000387.8.6	SLC25A20	chr3	48895939	48897060	N/A	N/A	1	20.925	N/A
NM_022748.27.21	TNS3	chr7	47331552	47344601	N/A	N/A	1	20.925	N/A
NM_017660.5.3	GATAD2A	chr19	19603114	19605192	N/A	N/A	1	20.925	N/A
NM_198514.5.3	NHLRC2	chr10	115636279	115644139	N/A	N/A	1	20.925	N/A
NM_014719.2.2	TCAF1	chr7	143573081	143573715	N/A	N/A	1	20.925	N/A
NR_037705.5.2	-	chr16	89287404	89292039	N/A	N/A	1	20.925	N/A

NM_005845.19.11	ABCC4	chr13	95813442	95839146	N/A	N/A	1	20.925	N/A
NM_145862.10.9	CHEK2	chr22	29091697	29095925	N/A	N/A	1	20.925	N/A
NM_020773.2.2	TBC1D14	chr4	6925099	6925838	N/A	N/A	1	20.925	N/A
NM_002568.14.12	PABPC1	chr8	101716524	101717901	N/A	N/A	1	20.925	N/A
NM_182641.7.5	BPTF	chr17	65887959	65890281	N/A	N/A	1	20.925	N/A
NM_000324.9.2	RHAG	chr6	49574560	49587075	N/A	N/A	1	20.925	N/A
NM_032167.9.5	SNX29	chr16	12136753	12155503	N/A	N/A	1	20.925	N/A
NM_145799.9.9	38961	chrX	118763280	118763471	N/A	N/A	1	20.925	N/A
NM_007342.3.2	NUPL2	chr7	23224688	23226765	N/A	N/A	1	20.925	N/A
NM_022897.24.23	RANBP17	chr5	170667931	170669824	N/A	N/A	1	20.925	N/A
NM_173872.5.3	CLCN3	chr4	170601200	170610381	N/A	N/A	1	20.925	N/A
NM_015255.34.32	UBR2	chr6	42630995	42633983	N/A	N/A	1	20.925	N/A
NM_006219.14.8	PIK3CB	chr3	138407716	138431146	N/A	N/A	1	20.925	N/A
NM_005899.14.11	NBR1	chr17	41345110	41347056	N/A	N/A	1	20.925	N/A
NM_003750.5.4	EIF3A	chr10	120830397	120832565	N/A	N/A	1	20.925	N/A
NM_001199692.7.3	SLC4A2	chr7	150761288	150764080	N/A	N/A	1	20.925	N/A
NM_014611.23.22	MDN1	chr6	90461149	90463341	N/A	N/A	1	20.925	N/A
NM_000632.14.7	ITGAM	chr16	31283167	31309275	N/A	N/A	1	20.925	N/A
NM_001278651.4.3	RANGAP1	chr22	41670603	41677086	N/A	N/A	1	20.925	N/A
NM_032815.5.4	NFATC2IP	chr16	28967298	28967658	N/A	N/A	1	20.925	N/A
NM_001211.22.13	BUB1B	chr15	40494605	40510763	N/A	N/A	1	20.925	N/A
NM_181672.5.4	OGT	chrX	70764416	70767873	N/A	N/A	1	20.925	N/A
NM_178835.4.3	ZNF827	chr4	146806829	146813567	N/A	N/A	1	20.925	N/A
NM_020718.11.2	USP31	chr16	23096180	23119504	N/A	N/A	1	20.925	N/A
NM_001144382.5.4	PLCL2	chr3	17051165	17056403	N/A	N/A	1	20.925	N/A
NM_000124.20.6	ERCC6	chr10	50668418	50714058	N/A	N/A	1	20.925	N/A
NR_029401.16.14	-	chr1	243222779	243229543	N/A	N/A	1	20.925	N/A
NM_024635.6.4	NAA35	chr9	88573386	88577095	N/A	N/A	1	20.925	N/A
NM_001193388.12.12	UNKL	chr16	1420130	1420358	N/A	N/A	1	20.925	N/A
NM_032139.10.6	ANKRD27	chr19	33132929	33134537	N/A	N/A	1	20.925	N/A
NM_001127391.7.2	ALS2CR12	chr2	202207092	202216174	N/A	N/A	1	20.925	N/A
NM_014709.53.22	USP34	chr2	61468694	61546462	N/A	N/A	1	20.925	N/A
NM_033395.7.2	CEP295	chr11	93399847	93412718	N/A	N/A	1	20.925	N/A

NR_015424.12.3	-	chr2	89076017	89092011	N/A	N/A	1	20.925	N/A
NM_152327.17.16	AK7	chr14	96949335	96953393	N/A	N/A	1	20.925	N/A
NM_032435.4.2	RP5-862P8.2	chr1	233482187	233490757	N/A	N/A	1	20.925	N/A
NM_001284333.6.3	TLK2	chr17	60598133	60601692	N/A	N/A	1	20.925	N/A
NM_001258374.23.23	EPS15L1	chr19	16472589	16472795	N/A	N/A	1	20.925	N/A
NM_001039840.3.2	CHIC1	chrX	72797242	72804408	N/A	N/A	1	20.925	N/A
NM_001160047.18.10	TXNDC16	chr14	52922041	52957723	N/A	N/A	1	20.925	N/A
NM_001145001.5.3	NEK6	chr9	127064214	127076264	N/A	N/A	1	20.925	N/A
NM_001244192.30.25	KIAA0586	chr14	58955338	58979345	N/A	N/A	1	20.925	N/A
NM_017437.15.2	CPSF2	chr14	92592465	92627590	N/A	N/A	1	20.925	N/A
NM_001195214.4.2	YIPF6	chrX	67733168	67741339	N/A	N/A	1	20.925	N/A
NM_001698.6.2	AUH	chr9	94058302	94118437	N/A	N/A	1	20.925	N/A
NM_012181.6.3	FKBP8	chr19	18648410	18650530	N/A	N/A	1	20.925	N/A
NR_033676.6.4	-	chrX	106390972	106396733	N/A	N/A	1	20.925	N/A
NM_000938.9.6	POLR2B	chr4	57861416	57871885	N/A	N/A	1	20.925	N/A
NM_198596.3.2	SULF2	chr20	46365446	46386207	N/A	N/A	1	20.925	N/A
NM_015542.21.15	UPF2	chr10	11971863	11994248	N/A	N/A	1	20.925	N/A
NM_002466.9.8	MYBL2	chr20	42331129	42333998	N/A	N/A	1	20.925	N/A
NM_017719.3.3	SNRK	chr3	43344589	43345284	N/A	N/A	1	20.925	N/A
NM_001284499.7.2	SLC43A2	chr17	1486497	1496573	N/A	N/A	1	20.925	N/A
NM_003105.5.4	SORL1	chr11	121358740	121360819	N/A	N/A	1	20.925	N/A
NM_001282801.15.14	SENP7	chr3	101058938	101060623	N/A	N/A	1	20.925	N/A
NM_015344.3.2	LEPROTL1	chr8	29959413	29962002	N/A	N/A	1	20.925	N/A
NM_005494.5.4	DNAJB6	chr7	157159195	157160177	N/A	N/A	1	20.925	N/A
NM_003703.12.5	NOP14	chr4	2946854	2955372	N/A	N/A	1	20.925	N/A
NM_001033553.12.9	SPECC1	chr17	20150531	20163607	N/A	N/A	1	20.925	N/A
NM_014822.22.20	SEC24D	chr4	119649715	119654067	N/A	N/A	1	20.925	N/A
NM_022145.6.5	CENPK	chr5	64838628	64847463	N/A	N/A	1	20.925	N/A
NR_104612.7.5	-	chr9	37768072	37770736	N/A	N/A	1	20.925	N/A
NM_138426.6.3	GLCCI1	chr7	8062112	8110761	N/A	N/A	1	20.925	N/A
NM_001004470.4.3	ST8SIA6	chr10	17401512	17432619	N/A	N/A	1	20.925	N/A
NM_000234.9.8	LIG1	chr19	48653019	48653467	N/A	N/A	1	20.925	N/A
NM_001024948.13.10	FNBP1L	chr1	94009663	94014983	N/A	N/A	1	20.925	N/A

NM_001134778.5.4	PBX3	chr9	128691933	128697886	N/A	N/A	1	20.925	N/A
NM_032320.6.3	BTBD10	chr11	13435076	13443385	N/A	N/A	1	20.925	N/A
NM_005475.2.2	SH2B3	chr12	111855922	111856681	N/A	N/A	1	20.925	N/A
NM_001257387.10.3	CHEK2	chr22	29095825	29121355	N/A	N/A	1	20.925	N/A
NM_032900.7.3	ARHGAP19	chr10	99006028	99024663	N/A	N/A	1	20.925	N/A
NM_001142936.7.6	DAGLB	chr7	6461357	6464466	N/A	N/A	1	20.925	N/A
NM_004459.25.23	BPTF	chr17	65941524	65944422	N/A	N/A	1	20.925	N/A
NM_207123.6.4	GAB1	chr4	144359151	144361535	N/A	N/A	1	20.925	N/A
NM_018489.6.6	ASH1L	chr1	155385534	155385714	N/A	N/A	1	20.925	N/A
NM_001494.6.2	GDI2	chr10	5827104	5842668	N/A	N/A	1	20.925	N/A
NM_001373.36.31	DNAH14	chr1	225339706	225373127	N/A	N/A	1	20.925	N/A
NM_000553.34.33	WRN	chr8	31014883	31024746	N/A	N/A	1	20.925	N/A
NM_014109.20.14	ATAD2	chr8	124351550	124361684	N/A	N/A	1	20.925	N/A
NM_174942.8.2	GAS2L3	chr12	100985513	101012365	N/A	N/A	1	20.925	N/A
NM_000324.5.4	RHAG	chr6	49582399	49583484	N/A	N/A	1	20.925	N/A
NM_015024.25.16	XPO7	chr8	21846513	21859783	N/A	N/A	1	20.925	N/A
NM_001172712.5.4	SP3	chr2	174783320	174820960	N/A	N/A	1	20.925	N/A
NM_014813.8.2	LRIG2	chr1	113633939	113638987	N/A	N/A	1	20.925	N/A
NM_001010887.4.2	ACER2	chr9	19423859	19435082	N/A	N/A	1	20.925	N/A
NM_194429.11.7	FGFR1OP	chr6	167435896	167447459	N/A	N/A	1	20.925	N/A
NM_001001894.42.38	TTC3	chr21	38559349	38568337	N/A	N/A	1	20.925	N/A
NM_022166.5.3	XYLT1	chr16	17292068	17353355	N/A	N/A	1	20.925	N/A
NM_001009955.8.6	SSBP3	chr1	54717288	54722859	N/A	N/A	1	20.925	N/A
NM_138798.6.4	MITD1	chr2	99786012	99787892	N/A	N/A	1	20.925	N/A
NM_001100409.20.18	SENP6	chr6	76412360	76421132	N/A	N/A	1	20.925	N/A
NM_020235.5.4	BBX	chr3	107429298	107435696	N/A	N/A	1	20.925	N/A
NM_001198665.35.31	ARHGEF12	chr11	120345268	120348235	N/A	N/A	1	20.925	N/A
NR_026580.4.4	-	chr13	114277494	114277601	N/A	N/A	1	20.925	N/A
NM_000875.2.2	IGF1R	chr15	99250790	99251336	N/A	N/A	1	20.925	N/A
NM_031921.7.3	ATAD3B	chr1	1414023	1417994	N/A	N/A	1	20.925	N/A
NM_001114636.5.2	FANCL	chr2	58449076	58459247	N/A	N/A	1	20.925	N/A
NM_032341.7.3	DDI2	chr1	15956819	15976318	N/A	N/A	1	20.925	N/A
NM_018367.7.5	ACER3	chr11	76696686	76709865	N/A	N/A	1	20.925	N/A

NM_003328.6.2	TXK	chr4	48106917	48116419	N/A	N/A	1	20.925	N/A
NM_007367.7.3	RALY	chr20	32659871	32665051	N/A	N/A	1	20.925	N/A
NM_005441.11.8	CHAF1B	chr21	37775055	37783902	N/A	N/A	1	20.925	N/A
NM_003157.15.13	NEK4	chr3	52771601	52775515	N/A	N/A	1	20.925	N/A
NM_030581.9.4	WDR59	chr16	74957823	74985441	N/A	N/A	1	20.925	N/A
NM_021190.8.4	PTBP2	chr1	97235258	97250810	N/A	N/A	1	20.925	N/A
NR_103780.8.2	-	chr4	77045801	77065626	N/A	N/A	1	20.925	N/A
NM_022111.20.15	CLSPN	chr1	36204736	36212593	N/A	N/A	1	20.925	N/A
NM_024757.25.16	EHMT1	chr9	140685299	140712590	N/A	N/A	1	20.925	N/A
NM_013396.7.4	USP25	chr21	17150222	17177577	N/A	N/A	1	20.925	N/A
NM_032421.6.5	CLIP2	chr7	73770739	73771807	N/A	N/A	1	20.925	N/A
NM_006811.4.2	SERINC3	chr20	43139929	43142681	N/A	N/A	1	20.925	N/A
NM_001173539.4.2	BANP	chr16	88008653	88017865	N/A	N/A	1	20.925	N/A
NM_004360.9.3	CDH1	chr16	68835572	68847398	N/A	N/A	1	20.925	N/A
NM_001033024.5.2	FBXO7	chr22	32875119	32883815	N/A	N/A	1	20.925	N/A
NM_016106.6.2	SCFD1	chr14	31097414	31109070	N/A	N/A	1	20.925	N/A
NM_030647.12.11	KDM7A	chr7	139801750	139810984	N/A	N/A	1	20.925	N/A
NM_001126050.5.2	HDGF	chr1	156713443	156715165	N/A	N/A	1	20.925	N/A
NM_005534.4.3	IFNGR2	chr21	34793786	34799339	N/A	N/A	1	20.925	N/A
NM_002631.8.5	PGD	chr1	10464217	10473308	N/A	N/A	1	20.925	N/A
NM_000375.9.7	UROS	chr10	127483448	127486714	N/A	N/A	1	20.925	N/A
NM_001080424.15.13	KDM6B	chr17	7753389	7754712	N/A	N/A	1	20.925	N/A
NM_003681.6.5	PDXK	chr21	45165959	45168961	N/A	N/A	1	20.925	N/A
NM_000210.24.21	ITGA6	chr2	173355751	173362828	N/A	N/A	1	20.925	N/A
NM_001177599.9.7	SUCLG2	chr3	67546221	67559327	N/A	N/A	1	20.925	N/A
NM_006292.7.2	TSG101	chr11	18524032	18541150	N/A	N/A	1	20.925	N/A
NM_001135638.14.13	PIP5K1A	chr1	151214598	151215043	N/A	N/A	1	20.925	N/A
NM_015113.16.15	ZZEF1	chr17	3988963	3989949	N/A	N/A	1	20.925	N/A
NM_032012.17.12	TMEM245	chr9	111795586	111819602	N/A	N/A	1	20.925	N/A
NM_006401.3.2	ANP32B	chr9	100756912	100760960	N/A	N/A	1	20.925	N/A
NM_139132.8.6	NUP98	chr11	3789810	3794969	N/A	N/A	1	20.925	N/A
NM_005788.7.6	PRMT3	chr11	20417348	20419267	N/A	N/A	1	20.925	N/A
NM_001256140.8.6	CAPG	chr2	85625142	85626408	N/A	N/A	1	20.925	N/A

NM_139132.8.7	NUP98	chr11	3789810	3793158	N/A	N/A	1	20.925	N/A
NM_001260.4.2	CDK8	chr13	26911703	26928017	N/A	N/A	1	20.925	N/A
NM_001077261.15.11	NCOR2	chr12	124904502	124915333	N/A	N/A	1	20.925	N/A
NM_006251.8.3	PRKAA1	chr5	40764615	40775605	N/A	N/A	1	20.925	N/A
NM_000265.4.3	NCF1	chr7	74193427	74193768	N/A	N/A	1	20.925	N/A
NM_001286431.7.5	REV3L	chr6	111726672	111737675	N/A	N/A	1	20.925	N/A
NM_001146156.9.8	GSK3B	chr3	119582265	119595355	N/A	N/A	1	20.925	N/A
NM_001243942.8.2	PRPSAP2	chr17	18768781	18814564	N/A	N/A	1	20.925	N/A
NM_024066.7.3	ERI3	chr1	44750506	44804994	N/A	N/A	1	20.925	N/A
NM_001014972.24.24	ZNF638	chr2	71653589	71654544	N/A	N/A	1	20.925	N/A
NM_152419.10.4	HGSNAT	chr8	43014065	43033377	N/A	N/A	1	20.925	N/A
NM_033505.6.2	EPT1	chr2	26587169	26598016	N/A	N/A	1	20.925	N/A
NM_032582.7.6	USP32	chr17	58346810	58348842	N/A	N/A	1	20.925	N/A
NM_017895.14.10	DDX27	chr20	47849842	47853047	N/A	N/A	1	20.925	N/A
NM_022486.11.10	SUSD1	chr9	114842353	114860942	N/A	N/A	1	20.925	N/A
NM_172373.8.6	ELF1	chr13	41515056	41518061	N/A	N/A	1	20.925	N/A
NM_021259.8.5	TMEM8A	chr16	425132	426812	N/A	N/A	1	20.925	N/A
NM_005935.11.8	AFF1	chr4	88026779	88036451	N/A	N/A	1	20.925	N/A
NM_175854.13.7	PAN3	chr13	28830428	28845003	N/A	N/A	1	20.925	N/A
NM_001242899.3.2	PPP6R2	chr22	50810448	50832564	N/A	N/A	1	20.925	N/A
NM_002115.14.13	HK3	chr5	176311039	176314122	N/A	N/A	1	20.925	N/A
NM_003693.10.9	SCARF1	chr17	1540002	1540356	N/A	N/A	1	20.925	N/A
NM_002508.12.10	NID1	chr1	236175220	236180573	N/A	N/A	1	20.925	N/A
NM_001110781.7.6	SLC35E2B	chr1	1601102	1601590	N/A	N/A	1	20.925	N/A
NM_153050.8.3	MTMR3	chr22	30366964	30394846	N/A	N/A	1	20.925	N/A
NM_031844.12.11	HNRNPU	chr1	245018725	245019460	N/A	N/A	1	20.925	N/A
NM_001164161.19.10	PPP6R3	chr11	68334481	68363686	N/A	N/A	1	20.925	N/A
NM_178450.4.2	37681	chr5	126213876	126253919	N/A	N/A	1	20.925	N/A
NM_152227.6.5	SNX5	chr20	17933230	17934761	N/A	N/A	1	20.925	N/A
NM_001130823.13.6	DNMT1	chr19	10273342	10288043	N/A	N/A	1	20.925	N/A
NM_001128212.9.2	WDSUB1	chr2	160112706	160139604	N/A	N/A	1	20.925	N/A
NM_001289403.7.4	MMS19	chr10	99236439	99238146	N/A	N/A	1	20.925	N/A
NM_014676.14.14	PUM1	chr1	31437520	31437757	N/A	N/A	1	20.925	N/A

NM_001099271.10.5	POC5	chr5	74981031	74998635	N/A	N/A	1	20.925	N/A
NM_018027.19.16	FRMD4A	chr10	13705452	13717044	N/A	N/A	1	20.925	N/A
NM_001128148.16.10	TFRC	chr3	195785154	195792471	N/A	N/A	1	20.925	N/A
NR_003187.8.3	-	chr7	74576463	74582682	N/A	N/A	1	20.925	N/A
NM_014616.25.22	ATP11B	chr3	182602540	182607336	N/A	N/A	1	20.925	N/A
NM_001177387.3.1	ATXN7	chr3	63884074	63898901	N/A	N/A	1	20.925	N/A
NM_001166691.16.13	TTK	chr6	80737601	80745134	N/A	N/A	1	20.925	N/A
NM_024570.9.2	RNASEH2B	chr13	51501542	51523641	N/A	N/A	1	20.925	N/A
NM_001278716.9.2	FBXL4	chr6	99323290	99382755	N/A	N/A	1	20.925	N/A
NM_001167582.13.12	RBM6	chr3	50102463	50103935	N/A	N/A	1	20.925	N/A
NM_017772.10.9	TBC1D22B	chr6	37280693	37281667	N/A	N/A	1	20.925	N/A
NM_001256667.14.7	FAM193A	chr4	2659529	2692697	N/A	N/A	1	20.925	N/A
NM_001010989.6.5	HERPUD1	chr16	56973148	56974157	N/A	N/A	1	20.925	N/A
NM_000254.8.3	MTR	chr1	236969443	236979843	N/A	N/A	1	20.925	N/A
NM_001206704.7.4	SP100	chr2	231307651	231313865	N/A	N/A	1	20.925	N/A
NM_020846.5.3	SLAIN2	chr4	48379912	48384944	N/A	N/A	1	20.925	N/A
NM_032590.12.7	KDM2B	chr12	121932381	121972495	N/A	N/A	1	20.925	N/A
NM_015355.10.7	SUZ12	chr17	30302500	30315516	N/A	N/A	1	20.925	N/A
NM_020954.11.9	RNF213	chr17	78268518	78272318	N/A	N/A	1	20.925	N/A
NM_138495.2.2	ATXN7L1	chr7	105305512	105305735	N/A	N/A	1	20.925	N/A
NM_203437.6.3	AFTPH	chr2	64794695	64800202	N/A	N/A	1	20.925	N/A
NM_005761.5.3	PLXNC1	chr12	94575221	94603480	N/A	N/A	1	20.925	N/A
NM_006699.6.2	MAN1A2	chr1	117944807	117984947	N/A	N/A	1	20.925	N/A
NM_002158.3.2	FOXN2	chr2	48555699	48573890	N/A	N/A	1	20.925	N/A
NM_015626.6.3	WSB1	chr17	25630392	25636298	N/A	N/A	1	20.925	N/A
NM_006733.14.13	CENPI	chrX	100387178	100387445	N/A	N/A	1	20.925	N/A
NM_012335.4.2	MYO1F	chr19	8619360	8620680	N/A	N/A	1	20.925	N/A
NM_020943.19.18	CWC22	chr2	180815238	180815653	N/A	N/A	1	20.925	N/A
NM_175854.17.7	PAN3	chr13	28830428	28855516	N/A	N/A	1	20.925	N/A
NM_015348.40.32	TMEM131	chr2	98375355	98392481	N/A	N/A	1	20.925	N/A
NM_018449.12.7	UBAP2	chr9	33953282	33973235	N/A	N/A	1	20.925	N/A
NM_024757.18.17	EHMT1	chr9	140693264	140695436	N/A	N/A	1	20.925	N/A
NM_005474.16.13	HDAC5	chr17	42161922	42165064	N/A	N/A	1	20.925	N/A

NM_001257137.21.16	ITCH	chr20	33057852	33069011	N/A	N/A	1	20.925	N/A
NM_002041.6.5	GABPB1	chr15	50592985	50593565	N/A	N/A	1	20.925	N/A
NM_018052.12.11	VAC14	chr16	70796442	70796928	N/A	N/A	1	20.925	N/A
NM_004398.12.11	DDX10	chr11	108586605	108590612	N/A	N/A	1	20.925	N/A
NM_007199.8.2	IRAK3	chr12	66597490	66622150	N/A	N/A	1	20.925	N/A
NM_001206947.12.10	PICALM	chr11	85707868	85712201	N/A	N/A	1	20.925	N/A
NM_003400.9.7	XPO1	chr2	61725807	61727029	N/A	N/A	1	20.925	N/A
NM_030816.9.6	ANKRD13C	chr1	70758070	70771973	N/A	N/A	1	20.925	N/A
NM_020193.7.5	EMSY	chr11	76169226	76175124	N/A	N/A	1	20.925	N/A
NM_018090.7.6	NECAP2	chr1	16778332	16782388	N/A	N/A	1	20.925	N/A
NM_003074.26.22	SMARCC1	chr3	47651555	47680270	N/A	N/A	1	20.925	N/A
NM_025004.13.10	CCDC15	chr11	124862475	124875108	N/A	N/A	1	20.925	N/A
NM_139132.14.11	NUP98	chr11	3752620	3774638	N/A	N/A	1	20.925	N/A
NM_001282771.8.4	ANKMY1	chr2	241463301	241492474	N/A	N/A	1	20.925	N/A
NR_102333.7.3	-	chr19	42311148	42314935	N/A	N/A	1	20.925	N/A
NM_024809.9.7	TCTN2	chr12	124172597	124177270	N/A	N/A	1	20.925	N/A
NM_001144823.11.3	DENND4A	chr15	66021409	66048810	N/A	N/A	1	20.925	N/A
NM_001006941.5.2	ALG3	chr3	183962388	183963600	N/A	N/A	1	20.925	N/A
NM_006516.4.3	SLC2A1	chr1	43396296	43396877	N/A	N/A	1	20.925	N/A
NM_001199699.9.8	DHX33	chr17	5353522	5354254	N/A	N/A	1	20.925	N/A
NM_000181.11.5	GUSB	chr7	65429309	65441189	N/A	N/A	1	20.925	N/A
NM_139241.6.5	FGD4	chr12	32751430	32754357	N/A	N/A	1	20.925	N/A
NM_004726.17.15	REPS2	chrX	17151946	17157084	N/A	N/A	1	20.925	N/A
NM_015346.35.33	ZFYVE26	chr14	68228082	68229536	N/A	N/A	1	20.925	N/A
NM_001746.6.2	CANX	chr5	179132679	179136060	N/A	N/A	1	20.925	N/A
NM_005923.8.4	MAP3K5	chr6	136990420	137019820	N/A	N/A	1	20.925	N/A
NM_004397.4.2	DDX6	chr11	118650340	118657227	N/A	N/A	1	20.925	N/A
NM_003502.5.4	AXIN1	chr16	354303	360069	N/A	N/A	1	20.925	N/A
NM_015091.5.3	FAM179B	chr14	45468565	45475470	N/A	N/A	1	20.925	N/A
NM_001142300.5.2	CCNYL1	chr2	208589523	208607066	N/A	N/A	1	20.925	N/A
NM_030647.10.7	KDM7A	chr7	139813290	139824583	N/A	N/A	1	20.925	N/A
NM_030819.2.2	GFOD2	chr16	67719359	67719705	N/A	N/A	1	20.925	N/A
NR_024497.6.5	-	chr10	38727785	38734535	N/A	N/A	1	20.925	N/A

NM_032773.4.2	LRCH3	chr3	197541778	197547301	N/A	N/A	1	20.925	N/A
NM_002209.6.5	ITGAL	chr16	30490411	30490782	N/A	N/A	1	20.925	N/A
NM_001195555.10.9	CLINT1	chr5	157218710	157221980	N/A	N/A	1	20.925	N/A
NM_015239.25.18	AGTPBP1	chr9	88190229	88211364	N/A	N/A	1	20.925	N/A
NM_015395.17.16	TECPR1	chr7	97857325	97858478	N/A	N/A	1	20.925	N/A
NM_001282806.5.2	39142	chr2	160585519	160609082	N/A	N/A	1	20.925	N/A
NM_018201.9.8	TBC1D13	chr9	131565528	131566398	N/A	N/A	1	20.925	N/A
NM_001754.7.5	RUNX1	chr21	36206706	36253010	N/A	N/A	1	20.925	N/A
NM_015033.10.7	FNBP1	chr9	132686122	132691974	N/A	N/A	1	20.925	N/A
NM_199141.14.4	CARM1	chr19	11019778	11031803	N/A	N/A	1	20.925	N/A
NM_015542.8.4	UPF2	chr10	12039670	12056183	N/A	N/A	1	20.925	N/A
NM_001289003.14.7	PHLPP2	chr16	71692135	71713438	N/A	N/A	1	20.925	N/A
NM_001145112.7.6	PATL2	chr15	44964212	44964654	N/A	N/A	1	20.925	N/A
NM_030752.6.2	TCP1	chr6	160205697	160209175	N/A	N/A	1	20.925	N/A
NM_173638.3.1	NBPF15	chr1	148560846	148568877	N/A	N/A	1	20.925	N/A
NM_016604.12.8	KDM3B	chr5	137726701	137750928	N/A	N/A	1	20.925	N/A
NM_033071.107.104	SYNE1	chr6	152560667	152570397	N/A	N/A	1	20.925	N/A
NM_016955.6.2	SEPSECS	chr4	25153581	25160729	N/A	N/A	1	20.925	N/A
NM_032043.10.8	BRIP1	chr17	59870957	59878835	N/A	N/A	1	20.925	N/A
NM_001282919.5.3	SPIDR	chr8	48196616	48206619	N/A	N/A	1	20.925	N/A
NM_001206736.7.2	NSFL1C	chr20	1433137	1445071	N/A	N/A	1	20.925	N/A
NM_001282753.11.9	TLE4	chr9	82319697	82323701	N/A	N/A	1	20.925	N/A
NM_004843.4.3	IL27RA	chr19	14150319	14150722	N/A	N/A	1	20.925	N/A
NM_012305.16.15	AP2A2	chr11	1000431	1003804	N/A	N/A	1	20.925	N/A
NM_014630.3.2	ZNF592	chr15	85307937	85321543	N/A	N/A	1	20.925	N/A
NM_024820.9.2	DENND1A	chr9	126414291	126641300	N/A	N/A	1	20.925	N/A
NM_013255.6.3	MKLN1	chr7	131071878	131084192	N/A	N/A	1	20.925	N/A
NM_152608.6.6	SDE2	chr1	226175596	226176089	N/A	N/A	1	20.925	N/A
NM_007318.3.2	PSEN1	chr14	73614502	73614802	N/A	N/A	1	20.925	N/A
NM_016166.6.3	PIAS1	chr15	68434283	68439038	N/A	N/A	1	20.925	N/A
NM_153813.3.2	ZFPM1	chr16	88552346	88555561	N/A	N/A	1	20.925	N/A
NM_001145160.6.3	TPM4	chr19	16192722	16199930	N/A	N/A	1	20.925	N/A
NM_015243.6.4	VPS13B	chr8	100108539	100123507	N/A	N/A	1	20.925	N/A

NM_030808.7.2	NDEL1	chr17	8347577	8358205	N/A	N/A	1	20.925	N/A
NM_053005.4.3	MOB2	chr11	1501622	1502115	N/A	N/A	1	20.925	N/A
NM_024813.12.9	RPAP2	chr1	92798947	92846430	N/A	N/A	1	20.925	N/A
NM_001193476.11.10	SLC26A8	chr6	35936653	35943265	N/A	N/A	1	20.925	N/A
NM_007282.8.3	RNF13	chr3	149563797	149629870	N/A	N/A	1	20.925	N/A
NM_006733.17.16	CENPI	chrX	100400052	100401266	N/A	N/A	1	20.925	N/A
NM_018261.17.15	EXOC1	chr4	56759717	56766050	N/A	N/A	1	20.925	N/A
NM_138771.3.3	CCDC126	chr7	23650789	23651172	N/A	N/A	1	20.925	N/A
NR_040723.12.8	-	chr20	34302106	34313077	N/A	N/A	1	20.925	N/A
NM_018190.15.13	BBS7	chr4	122754385	122760851	N/A	N/A	1	20.925	N/A
NR_024460.10.8	-	chr3	56626997	56628056	N/A	N/A	1	20.925	N/A
NM_006031.27.25	PCNT	chr21	47819503	47822397	N/A	N/A	1	20.925	N/A
NM_001278209.18.13	NUP153	chr6	17632880	17649531	N/A	N/A	1	20.925	N/A
NM_000052.4.3	ATP7A	chrX	77243737	77245454	N/A	N/A	1	20.925	N/A
NR_073547.14.10	-	chr14	79423584	79454484	N/A	N/A	1	20.925	N/A
NM_020409.6.2	MRPL47	chr3	179310431	179320585	N/A	N/A	1	20.925	N/A
NM_006437.12.2	PARP4	chr13	25058790	25077915	N/A	N/A	1	20.925	N/A
NM_001605.13.12	AARS	chr16	70294946	70296427	N/A	N/A	1	20.925	N/A
NM_199203.4.3	TMEM189-UBE2V1	chr20	48746082	48747484	N/A	N/A	1	20.925	N/A
NM_006437.12.8	PARP4	chr13	25058790	25067871	N/A	N/A	1	20.925	N/A
NM_173496.13.6	MPP7	chr10	28408579	28438988	N/A	N/A	1	20.925	N/A
NM_001282938.12.4	STIL	chr1	47753222	47770668	N/A	N/A	1	20.925	N/A
NM_052985.8.4	IFT122	chr3	129177441	129183624	N/A	N/A	1	20.925	N/A
NM_001286790.4.2	CNOT6L	chr4	78694234	78697546	N/A	N/A	1	20.925	N/A
NM_145647.11.9	TBC1D31	chr8	124121556	124132428	N/A	N/A	1	20.925	N/A
NR_034059.15.7	-	chr2	231940224	231951895	N/A	N/A	1	20.925	N/A
NM_000436.16.13	OXCT1	chr5	41739491	41794180	N/A	N/A	1	20.925	N/A
NR_027862.3.2	-	chr2	172782046	172809519	N/A	N/A	1	20.925	N/A
NM_005898.11.10	CAPRIN1	chr11	34107610	34107960	N/A	N/A	1	20.925	N/A
NM_001166005.16.10	EPB41	chr1	29362337	29391670	N/A	N/A	1	20.925	N/A
NM_001256135.23.19	CSE1L	chr20	47707278	47711500	N/A	N/A	1	20.925	N/A
NM_016281.14.12	TAOK3	chr12	118627611	118639268	N/A	N/A	1	20.925	N/A
NM_024790.3.2	CSPP1	chr8	67986477	67988816	N/A	N/A	1	20.925	N/A

NM_021629.8.2	GNB4	chr3	179131199	179144030	N/A	N/A	1	20.925	N/A
NM_021629.8.5	GNB4	chr3	179131199	179134344	N/A	N/A	1	20.925	N/A
NM_152671.5.4	PIKFYVE	chr2	209150449	209153542	N/A	N/A	1	20.925	N/A
NM_020840.12.11	FNIP2	chr4	159782493	159782928	N/A	N/A	1	20.925	N/A
NM_003648.17.15	DGKD	chr2	234358627	234360706	N/A	N/A	1	20.925	N/A
NR_002453.11.7	-	chr16	30299881	30306012	N/A	N/A	1	20.925	N/A
NM_025083.3.2	EDC3	chr15	74963795	74967483	N/A	N/A	1	20.925	N/A
NM_012430.6.3	SEC22A	chr3	122942405	122978436	N/A	N/A	1	20.925	N/A
NM_199166.7.5	ALAS1	chr3	52238708	52240767	N/A	N/A	1	20.925	N/A
NM_182641.2.2	BPTF	chr17	65850055	65850878	N/A	N/A	1	20.925	N/A
NM_006754.5.3	SYPL1	chr7	105733394	105739728	N/A	N/A	1	20.925	N/A
NM_001247996.10.4	ASAP1	chr8	131191509	131370389	N/A	N/A	1	20.925	N/A
NM_004738.5.4	VAPB	chr20	57014000	57016139	N/A	N/A	1	20.925	N/A
NR_027949.15.12	-	chr12	1480998	1553916	N/A	N/A	1	20.925	N/A
NM_001286189.6.4	SCAF8	chr6	155095122	155109156	N/A	N/A	1	20.925	N/A
NM_001131010.9.2	SATB1	chr3	18419661	18462483	N/A	N/A	1	20.925	N/A
NM_001113492.3.3	40057	chr17	75398140	75398785	N/A	N/A	1	20.925	N/A
NM_000651.27.5	CR1	chr1	207696955	207743291	N/A	N/A	1	20.925	N/A
NM_014415.3.2	ZBTB11	chr3	101389973	101391057	N/A	N/A	1	20.925	N/A
NM_001284363.9.6	TLK2	chr17	60629662	60642498	N/A	N/A	1	20.925	N/A
NM_001876.6.3	CPT1A	chr11	68566685	68580044	N/A	N/A	1	20.925	N/A
NM_001161344.12.11	CHFR	chr12	133428203	133430159	N/A	N/A	1	20.925	N/A
NR_027671.11.2	-	chr2	128855002	128880820	N/A	N/A	1	20.925	N/A
NM_001195215.10.6	DENND1B	chr1	197611840	197627499	N/A	N/A	1	20.925	N/A
NM_001286277.11.8	TUBGCP3	chr13	113200012	113208496	N/A	N/A	1	20.925	N/A
NM_013448.3.3	BAZ1A	chr14	35331249	35331528	N/A	N/A	1	20.925	N/A
NM_002111.6.2	HTT	chr4	3088665	3109150	N/A	N/A	1	20.925	N/A
NM_018706.16.2	DHTKD1	chr10	12123470	12162266	N/A	N/A	1	20.925	N/A
NM_003400.24.21	XPO1	chr2	61708319	61711240	N/A	N/A	1	20.925	N/A
NM_003384.11.4	VRK1	chr14	97312431	97327072	N/A	N/A	1	20.925	N/A
NM_004333.13.4	BRAF	chr7	140476711	140508795	N/A	N/A	1	20.925	N/A
NM_031217.10.4	KIF18A	chr11	28098553	28113060	N/A	N/A	1	20.925	N/A
NM_014864.4.2	FAM20B	chr1	179012849	179023773	N/A	N/A	1	20.925	N/A

NM_002661.5.3	PLCG2	chr16	81888048	81892768	N/A	N/A	1	20.925	N/A
NM_020314.20.13	C16orf62	chr16	19627435	19648978	N/A	N/A	1	20.925	N/A
NM_014023.11.6	WDR37	chr10	1130342	1151207	N/A	N/A	1	20.925	N/A
NM_025160.6.4	WDR26	chr1	224605961	224612356	N/A	N/A	1	20.925	N/A
NM_030877.7.6	CTNNBL1	chr20	36393598	36396446	N/A	N/A	1	20.925	N/A
NM_014153.9.4	ZC3H7A	chr16	11868091	11873219	N/A	N/A	1	20.925	N/A
NM_015435.9.7	RNF19A	chr8	101273769	101277013	N/A	N/A	1	20.925	N/A
NM_014823.23.18	WNK1	chr12	996386	1006847	N/A	N/A	1	20.925	N/A
NM_032156.8.4	CAPRIN2	chr12	30881581	30888140	N/A	N/A	1	20.925	N/A
NM_001244585.3.2	LUC7L2	chr7	139056132	139060902	N/A	N/A	1	20.925	N/A
NM_018682.11.6	KMT2E	chr7	104714065	104719410	N/A	N/A	1	20.925	N/A
NM_004286.4.2	GTPBP1	chr22	39104849	39113005	N/A	N/A	1	20.925	N/A
NM_003900.5.3	SQSTM1	chr5	179250857	179252226	N/A	N/A	1	20.925	N/A
NM_001076785.5.4	SLC7A6	chr16	68308593	68321774	N/A	N/A	1	20.925	N/A
NM_004145.10.9	MYO9B	chr19	17273159	17273932	N/A	N/A	1	20.925	N/A
NM_138962.11.3	MSI2	chr17	55334826	55729522	N/A	N/A	1	20.925	N/A
NM_012197.13.2	RABGAP1	chr9	125719289	125782738	N/A	N/A	1	20.925	N/A
NM_145177.3.2	DHRSX	chrY	2276785	2293345	N/A	N/A	1	20.925	N/A
NM_001164687.3.3	THEMIS	chr6	128150620	128151079	N/A	N/A	1	20.925	N/A
NM_014801.17.10	PCNX2	chr1	233313547	233363117	N/A	N/A	1	20.925	N/A
NM_024820.12.2	DENND1A	chr9	126345447	126641300	N/A	N/A	1	20.925	N/A
NM_001135055.4.2	TKT	chr3	53274266	53276258	N/A	N/A	1	20.925	N/A
NM_001178056.14.8	PARP8	chr5	50073902	50093067	N/A	N/A	1	20.925	N/A
NM_138619.8.5	GGA3	chr17	73238416	73239651	N/A	N/A	1	20.925	N/A
NM_000309.8.7	PPOX	chr1	161138782	161139510	N/A	N/A	1	20.925	N/A
NM_018449.14.11	UBAP2	chr9	33944362	33956144	N/A	N/A	1	20.925	N/A
NM_032221.5.2	CHD6	chr20	40141484	40179999	N/A	N/A	1	20.925	N/A
NM_005845.13.5	ABCC4	chr13	95829960	95863035	N/A	N/A	1	20.925	N/A
NM_001005751.13.12	FAM21A	chr10	51853027	51853677	N/A	N/A	1	20.925	N/A
NM_018449.14.13	UBAP2	chr9	33944362	33948585	N/A	N/A	1	20.925	N/A
NM_144604.8.8	ZC3H18	chr16	88677675	88677944	N/A	N/A	1	20.925	N/A
NM_001243089.5.4	FOXM1	chr12	2975558	2977920	N/A	N/A	1	20.925	N/A
NM_017890.31.22	VPS13B	chr8	100443764	100568881	N/A	N/A	1	20.925	N/A

NM_002541.20.19	OGDH	chr7	44739739	44741214	N/A	N/A	1	20.925	N/A
NM_001242599.11.6	RBM39	chr20	34300940	34313077	N/A	N/A	1	20.925	N/A
NM_032341.8.6	DDI2	chr1	15970016	15978390	N/A	N/A	1	20.925	N/A
NM_030934.7.2	TRMT1L	chr1	185112488	185121067	N/A	N/A	1	20.925	N/A
NM_005109.11.2	OXSR1	chr3	38224493	38278452	N/A	N/A	1	20.925	N/A
NM_032341.8.2	DDI2	chr1	15953163	15978390	N/A	N/A	1	20.925	N/A
NM_012335.12.5	MYO1F	chr19	8612919	8618321	N/A	N/A	1	20.925	N/A
NM_001077691.13.10	ALG9	chr11	111706887	111711532	N/A	N/A	1	20.925	N/A
NR_047701.7.6	-	chr7	43679047	43680248	N/A	N/A	1	20.925	N/A
NM_012426.21.19	SF3B3	chr16	70598967	70601439	N/A	N/A	1	20.925	N/A
NM_031490.6.4	LONP2	chr16	48292528	48296783	N/A	N/A	1	20.925	N/A
NM_024948.10.2	FAM188A	chr10	15858833	15889942	N/A	N/A	1	20.925	N/A
NM_014607.6.2	UBXN4	chr2	136505836	136519481	N/A	N/A	1	20.925	N/A
NM_001003941.4.3	OGDH	chr7	44684925	44687358	N/A	N/A	1	20.925	N/A
NM_020820.18.16	PREX1	chr20	47273577	47276599	N/A	N/A	1	20.925	N/A
NM_032844.6.5	MASTL	chr10	27453992	27454468	N/A	N/A	1	20.925	N/A
NM_030920.5.4	ANP32E	chr1	150198939	150201570	N/A	N/A	1	20.925	N/A
NM_012287.15.14	ACAP2	chr3	195022303	195022903	N/A	N/A	1	20.925	N/A
NM_001243403.20.20	CLEC16A	chr16	11217598	11217803	N/A	N/A	1	20.925	N/A
NM_001128610.7.4	USP8	chr15	50741596	50757388	N/A	N/A	1	20.925	N/A
NM_001198810.14.8	SLC43A1	chr11	57254567	57261644	N/A	N/A	1	20.925	N/A
NM_001144823.14.8	DENND4A	chr15	66007815	66025156	N/A	N/A	1	20.925	N/A
NM_002745.4.2	MAPK1	chr22	22153300	22162135	N/A	N/A	1	20.925	N/A
NM_001130850.4.2	CAB39	chr2	231624673	231658046	N/A	N/A	1	20.925	N/A
NM_001077352.4.3	RBM23	chr14	23375403	23378804	N/A	N/A	1	20.925	N/A
NM_001278210.14.12	NUP153	chr6	17646297	17649531	N/A	N/A	1	20.925	N/A
NM_033481.4.2	FBXO9	chr6	52935854	52941341	N/A	N/A	1	20.925	N/A
NM_002154.15.8	HSPA4	chr5	132422473	132432978	N/A	N/A	1	20.925	N/A
NM_001287061.8.6	RABGEF1	chr7	66262360	66270383	N/A	N/A	1	20.925	N/A
NR_026644.4.2	-	chr13	99890680	99896878	N/A	N/A	1	20.925	N/A
NM_005121.9.2	MED13	chr17	60087910	60140662	N/A	N/A	1	20.925	N/A
NM_013438.5.2	UBQLN1	chr9	86293355	86301070	N/A	N/A	1	20.925	N/A
NM_017566.5.2	KLHDC4	chr16	87782278	87795646	N/A	N/A	1	20.925	N/A

NM_017590.13.10	ZC3H7B	chr22	41735819	41739580	N/A	N/A	1	20.925	N/A
NM_014810.8.4	CEP350	chr1	179959641	179975702	N/A	N/A	1	20.925	N/A
NM_002577.12.11	PAK2	chr3	196541321	196545027	N/A	N/A	1	20.925	N/A
NM_001278387.6.3	SLC38A1	chr12	46622935	46637097	N/A	N/A	1	20.925	N/A
NM_001146216.3.2	TACC1	chr8	38646221	38678153	N/A	N/A	1	20.925	N/A
NM_002432.4.2	MNDA	chr1	158811923	158813912	N/A	N/A	1	20.925	N/A
NM_024524.29.21	ATP13A3	chr3	194146070	194152621	N/A	N/A	1	20.925	N/A
NM_003601.16.15	SMARCA5	chr4	144464661	144465125	N/A	N/A	1	20.925	N/A
NM_024605.9.5	ARHGAP10	chr4	148778703	148800489	N/A	N/A	1	20.925	N/A
NM_175710.9.5	CR1L	chr1	207867697	207874963	N/A	N/A	1	20.925	N/A
NR_073030.5.2	-	chr10	51606987	51613311	N/A	N/A	1	20.925	N/A
NM_001142495.5.2	MYO5A	chr15	52708341	52725482	N/A	N/A	1	20.925	N/A
NM_002504.17.16	NFX1	chr9	33351557	33352717	N/A	N/A	1	20.925	N/A
NM_182540.8.3	INTS6L	chrX	134679347	134686071	N/A	N/A	1	20.925	N/A
NM_004249.6.5	RAB28	chr4	13378168	13383218	N/A	N/A	1	20.925	N/A
NM_014868.13.4	RNF10	chr12	120992520	121004783	N/A	N/A	1	20.925	N/A
NM_000254.15.2	MTR	chr1	236966727	237001899	N/A	N/A	1	20.925	N/A
NM_022039.4.2	FBXW4	chr10	103432671	103436193	N/A	N/A	1	20.925	N/A
NM_001097615.3.2	POLR2J3	chr7	102208456	102210371	N/A	N/A	1	20.925	N/A
NM_018027.21.19	FRMD4A	chr10	13701322	13705509	N/A	N/A	1	20.925	N/A
NM_001876.10.8	CPT1A	chr11	68552282	68562379	N/A	N/A	1	20.925	N/A
NM_144973.7.6	DENND5B	chr12	31595708	31600703	N/A	N/A	1	20.925	N/A
NM_005499.5.2	UBA2	chr19	34921480	34925873	N/A	N/A	1	20.925	N/A
NM_182751.5.4	MCM10	chr10	13214375	13214765	N/A	N/A	1	20.925	N/A
NM_016377.6.2	AKAP7	chr6	131466424	131520713	N/A	N/A	1	20.925	N/A
NM_007167.4.3	ZMYM6	chr1	35484953	35486068	N/A	N/A	1	20.925	N/A
NM_004536.9.7	NAIP	chr5	70294550	70297970	N/A	N/A	1	20.925	N/A
NM_175854.5.2	PAN3	chr13	28748408	28771483	N/A	N/A	1	20.925	N/A
NM_001100164.6.6	PHACTR2	chr6	144086397	144086935	N/A	N/A	1	20.925	N/A
NM_003400.11.8	XPO1	chr2	61722589	61726048	N/A	N/A	1	20.925	N/A
NM_001005751.5.3	FAM21A	chr10	51829306	51840691	N/A	N/A	1	20.925	N/A
NM_024605.19.15	ARHGAP10	chr4	148867767	148944564	N/A	N/A	1	20.925	N/A
NM_001203249.8.4	EZH2	chr7	148523560	148529842	N/A	N/A	1	20.925	N/A

NM_001981.12.9	EPS15	chr1	51906018	51913807	N/A	N/A	1	20.925	N/A
NM_004996.4.2	ABCC1	chr16	16101672	16108485	N/A	N/A	1	20.925	N/A
NM_002463.5.2	MX2	chr21	42748762	42754491	N/A	N/A	1	20.925	N/A
NM_001080480.3.2	MBOAT1	chr6	20151415	20153000	N/A	N/A	1	20.925	N/A
NM_005085.30.28	NUP214	chr9	134070619	134077104	N/A	N/A	1	20.925	N/A
NM_001013848.18.8	EXOC6	chr10	94679738	94733989	N/A	N/A	1	20.925	N/A
NM_018639.6.3	WSB2	chr12	118474142	118481182	N/A	N/A	1	20.925	N/A
NM_001166168.7.3	NEK6	chr9	127074787	127089724	N/A	N/A	1	20.925	N/A
NM_152829.6.3	TES	chr7	115889073	115892530	N/A	N/A	1	20.925	N/A
NM_152787.9.7	TAB3	chrX	30861082	30871055	N/A	N/A	1	20.925	N/A
NM_001282751.9.4	SUCO	chr1	172525008	172539870	N/A	N/A	1	20.925	N/A
NM_001145207.4.3	HBS1L	chr6	135360710	135363264	N/A	N/A	1	20.925	N/A
NM_001136031.7.2	ATG7	chr3	11340159	11356967	N/A	N/A	1	20.925	N/A
NM_015016.17.15	MAST3	chr19	18245388	18246651	N/A	N/A	1	20.925	N/A
NM_000342.19.18	SLC4A1	chr17	42328526	42328956	N/A	N/A	1	20.925	N/A
NM_000051.52.49	ATM	chr11	108199747	108202764	N/A	N/A	1	20.925	N/A
NM_013385.8.7	CYTH4	chr22	37696947	37699443	N/A	N/A	1	20.925	N/A
NM_001006607.7.3	LRRC37A2	chr17_ctg5_hap1	212478	233157	N/A	N/A	1	20.925	N/A
NM_001286206.13.12	CEP162	chr6	84894904	84896341	N/A	N/A	1	20.925	N/A
NM_003704.13.12	FAM193A	chr4	2691232	2692697	N/A	N/A	1	20.925	N/A
NM_022373.8.2	HERPUD2	chr7	35673921	35734237	N/A	N/A	1	20.925	N/A
NM_198513.5.2	PHF20L1	chr8	133790041	133811106	N/A	N/A	1	20.925	N/A
NR_034181.2.2	-	chr10	14938844	14939516	N/A	N/A	1	20.925	N/A
NM_017858.6.5	TIPIN	chr15	66641397	66641775	N/A	N/A	1	20.925	N/A
NM_001039476.4.2	NPRL3	chr16	160522	180590	N/A	N/A	1	20.925	N/A
NM_001429.10.3	EP300	chr22	41521867	41537226	N/A	N/A	1	20.925	N/A
NM_005426.13.13	TP53BP2	chr1	223985916	223986379	N/A	N/A	1	20.925	N/A
NM_003126.35.34	SPTA1	chr1	158609371	158609797	N/A	N/A	1	20.925	N/A
NM_006045.13.5	ATP9A	chr20	50286535	50314021	N/A	N/A	1	20.925	N/A
NM_001139459.2.2	CNST	chr1	246754813	246755243	N/A	N/A	1	20.925	N/A
NM_006031.15.14	PCNT	chr21	47783394	47787054	N/A	N/A	1	20.925	N/A
NM_001005339.4.2	RGS10	chr10	121275020	121286936	N/A	N/A	1	20.925	N/A
NM_015496.4.3	KIAA1429	chr8	95549330	95550574	N/A	N/A	1	20.925	N/A

NM_004996.14.13	ABCC1	chr16	16162012	16165586	N/A	N/A	1	20.925	N/A
NM_007007.8.3	CPSF6	chr12	69646830	69653977	N/A	N/A	1	20.925	N/A
NR_046188.4.2	-	chr1	146066114	146074129	N/A	N/A	1	20.925	N/A
NR_024548.5.4	-	chr7	65595730	65599361	N/A	N/A	1	20.925	N/A
NR_033959.5.2	-	chr16	70253814	70256383	N/A	N/A	1	20.925	N/A
NR_002824.7.4	-	chr15	23333848	23339028	N/A	N/A	1	20.925	N/A
NM_005754.11.9	G3BP1	chr5	151179449	151180430	N/A	N/A	1	20.925	N/A
NM_001105515.19.15	ABCC4	chr13	95813442	95818621	N/A	N/A	1	20.925	N/A
NM_001195144.25.21	AC013264.1	chr2	197863060	197870634	N/A	N/A	1	20.925	N/A
NM_018416.2.2	FOXJ2	chr12	8192414	8192761	N/A	N/A	1	20.925	N/A
NM_001199398.11.6	NEK1	chr4	170501992	170511960	N/A	N/A	1	20.925	N/A
NM_001243835.9.7	STAT4	chr2	191927487	191931241	N/A	N/A	1	20.925	N/A
NM_019043.8.5	APBB1IP	chr10	26789747	26802589	N/A	N/A	1	20.925	N/A
NM_002035.9.2	KDSR	chr18	61002489	61030101	N/A	N/A	1	20.925	N/A
NM_004774.13.11	MED1	chr17	37579579	37580991	N/A	N/A	1	20.925	N/A
NM_007282.9.6	RNF13	chr3	149613259	149639014	N/A	N/A	1	20.925	N/A
NM_004941.16.10	DHX8	chr17	41576229	41585888	N/A	N/A	1	20.925	N/A
NM_018291.5.2	FGGY	chr1	59787207	59844509	N/A	N/A	1	20.925	N/A
NM_003105.32.24	SORL1	chr11	121444949	121466481	N/A	N/A	1	20.925	N/A
NM_014616.24.22	ATP11B	chr3	182602540	182605501	N/A	N/A	1	20.925	N/A
NM_015171.23.22	XPO6	chr16	28112778	28113266	N/A	N/A	1	20.925	N/A
NM_006098.4.2	RACK1	chr5	180666486	180669345	N/A	N/A	1	20.925	N/A
NM_001267727.2.2	ARSG	chr17	66303083	66303852	N/A	N/A	1	20.925	N/A
NM_015113.35.29	ZZEF1	chr17	3955264	3968123	N/A	N/A	1	20.925	N/A
NM_001206999.22.20	CIT	chr12	120195174	120198859	N/A	N/A	1	20.925	N/A
NM_001253699.7.5	ERBIN	chr5	65307876	65310553	N/A	N/A	1	20.925	N/A
NM_004724.11.6	ZW10	chr11	113612415	113629428	N/A	N/A	1	20.925	N/A
NM_020245.5.4	TULP4	chr6	158870027	158873300	N/A	N/A	1	20.925	N/A
NM_020165.7.3	RAD18	chr3	8977554	8990254	N/A	N/A	1	20.925	N/A
NM_198097.8.5	CCZ1B	chr7	6854394	6862991	N/A	N/A	1	20.925	N/A
NM_173545.8.2	APLF	chr2	68717321	68772444	N/A	N/A	1	20.925	N/A
NM_005829.3.2	AP3S2	chr15	90431752	90432372	N/A	N/A	1	20.925	N/A
NM_019004.10.6	ANKIB1	chr7	91972337	91991587	N/A	N/A	1	20.925	N/A

NR_026665.3.2	-	chr1	117078586	117087226	N/A	N/A	1	20.925	N/A
NM_001134415.7.3	TBCCD1	chr3	186268917	186276361	N/A	N/A	1	20.925	N/A
NM_004810.7.2	GRAP2	chr22	40343096	40365537	N/A	N/A	1	20.925	N/A
NM_024887.8.6	DHDDS	chr1	26774049	26786635	N/A	N/A	1	20.925	N/A
NM_018023.19.18	YEATS2	chr3	183493702	183495488	N/A	N/A	1	20.925	N/A
NM_138443.6.3	HAUS1	chr18	43698146	43703330	N/A	N/A	1	20.925	N/A
NM_004068.4.3	AP2M1	chr3	183896644	183898039	N/A	N/A	1	20.925	N/A
NM_001199261.7.4	UCHL5	chr1	192997214	192998787	N/A	N/A	1	20.925	N/A
NM_001243249.9.8	NPRL3	chr16	142593	143323	N/A	N/A	1	20.925	N/A
NM_018429.35.29	BDP1	chr5	70837280	70849147	N/A	N/A	1	20.925	N/A
NM_014223.6.2	NFYC	chr1	41204507	41223966	N/A	N/A	1	20.925	N/A
NM_001144884.8.3	SLC30A7	chr1	101372407	101387397	N/A	N/A	1	20.925	N/A
NM_006260.9.5	DNAJC3	chr13	96409897	96416207	N/A	N/A	1	20.925	N/A
NM_032295.7.6	SLC37A3	chr7	140055467	140058585	N/A	N/A	1	20.925	N/A
NM_015092.49.39	SMG1	chr16	18846213	18856973	N/A	N/A	1	20.925	N/A
NM_017757.6.4	ZNF407	chr18	72589152	72601994	N/A	N/A	1	20.925	N/A
NM_001204387.13.8	DLG1	chr3	196807921	196846401	N/A	N/A	1	20.925	N/A
NM_005505.7.2	SCARB1	chr12	125292306	125302253	N/A	N/A	1	20.925	N/A
NM_017990.9.7	PDPR	chr16	70164325	70166203	N/A	N/A	1	20.925	N/A
NM_006437.15.13	PARP4	chr13	25049609	25052414	N/A	N/A	1	20.925	N/A
NM_014802.14.10	C2CD5	chr12	22635490	22659753	N/A	N/A	1	20.925	N/A
NM_015020.13.6	PHLPP2	chr16	71697801	71715808	N/A	N/A	1	20.925	N/A
NM_001134938.10.8	FIP1L1	chr4	54265896	54294350	N/A	N/A	1	20.925	N/A
NM_001203246.9.6	GPBP1	chr5	56542126	56546968	N/A	N/A	1	20.925	N/A
NM_015531.22.16	C2CD3	chr11	73795925	73809300	N/A	N/A	1	20.925	N/A
NM_002519.12.9	NPAT	chr11	108046972	108056221	N/A	N/A	1	20.925	N/A
NM_001271.37.33	CHD2	chr15	93545406	93558139	N/A	N/A	1	20.925	N/A
NM_014972.12.8	TCF25	chr16	89961445	89967202	N/A	N/A	1	20.925	N/A
NM_014972.12.9	TCF25	chr16	89962397	89967202	N/A	N/A	1	20.925	N/A
NM_016551.9.6	TM7SF3	chr12	27132716	27143560	N/A	N/A	1	20.925	N/A
NM_001083953.29.29	THADA	chr2	43625109	43625278	N/A	N/A	1	20.925	N/A
NM_020751.18.14	COG6	chr13	40293372	40301685	N/A	N/A	1	20.925	N/A
NM_001206946.7.2	PICALM	chr11	85722072	85742653	N/A	N/A	1	20.925	N/A

NM_001134338.4.2	RNF24	chr20	3928883	3955047	N/A	N/A	1	20.925	N/A
NM_001012515.4.2	FECH	chr18	55238623	55247431	N/A	N/A	1	20.925	N/A
NM_005422.19.18	TECTA	chr11	121038762	121039634	N/A	N/A	1	20.925	N/A
NM_001198957.15.12	DCAF6	chr1	168007608	168014465	N/A	N/A	1	20.925	N/A
NM_001163286.5.3	EWSR1	chr22	29668368	29674205	N/A	N/A	1	20.925	N/A
NM_015331.15.11	NCSTN	chr1	160323907	160326544	N/A	N/A	1	20.925	N/A
NM_001081573.8.5	GAB3	chrX	153924191	153928334	N/A	N/A	1	20.925	N/A
NM_203346.24.21	HDLBP	chr2	242173234	242176184	N/A	N/A	1	20.925	N/A
NM_001204426.9.5	LIMK1	chr7	73520204	73523366	N/A	N/A	1	20.925	N/A
NM_015382.40.31	HECTD1	chr14	31574588	31583546	N/A	N/A	1	20.925	N/A
NR_026999.10.8	-	chr7	39822333	39829143	N/A	N/A	1	20.925	N/A
NM_016604.11.8	KDM3B	chr5	137726701	137735701	N/A	N/A	1	20.925	N/A
NM_005121.16.16	MED13	chr17	60059558	60060475	N/A	N/A	1	20.925	N/A
NM_015555.13.11	ZNF451	chr6	57017018	57025950	N/A	N/A	1	20.925	N/A
NM_007124.13.10	UTRN	chr6	144758700	144765518	N/A	N/A	1	20.925	N/A
NM_001605.14.8	AARS	chr16	70292882	70302282	N/A	N/A	1	20.925	N/A
NM_005124.11.3	NUP153	chr6	17661883	17676001	N/A	N/A	1	20.925	N/A
NM_173647.3.2	RNF149	chr2	101910475	101911643	N/A	N/A	1	20.925	N/A
NM_004800.9.5	TM9SF2	chr13	100188861	100193921	N/A	N/A	1	20.925	N/A
NM_033656.15.13	BRWD1	chr21	40641824	40646398	N/A	N/A	1	20.925	N/A
NM_198433.8.5	AURKA	chr20	54956488	54961589	N/A	N/A	1	20.925	N/A
NM_005761.9.6	PLXNC1	chr12	94613791	94621011	N/A	N/A	1	20.925	N/A
NM_001282560.10.7	EYA3	chr1	28330846	28343750	N/A	N/A	1	20.925	N/A
NM_006243.3.2	PPP2R5A	chr1	212502476	212506940	N/A	N/A	1	20.925	N/A
NM_012398.6.4	PIP5K1C	chr19	3656402	3661999	N/A	N/A	1	20.925	N/A
NM_001257990.7.2	FBXO7	chr22	32874967	32889268	N/A	N/A	1	20.925	N/A
NM_000181.5.2	GUSB	chr7	65441001	65445396	N/A	N/A	1	20.925	N/A
NR_036627.14.10	-	chr3	50144199	50147121	N/A	N/A	1	20.925	N/A
NM_024956.10.6	TMEM62	chr15	43440952	43452992	N/A	N/A	1	20.925	N/A
NM_016447.6.3	MPP6	chr7	24663284	24690331	N/A	N/A	1	20.925	N/A
NM_020246.7.5	SLC12A9	chr7	100454489	100456783	N/A	N/A	1	20.925	N/A
NM_016334.10.8	GPR89B	chr1	147438785	147442191	N/A	N/A	1	20.925	N/A
NM_005993.35.34	TBCD	chr17	80890533	80895236	N/A	N/A	1	20.925	N/A

NM_133645.9.8	MTO1	chr6	74190397	74191967	N/A	N/A	1	20.925	N/A
NM_001271.7.3	CHD2	chr15	93467550	93482948	N/A	N/A	1	20.925	N/A
NM_001097616.5.3	-	chr1	145897084	145900490	N/A	N/A	1	20.925	N/A
NM_001433.7.4	ERN1	chr17	62145549	62157094	N/A	N/A	1	20.925	N/A
NM_206921.5.3	CEP85L	chr6	118832460	118887479	N/A	N/A	1	20.925	N/A
NM_000520.7.2	HEXA	chr15	72642858	72648958	N/A	N/A	1	20.925	N/A
NR_024075.13.8	-	chr19	6960307	6974685	N/A	N/A	1	20.925	N/A
NM_017772.7.3	TBC1D22B	chr6	37247079	37254848	N/A	N/A	1	20.925	N/A
NM_001282604.6.3	PTGES3	chr12	57064058	57066849	N/A	N/A	1	20.925	N/A
NM_152564.30.24	VPS13B	chr8	100479641	100533238	N/A	N/A	1	20.925	N/A
NM_001170464.3.2	CDK17	chr12	96717725	96728643	N/A	N/A	1	20.925	N/A
NM_015030.3.2	FRYL	chr4	48686689	48712715	N/A	N/A	1	20.925	N/A
NM_000922.5.3	PDE3B	chr11	14807982	14825596	N/A	N/A	1	20.925	N/A
NM_152892.3.2	LRWD1	chr7	102106263	102106717	N/A	N/A	1	20.925	N/A
NM_001271594.7.2	SESN3	chr11	94910882	94926686	N/A	N/A	1	20.925	N/A
NM_001282752.12.10	PUM2	chr2	20478343	20483247	N/A	N/A	1	20.925	N/A
NM_015171.5.2	XPO6	chr16	28181070	28192352	N/A	N/A	1	20.925	N/A
NM_170606.31.27	KMT2C	chr7	151891093	151896544	N/A	N/A	1	20.925	N/A
NM_020201.4.2	NT5M	chr17	17209856	17248222	N/A	N/A	1	20.925	N/A
NM_000107.7.4	DDB2	chr11	47254364	47256963	N/A	N/A	1	20.925	N/A
NM_015164.6.4	PLEKHM2	chr1	16044387	16046415	N/A	N/A	1	20.925	N/A
NM_001289030.8.5	GALK2	chr15	49528047	49575915	N/A	N/A	1	20.925	N/A
NM_001278617.19.10	BUB1	chr2	111406810	111419418	N/A	N/A	1	20.925	N/A
NM_006934.7.5	SLC6A9	chr1	44467984	44468657	N/A	N/A	1	20.925	N/A
NM_003234.18.2	TFRC	chr3	195780288	195803993	N/A	N/A	1	20.925	N/A
NM_024821.6.2	CCDC134	chr22	42204878	42209826	N/A	N/A	1	20.925	N/A
NM_014547.7.4	TMOD3	chr15	52179785	52188723	N/A	N/A	1	20.925	N/A
NM_005156.6.3	PTBP3	chr9	115024714	115060196	N/A	N/A	1	20.925	N/A
NM_014451.17.14	BBS9	chr7	33397466	33427756	N/A	N/A	1	20.925	N/A
NM_001159643.3.1	MCTP2	chr15	94841429	94858866	N/A	N/A	1	20.925	N/A
NM_007269.17.12	STXBP3	chr1	109336203	109342927	N/A	N/A	1	20.925	N/A
NM_001171805.7.2	PAPD7	chr5	6737622	6746540	N/A	N/A	1	20.925	N/A
NM_017635.3.2	KMT5B	chr11	67953247	67957619	N/A	N/A	1	20.925	N/A

NM_001286457.10.7	USP7	chr16	9009110	9011013	N/A	N/A	1	20.925	N/A
NM_001135024.5.3	PDHX	chr11	34969052	34982065	N/A	N/A	1	20.925	N/A
NM_001744.7.2	CAMK4	chr5	110679721	110784901	N/A	N/A	1	20.925	N/A
NM_033121.12.7	ANKRD13A	chr12	110461850	110468563	N/A	N/A	1	20.925	N/A
NM_001271886.5.2	AAGAB	chr15	67524151	67529158	N/A	N/A	1	20.925	N/A
NM_002370.3.2	MAGOH	chr1	53699213	53701307	N/A	N/A	1	20.925	N/A
NM_004384.3.1	CSNK1G3	chr5	122881110	122893258	N/A	N/A	1	20.925	N/A
NM_006540.4.3	NCOA2	chr8	71126137	71128999	N/A	N/A	1	20.925	N/A
NM_175861.5.3	TMTC1	chr12	29904598	29911710	N/A	N/A	1	20.925	N/A
NM_032290.4.2	SLF1	chr5	93964515	93966448	N/A	N/A	1	20.925	N/A
NM_014574.14.11	STRN3	chr14	31371741	31380368	N/A	N/A	1	20.925	N/A
NM_004371.7.4	COPA	chr1	160295332	160305112	N/A	N/A	1	20.925	N/A
NM_001144772.16.13	NSMAF	chr8	59513843	59515945	N/A	N/A	1	20.925	N/A
NM_173582.12.10	PGM2L1	chr11	74053505	74054461	N/A	N/A	1	20.925	N/A
NM_016589.6.2	TIMMDC1	chr3	119219541	119236162	N/A	N/A	1	20.925	N/A
NM_003061.25.23	SLIT1	chr10	98790513	98794299	N/A	N/A	1	20.925	N/A
NM_138799.6.2	MBOAT2	chr2	9022640	9098771	N/A	N/A	1	20.925	N/A
NM_002830.15.12	PTPN4	chr2	120677644	120692534	N/A	N/A	1	20.925	N/A
NM_014184.4.3	CNIH4	chr1	224553580	224559125	N/A	N/A	1	20.925	N/A
NM_014960.9.7	ARSG	chr17	66364688	66381313	N/A	N/A	1	20.925	N/A
NM_014456.8.3	PDCD4	chr10	112640990	112650428	N/A	N/A	1	20.925	N/A
NM_001271769.22.12	AP3B1	chr5	77385216	77461496	N/A	N/A	1	20.925	N/A
NM_020234.5.3	DTWD1	chr15	49917309	49926991	N/A	N/A	1	20.925	N/A
NM_003400.4.3	XPO1	chr2	61749745	61753656	N/A	N/A	1	20.925	N/A
NM_032590.11.11	KDM2B	chr12	121947369	121947842	N/A	N/A	1	20.925	N/A
NM_001277140.6.2	ACTR3	chr2	114670748	114691963	N/A	N/A	1	20.925	N/A
NM_001109938.4.3	C6orf136	chr6_ssto_hap7	1951050	1951523	N/A	N/A	1	20.925	N/A
NM_001161726.12.11	PPP2R5C	chr14	102368055	102372866	N/A	N/A	1	20.925	N/A
NM_020210.8.5	SEMA4B	chr15	90763024	90764997	N/A	N/A	1	20.925	N/A
NM_001080475.3.2	PLEKHM3	chr2	208841374	208866681	N/A	N/A	1	20.925	N/A
NM_023072.9.7	ZSWIM4	chr19	13928029	13930306	N/A	N/A	1	20.925	N/A
NM_001113512.5.4	ARHGEF7	chr13	111870025	111885640	N/A	N/A	1	20.925	N/A
NM_014671.10.3	UBE3C	chr7	156961741	156979715	N/A	N/A	1	20.925	N/A

NR_037659.10.4	-	chr12	89853414	89866052	N/A	N/A	1	20.925	N/A
NM_015115.5.2	DCUN1D4	chr4	52729602	52744020	N/A	N/A	1	20.925	N/A
NM_019004.9.2	ANKIB1	chr7	91924202	91981956	N/A	N/A	1	20.925	N/A
NM_001145044.7.5	SLCO3A1	chr15	92663694	92671719	N/A	N/A	1	20.925	N/A
NM_001042683.29.26	SHPRH	chr6	146209155	146216113	N/A	N/A	1	20.925	N/A
NM_019004.9.6	ANKIB1	chr7	91972337	91981956	N/A	N/A	1	20.925	N/A
NM_015482.9.5	SLC22A23	chr6	3284085	3298452	N/A	N/A	1	20.925	N/A
NM_014247.7.4	RAPGEF2	chr4	160243498	160251650	N/A	N/A	1	20.925	N/A
NM_014423.13.12	AFF4	chr5	132227855	132228810	N/A	N/A	1	20.925	N/A
NM_030768.9.2	ILKAP	chr2	239090705	239103511	N/A	N/A	1	20.925	N/A
NM_001280539.3.3	RNF19A	chr8	101299728	101300495	N/A	N/A	1	20.925	N/A
NM_145726.9.2	TRAF3	chr14	103296670	103363738	N/A	N/A	1	20.925	N/A
NM_021627.16.4	SENP2	chr3	185316745	185344181	N/A	N/A	1	20.925	N/A
NM_001247996.13.9	ASAP1	chr8	131172109	131193126	N/A	N/A	1	20.925	N/A
NM_001265612.12.9	CNOT1	chr16	58614536	58617086	N/A	N/A	1	20.925	N/A
NM_018312.3.2	PPP6R3	chr11	68272612	68287119	N/A	N/A	1	20.925	N/A
NM_001037283.6.2	EIF3B	chr7	2400344	2404164	N/A	N/A	1	20.925	N/A
NM_021249.8.2	SNX6	chr14	35055435	35078948	N/A	N/A	1	20.925	N/A
NM_024763.4.2	WDR78	chr1	67356836	67371058	N/A	N/A	1	20.925	N/A
NM_001025160.12.11	ADGRE5	chr19	14513408	14515374	N/A	N/A	1	20.925	N/A
NM_001040451.5.3	RUFY1	chr5	178994461	179004137	N/A	N/A	1	20.925	N/A
NM_005637.9.4	SS18	chr18	23612362	23637706	N/A	N/A	1	20.925	N/A
NM_006303.3.2	AIMP2	chr7	6054776	6057676	N/A	N/A	1	20.925	N/A
NM_020410.24.15	ATP13A1	chr19	19756682	19764703	N/A	N/A	1	20.925	N/A
NM_001172746.18.14	SEC23B	chr20	18523662	18535034	N/A	N/A	1	20.925	N/A
NM_001007188.5.3	DEPDC5	chr22	32154531	32161046	N/A	N/A	1	20.925	N/A
NM_001243689.8.2	LETMD1	chr12	51442816	51451911	N/A	N/A	1	20.925	N/A
NM_001251922.6.3	RAP1B	chr12	69044179	69050997	N/A	N/A	1	20.925	N/A
NM_003400.23.18	XPO1	chr2	61709514	61715906	N/A	N/A	1	20.925	N/A
NM_015168.11.3	ZC3H4	chr19	47584769	47597865	N/A	N/A	1	20.925	N/A
NM_003907.7.6	EIF2B5	chr3	183857867	183858518	N/A	N/A	1	20.925	N/A
NM_001273.33.31	CHD4	chr12	6690209	6690980	N/A	N/A	1	20.925	N/A
NM_002372.12.5	MAN2A1	chr5	109091029	109125228	N/A	N/A	1	20.925	N/A

NM_020935.10.3	USP37	chr2	219394678	219425606	N/A	N/A	1	20.925	N/A
NM_005188.11.10	CBL	chr11	119155678	119156276	N/A	N/A	1	20.925	N/A
NM_018440.3.2	PAG1	chr8	81942230	81982405	N/A	N/A	1	20.925	N/A
NM_138353.6.4	DCAF15	chr19	14066723	14067334	N/A	N/A	1	20.925	N/A
NM_020820.29.17	PREX1	chr20	47258705	47274766	N/A	N/A	1	20.925	N/A
NR_073598.8.4	-	chr15	50303041	50339661	N/A	N/A	1	20.925	N/A
NM_001031847.14.9	CPT1A	chr11	68540732	68560870	N/A	N/A	1	20.925	N/A
NM_020754.11.4	ARHGAP31	chr3	119099750	119128623	N/A	N/A	1	20.925	N/A
NM_015040.7.2	PIKFYVE	chr2	209136234	209153542	N/A	N/A	1	20.925	N/A
NM_052937.2.2	PCMTD1	chr8	52773404	52773806	N/A	N/A	1	20.925	N/A
NM_020120.3.2	UGGT1	chr2	128855002	128861588	N/A	N/A	1	20.925	N/A
NM_014716.10.6	ACAP1	chr17	7246697	7247967	N/A	N/A	1	20.925	N/A
NM_006322.19.12	TUBGCP3	chr13	113158345	113181798	N/A	N/A	1	20.925	N/A
NM_015288.4.2	JADE2	chr5	133871547	133887899	N/A	N/A	1	20.925	N/A
NM_004996.7.5	ABCC1	chr16	16110352	16130460	N/A	N/A	1	20.925	N/A
NM_014629.11.8	ARHGEF10	chr8	1824736	1833873	N/A	N/A	1	20.925	N/A
NM_001287054.5.2	ZKSCAN1	chr7	99616853	99621930	N/A	N/A	1	20.925	N/A
NR_036464.10.2	-	chr4	15626874	15646331	N/A	N/A	1	20.925	N/A
NM_001286792.6.5	SPATA13	chr13	24823614	24826000	N/A	N/A	1	20.925	N/A
NM_017774.9.8	CDKAL1	chr6	20781375	20846409	N/A	N/A	1	20.925	N/A
NM_017774.9.6	CDKAL1	chr6	20739749	20846409	N/A	N/A	1	20.925	N/A
NM_007368.22.19	RASA3	chr13	114757960	114766408	N/A	N/A	1	20.925	N/A
NM_001286277.17.12	TUBGCP3	chr13	113170753	113181798	N/A	N/A	1	20.925	N/A
NM_001287060.9.9	RABGEF1	chr7	66270126	66270383	N/A	N/A	1	20.925	N/A
NM_001253697.4.2	ERBIN	chr5	65284462	65290692	N/A	N/A	1	20.925	N/A
NM_033657.6.4	DAP3	chr1	155691307	155695810	N/A	N/A	1	20.925	N/A
NM_033657.6.5	DAP3	chr1	155695172	155695810	N/A	N/A	1	20.925	N/A
NM_001284226.13.7	ELP3	chr8	27987018	28019595	N/A	N/A	1	20.925	N/A
NM_020830.10.7	WDFY1	chr2	224746658	224760347	N/A	N/A	1	20.925	N/A
NM_003411.3.2	ZFY	chrY	2821949	2829687	N/A	N/A	1	20.925	N/A
NM_001605.9.8	AARS	chr16	70301561	70302282	N/A	N/A	1	20.925	N/A
NM_181701.4.2	QSOX2	chr9	139115852	139118720	N/A	N/A	1	20.925	N/A
NR_024019.5.3	-	chr5	176963358	176966148	N/A	N/A	1	20.925	N/A

NM_032213.6.5	ELMOD3	chr2	85595808	85596953	N/A	N/A	1	20.925	N/A
NM_001243137.18.14	PDE8A	chr15	85657103	85664245	N/A	N/A	1	20.925	N/A
NM_000918.4.3	P4HB	chr17	79813017	79813462	N/A	N/A	1	20.925	N/A
NM_173630.39.35	RTTN	chr18	67718646	67733158	N/A	N/A	1	20.925	N/A
NM_003184.22.19	TAF2	chr8	120768245	120774848	N/A	N/A	1	20.925	N/A
NM_014801.15.13	PCNX2	chr1	233334684	233344435	N/A	N/A	1	20.925	N/A
NM_152233.6.3	SNX6	chr14	35072553	35078948	N/A	N/A	1	20.925	N/A
NM_015295.28.25	SMCHD1	chr18	2732262	2740819	N/A	N/A	1	20.925	N/A
NM_001003652.6.3	SMAD2	chr18	45391429	45396935	N/A	N/A	1	20.925	N/A
NM_012096.10.7	APPL1	chr3	57276883	57282379	N/A	N/A	1	20.925	N/A
NM_001008938.16.14	CKAP5	chr11	46810230	46812133	N/A	N/A	1	20.925	N/A
NM_014868.3.2	RNF10	chr12	120984207	120990501	N/A	N/A	1	20.925	N/A
NM_017653.8.4	DYM	chr18	46858233	46906128	N/A	N/A	1	20.925	N/A
NM_014810.17.16	CEP350	chr1	180002996	180006224	N/A	N/A	1	20.925	N/A
NM_005601.3.2	NKG7	chr19	51875193	51875561	N/A	N/A	1	20.925	N/A
NM_002631.6.5	PGD	chr1	10464217	10468197	N/A	N/A	1	20.925	N/A
NM_032236.15.13	USP48	chr1	22041881	22048257	N/A	N/A	1	20.925	N/A
NM_024541.23.16	C10orf76	chr10	103699619	103755112	N/A	N/A	1	20.925	N/A
NM_007159.5.3	SLMAP	chr3	57827025	57835543	N/A	N/A	1	20.925	N/A
NM_017679.19.17	BCAS3	chr17	59112026	59118253	N/A	N/A	1	20.925	N/A
NM_001256182.3.2	ANKRD11	chr16	89484691	89497734	N/A	N/A	1	20.925	N/A
NM_016287.9.5	HP1BP3	chr1	21083658	21100103	N/A	N/A	1	20.925	N/A
NM_015015.5.2	KDM4B	chr19	5016267	5041262	N/A	N/A	1	20.925	N/A
NM_020428.20.17	SLC44A2	chr19	10748319	10748991	N/A	N/A	1	20.925	N/A
NM_145297.3.2	ZNF626	chr19	20828489	20829211	N/A	N/A	1	20.925	N/A
NM_001257191.2.2	DDX6	chr11	118656760	118657227	N/A	N/A	1	20.925	N/A
NM_138572.8.6	TAF8	chr6	42034049	42044977	N/A	N/A	1	20.925	N/A
NM_021729.5.4	VPS11	chr11	118940943	118942553	N/A	N/A	1	20.925	N/A
NR_103528.13.10	-	chr13	49742748	49749677	N/A	N/A	1	20.925	N/A
NM_031915.2.2	SETDB2	chr13	50025688	50026045	N/A	N/A	1	20.925	N/A
NM_001784.16.11	ADGRE5	chr19	14515194	14518041	N/A	N/A	1	20.925	N/A
NM_001018039.7.5	SFMBT2	chr10	7318853	7327916	N/A	N/A	1	20.925	N/A
NM_207113.7.4	SLC37A3	chr7	140055467	140069482	N/A	N/A	1	20.925	N/A

NM_001005207.24.20	TRIM37	chr17	57076741	57094785	N/A	N/A	1	20.925	N/A
NM_001270978.5.2	IST1	chr16	71954641	71957283	N/A	N/A	1	20.925	N/A
NM_017925.10.4	DENND4C	chr9	19288593	19305525	N/A	N/A	1	20.925	N/A
NM_002285.23.19	AFF3	chr2	100170772	100182085	N/A	N/A	1	20.925	N/A
NM_001136473.3.2	LITAF	chr16	11647388	11650591	N/A	N/A	1	20.925	N/A
NM_001893.8.3	CSNK1D	chr17	80206750	80213453	N/A	N/A	1	20.925	N/A
NM_014949.10.7	KIAA0907	chr1	155891165	155895634	N/A	N/A	1	20.925	N/A
NM_001278533.11.9	ERMARD	chr6	170162524	170168267	N/A	N/A	1	20.925	N/A
NM_020193.14.9	EMSY	chr11	76207258	76239510	N/A	N/A	1	20.925	N/A
NM_004446.16.14	EPRS	chr1	220178589	220180680	N/A	N/A	1	20.925	N/A
NM_001243879.5.3	TRA2B	chr3	185638891	185641772	N/A	N/A	1	20.925	N/A
NM_001199942.4.3	DOPEY1	chr6	83806643	83810605	N/A	N/A	1	20.925	N/A
NM_175854.6.5	PAN3	chr13	28771321	28794515	N/A	N/A	1	20.925	N/A
NM_006321.13.12	ARIH2	chr3	49016914	49017929	N/A	N/A	1	20.925	N/A
NM_033116.10.9	NEK9	chr14	75576387	75577074	N/A	N/A	1	20.925	N/A
NM_004380.20.17	CREBBP	chr16	3801726	3808973	N/A	N/A	1	20.925	N/A
NM_020823.6.4	TMEM181	chr6	159004985	159010814	N/A	N/A	1	20.925	N/A
NM_001286724.4.2	FAM120A	chr9	96233422	96259881	N/A	N/A	1	20.925	N/A
NM_170606.15.12	KMT2C	chr7	151935791	151948051	N/A	N/A	1	20.925	N/A
NM_199141.4.2	CARM1	chr19	11015626	11019883	N/A	N/A	1	20.925	N/A
NM_000885.10.5	ITGA4	chr2	182343483	182350719	N/A	N/A	1	20.925	N/A
NR_102436.3.2	-	chr1	235993525	235996967	N/A	N/A	1	20.925	N/A
NM_000110.8.4	DPYD	chr1	98144650	98206035	N/A	N/A	1	20.925	N/A
NM_001202449.5.4	ADK	chr10	76153898	76158337	N/A	N/A	1	20.925	N/A
NM_003870.35.32	IQGAP1	chr15	91030185	91035943	N/A	N/A	1	20.925	N/A
NM_001031801.4.2	LIMK2	chr22	31654276	31656063	N/A	N/A	1	20.925	N/A
NM_153029.4.2	N4BP1	chr16	48585296	48596355	N/A	N/A	1	20.925	N/A
NM_003901.9.6	SGPL1	chr10	72617370	72629654	N/A	N/A	1	20.925	N/A
NM_013300.6.3	FAM216A	chr12	110922882	110925748	N/A	N/A	1	20.925	N/A
NM_175854.15.6	PAN3	chr13	28794367	28851513	N/A	N/A	1	20.925	N/A
NM_001207044.6.2	CLASP2	chr3	33661094	33686395	N/A	N/A	1	20.925	N/A
NM_001114380.18.14	ITGAL	chr16	30510394	30516783	N/A	N/A	1	20.925	N/A
NM_003036.5.2	SKI	chr1	2234416	2236024	N/A	N/A	1	20.925	N/A

NM_000610.14.6	CD44	chr11	35218292	35232996	N/A	N/A	1	20.925	N/A
NR_073001.3.2	-	chr7	152480271	152498816	N/A	N/A	1	20.925	N/A
NM_194442.8.7	LBR	chr1	225600155	225603034	N/A	N/A	1	20.925	N/A
NM_020774.3.2	MIB1	chr18	19345732	19348713	N/A	N/A	1	20.925	N/A
NM_001012614.4.2	CTBP1	chr4	1221986	1235307	N/A	N/A	1	20.925	N/A
NM_001002814.5.2	RAB11FIP1	chr8	37727937	37735069	N/A	N/A	1	20.925	N/A
NM_001142446.5.4	ANK1	chr8	41584767	41585524	N/A	N/A	1	20.925	N/A
NM_145059.3.2	FUK	chr16	70497071	70497677	N/A	N/A	1	20.925	N/A
NM_004668.44.22	MGAM	chr7	141747584	141799537	N/A	N/A	1	20.925	N/A
NM_000528.14.11	MAN2B1	chr19	12766507	12768369	N/A	N/A	1	20.925	N/A
NR_026580.10.2	-	chr13	114240088	114292199	N/A	N/A	1	20.925	N/A
NM_024947.4.2	PHC3	chr3	169889160	169896726	N/A	N/A	1	20.925	N/A
NM_001077207.25.24	SEC31A	chr4	83745707	83748785	N/A	N/A	1	20.925	N/A
NM_199340.9.4	LRRC37A3	chr17	62864579	62888718	N/A	N/A	1	20.925	N/A
NM_005469.5.4	ACOT8	chr20	44472165	44473059	N/A	N/A	1	20.925	N/A
NM_002973.14.12	ATXN2	chr12	111947352	111948386	N/A	N/A	1	20.925	N/A
NM_032043.16.13	BRIP1	chr17	59820373	59857762	N/A	N/A	1	20.925	N/A
NM_001286644.13.5	RFWD2	chr1	175996707	176105683	N/A	N/A	1	20.925	N/A
NM_001256.10.3	CDC27	chr17	45221248	45249430	N/A	N/A	1	20.925	N/A
NM_005534.6.5	IFNGR2	chr21	34804483	34805178	N/A	N/A	1	20.925	N/A
NM_001114403.5.2	UPK3BL	chr7	102279160	102281220	N/A	N/A	1	20.925	N/A
NM_033125.7.2	SLC22A16	chr6	110752373	110778220	N/A	N/A	1	20.925	N/A
NR_024495.8.6	-	chr10	48194832	48197195	N/A	N/A	1	20.925	N/A
NM_170606.23.15	KMT2C	chr7	151917607	151935911	N/A	N/A	1	20.925	N/A
NM_001190242.7.3	IFT80	chr3	160073800	160095328	N/A	N/A	1	20.925	N/A
NM_001172671.4.3	ZNF430	chr19	21216261	21216990	N/A	N/A	1	20.925	N/A
NR_036680.9.7	-	chr7	32672154	32678977	N/A	N/A	1	20.925	N/A
NM_199443.8.4	USP4	chr3	49343147	49363278	N/A	N/A	1	20.925	N/A
NM_001242768.24.23	MTHFD1L	chr6	151336015	151336829	N/A	N/A	1	20.925	N/A
NM_018989.11.10	RBM27	chr5	145634505	145638156	N/A	N/A	1	20.925	N/A
NM_030816.7.4	ANKRD13C	chr1	70766446	70781249	N/A	N/A	1	20.925	N/A
NM_004990.15.8	MARS	chr12	57891939	57906747	N/A	N/A	1	20.925	N/A
NM_015189.6.2	EXOC6B	chr2	72945231	72968598	N/A	N/A	1	20.925	N/A

NM_001178035.11.8	CEP85L	chr6	118801033	118805021	N/A	N/A	1	20.925	N/A
NM_001014431.13.2	AKT1	chr14	105237081	105259059	N/A	N/A	1	20.925	N/A
NM_006048.6.4	UBE4B	chr1	10161165	10165802	N/A	N/A	1	20.925	N/A
NM_001012967.13.6	DDX60L	chr4	169351628	169379159	N/A	N/A	1	20.925	N/A
NM_178566.8.3	ZDHHC21	chr9	14639893	14680160	N/A	N/A	1	20.925	N/A
NM_001286577.7.6	C2CD3	chr11	73843888	73844602	N/A	N/A	1	20.925	N/A
NR_104214.7.4	-	chr1	28354299	28369161	N/A	N/A	1	20.925	N/A
NM_014933.4.2	SEC31A	chr4	83799882	83803090	N/A	N/A	1	20.925	N/A
NM_016028.2.2	KMT5B	chr11	67957383	67957619	N/A	N/A	1	20.925	N/A
NM_001114403.5.4	UPK3BL	chr7	102279160	102279646	N/A	N/A	1	20.925	N/A
NM_020823.10.7	TMEM181	chr6	159026298	159029782	N/A	N/A	1	20.925	N/A
NM_153607.4.4	CREBRF	chr5	172517317	172518404	N/A	N/A	1	20.925	N/A
NM_015447.4.2	CAMSAP1	chr9	138758301	138774924	N/A	N/A	1	20.925	N/A
NM_144604.16.14	ZC3H18	chr16	88694029	88695262	N/A	N/A	1	20.925	N/A
NM_001282129.8.6	SSH2	chr17	28003837	28011702	N/A	N/A	1	20.925	N/A
NM_002887.7.2	RARS	chr5	167915606	167924353	N/A	N/A	1	20.925	N/A
NM_007124.45.43	UTRN	chr6	144858717	144864006	N/A	N/A	1	20.925	N/A
NM_020245.6.3	TULP4	chr6	158850767	158882761	N/A	N/A	1	20.925	N/A
NM_001190964.22.20	ZMYM2	chr13	20638590	20641530	N/A	N/A	1	20.925	N/A
NM_004733.4.2	SLC33A1	chr3	155551256	155560408	N/A	N/A	1	20.925	N/A
NR_003369.6.2	-	chr16	29087976	29096597	N/A	N/A	1	20.925	N/A
NM_199141.8.6	CARM1	chr19	11024552	11027453	N/A	N/A	1	20.925	N/A
NM_001127184.3.2	CFLAR	chr2	201994451	201997857	N/A	N/A	1	20.925	N/A
NM_001271.17.13	CHD2	chr15	93492181	93510743	N/A	N/A	1	20.925	N/A
NM_015276.10.8	USP22	chr17	20910208	20914622	N/A	N/A	1	20.925	N/A
NM_016287.10.5	HP1BP3	chr1	21076215	21100103	N/A	N/A	1	20.925	N/A
NM_001145819.13.5	SOX6	chr11	16036487	16208501	N/A	N/A	1	20.925	N/A
NM_006750.3.2	SNTB2	chr16	69279504	69294163	N/A	N/A	1	20.925	N/A
NR_034053.5.3	-	chr7	128655032	128658211	N/A	N/A	1	20.925	N/A
NM_017918.4.2	CCDC109B	chr4	110580166	110585550	N/A	N/A	1	20.925	N/A
NM_003298.5.4	NR2C2	chr3	15055095	15057761	N/A	N/A	1	20.925	N/A
NM_012486.8.6	PSEN2	chr1	227073238	227076750	N/A	N/A	1	20.925	N/A
NR_036627.13.12	-	chr3	50145502	50145737	N/A	N/A	1	20.925	N/A

NR_028040.5.2	-	chr7	99820219	99831297	N/A	N/A	1	20.925	N/A
NM_030938.5.2	VMP1	chr17	57808781	57816308	N/A	N/A	1	20.925	N/A
NM_001199282.34.31	LRBA	chr4	151719232	151738409	N/A	N/A	1	20.925	N/A
NM_001282539.6.4	GNB1	chr1	1735857	1747301	N/A	N/A	1	20.925	N/A
NM_001098794.6.2	FAM160A2	chr11	6243671	6245937	N/A	N/A	1	20.925	N/A
NM_032932.14.11	RAB11FIP4	chr17	29854859	29857487	N/A	N/A	1	20.925	N/A
NM_000887.14.9	ITGAX	chr16	31372383	31374695	N/A	N/A	1	20.925	N/A
NM_001284382.4.3	UBE2Q2	chr15	76161291	76165909	N/A	N/A	1	20.925	N/A
NM_017554.5.4	PARP14	chr3	122411147	122414509	N/A	N/A	1	20.925	N/A
NM_007373.3.2	SHOC2	chr10	112723882	112745523	N/A	N/A	1	20.925	N/A
NM_014972.13.8	TCF25	chr16	89961445	89970613	N/A	N/A	1	20.925	N/A
NM_014992.2.1	DAAM1	chr14	59730158	59758024	N/A	N/A	1	20.925	N/A
NM_175710.10.6	CR1L	chr1	207870847	207881608	N/A	N/A	1	20.925	N/A
NM_175710.10.5	CR1L	chr1	207867697	207881608	N/A	N/A	1	20.925	N/A
NM_001277126.5.2	NLRP12	chr19	54308533	54318242	N/A	N/A	1	20.925	N/A
NM_001130926.6.4	MEF2A	chr15	100211527	100215663	N/A	N/A	1	20.925	N/A
NM_000560.3.2	CD53	chr1	111434013	111435155	N/A	N/A	1	20.925	N/A
NM_133494.5.2	NEK7	chr1	198201682	198233365	N/A	N/A	1	20.925	N/A
NM_173514.11.9	SLC38A9	chr5	54945034	54952602	N/A	N/A	1	20.925	N/A
NM_005359.10.5	SMAD4	chr18	48581150	48593557	N/A	N/A	1	20.925	N/A
NM_001111112.4.2	UBE2K	chr4	39739039	39757359	N/A	N/A	1	20.925	N/A
NR_023361.4.2	-	chr15	90420537	90432372	N/A	N/A	1	20.925	N/A
NM_152718.14.12	VWCE	chr11	61039146	61042056	N/A	N/A	1	20.925	N/A
NM_005848.11.8	DENND4A	chr15	66021409	66025156	N/A	N/A	1	20.925	N/A
NM_020458.5.4	TTC7A	chr2	47202111	47206046	N/A	N/A	1	20.925	N/A
NM_015073.12.11	SIPA1L3	chr19	38631823	38633350	N/A	N/A	1	20.925	N/A
NM_012241.3.2	SIRT5	chr6	13579682	13584457	N/A	N/A	1	20.925	N/A
NR_002934.9.2	-	chr10	135269740	135280857	N/A	N/A	1	20.925	N/A
NM_024313.4.2	NOL12	chr22	38083916	38084999	N/A	N/A	1	20.925	N/A
NM_207116.15.14	RNF216	chr7	5680784	5692141	N/A	N/A	1	20.925	N/A
NM_003922.64.47	HERC1	chr15	63929703	63952135	N/A	N/A	1	20.925	N/A
NM_014606.10.4	HERC3	chr4	89570990	89579642	N/A	N/A	1	20.925	N/A
NM_001128602.11.10	RASGRP1	chr15	38794512	38798121	N/A	N/A	1	20.925	N/A

NM_001707.4.3	BCL7B	chr7	72954211	72957974	N/A	N/A	1	20.925	N/A
NM_005626.4.4	SRSF4	chr1	29481207	29481422	N/A	N/A	1	20.925	N/A
NM_152707.6.5	SLC25A16	chr10	70252888	70253327	N/A	N/A	1	20.925	N/A
NM_016316.9.7	REV1	chr2	100046301	100052403	N/A	N/A	1	20.925	N/A
NM_006662.4.4	SRCAP	chr16	30715384	30715636	N/A	N/A	1	20.925	N/A
NM_001243737.3.2	TRAPPC13	chr5	64931127	64931870	N/A	N/A	1	20.925	N/A
NM_015239.3.2	AGTPBP1	chr9	88307603	88327481	N/A	N/A	1	20.925	N/A
NM_007055.17.15	POLR3A	chr10	79761954	79767624	N/A	N/A	1	20.925	N/A
NM_153827.11.2	MINK1	chr17	4781611	4790474	N/A	N/A	1	20.925	N/A
NM_024105.7.4	ALG12	chr22	50301368	50304255	N/A	N/A	1	20.925	N/A
NM_018046.7.6	AGGF1	chr5	76342171	76344097	N/A	N/A	1	20.925	N/A
NM_000946.9.5	PRIM1	chr12	57135218	57139965	N/A	N/A	1	20.925	N/A
NM_001161454.6.5	CYB561A3	chr11	61120456	61121464	N/A	N/A	1	20.925	N/A
NM_000946.9.7	PRIM1	chr12	57135218	57136880	N/A	N/A	1	20.925	N/A
NM_001440.3.2	EXTL3	chr8	28570973	28575724	N/A	N/A	1	20.925	N/A
NM_001160167.3.2	PRR5L	chr11	36422546	36424928	N/A	N/A	1	20.925	N/A
NM_024857.15.12	ATAD5	chr17	29195350	29203568	N/A	N/A	1	20.925	N/A
NM_001109662.7.5	HECTD4	chr12	112743867	112747473	N/A	N/A	1	20.925	N/A
NM_001109662.7.4	HECTD4	chr12	112743867	112750951	N/A	N/A	1	20.925	N/A
NM_017453.3.2	STAU1	chr20	47782533	47790806	N/A	N/A	1	20.925	N/A
NM_001278210.11.6	NUP153	chr6	17661883	17669777	N/A	N/A	1	20.925	N/A
NM_014611.64.54	MDN1	chr6	90400391	90411780	N/A	N/A	1	20.925	N/A
NM_001145354.7.3	MKLN1	chr7	131060182	131084192	N/A	N/A	1	20.925	N/A
NM_006468.5.3	POLR3C	chr1	145606274	145608659	N/A	N/A	1	20.925	N/A
NM_012327.13.2	PIGN	chr18	59805475	59830889	N/A	N/A	1	20.925	N/A
NM_016227.9.3	SUCO	chr1	172520651	172539870	N/A	N/A	1	20.925	N/A
NM_001286531.4.2	RAD9B	chr12	110941610	110944498	N/A	N/A	1	20.925	N/A
NM_002519.9.4	NPAT	chr11	108056129	108062917	N/A	N/A	1	20.925	N/A
NM_003342.5.3	UBE2G1	chr17	4186092	4200109	N/A	N/A	1	20.925	N/A
NM_001109662.54.42	HECTD4	chr12	112641439	112666615	N/A	N/A	1	20.925	N/A
NM_003342.5.2	UBE2G1	chr17	4186092	4210418	N/A	N/A	1	20.925	N/A
NR_027155.11.11	-	chr16	21478412	21478637	N/A	N/A	1	20.925	N/A
NR_046386.4.2	-	chr9	6420911	6460791	N/A	N/A	1	20.925	N/A

NM_015396.13.3	ARMC8	chr3	137928658	137964025	N/A	N/A	1	20.925	N/A
NM_016016.5.2	SLC25A39	chr17	42399786	42400945	N/A	N/A	1	20.925	N/A
NM_006386.8.2	DDX17	chr22	38890634	38897285	N/A	N/A	1	20.925	N/A
NM_024820.10.2	DENND1A	chr9	126392654	126641300	N/A	N/A	1	20.925	N/A
NM_004457.4.3	ACSL3	chr2	223765391	223773868	N/A	N/A	1	20.925	N/A
NM_002691.3.2	POLD1	chr19	50902107	50902741	N/A	N/A	1	20.925	N/A
NM_001128148.4.3	TFRC	chr3	195800800	195802231	N/A	N/A	1	20.925	N/A
NM_001205247.17.15	ACSL6	chr5	131302108	131305893	N/A	N/A	1	20.925	N/A
NM_054027.8.3	ANKH	chr5	14741935	14758707	N/A	N/A	1	20.925	N/A
NM_001256126.8.5	CERS6	chr2	169547543	169574488	N/A	N/A	1	20.925	N/A
NM_001039650.4.3	ZMYM5	chr13	20425494	20426330	N/A	N/A	1	20.925	N/A
NM_001102.11.10	ACTN1	chr14	69356855	69359000	N/A	N/A	1	20.925	N/A
NM_198450.8.6	APOOL	chrX	84322132	84329397	N/A	N/A	1	20.925	N/A
NM_007331.5.4	WHSC1	chr4	1902352	1906105	N/A	N/A	1	20.925	N/A
NR_024386.10.7	-	chr17	62782684	62793527	N/A	N/A	1	20.925	N/A
NM_020536.6.4	KAT14	chr20	18139727	18143589	N/A	N/A	1	20.925	N/A
NM_015132.9.2	SNX13	chr7	17908029	17937069	N/A	N/A	1	20.925	N/A
NM_015185.6.3	ARHGEF9	chrX	62893917	62926329	N/A	N/A	1	20.925	N/A
NM_005465.8.1	AKT3	chr1	243736227	244006584	N/A	N/A	1	20.925	N/A
NM_005990.16.15	STK10	chr5	171482591	171484477	N/A	N/A	1	20.925	N/A
NM_017880.9.3	C2orf42	chr2	70387756	70409129	N/A	N/A	1	20.925	N/A
NM_001190214.3.2	PPIP5K1	chr15	43876555	43877090	N/A	N/A	1	20.925	N/A
NM_014756.5.2	CKAP5	chr11	46832556	46842819	N/A	N/A	1	20.925	N/A
NM_002647.21.14	PIK3C3	chr18	39607406	39629569	N/A	N/A	1	20.925	N/A
NM_002830.16.13	PTPN4	chr2	120684173	120702816	N/A	N/A	1	20.925	N/A
NM_001114380.11.10	ITGAL	chr16	30505532	30506171	N/A	N/A	1	20.925	N/A
NM_001488.6.2	TADA2A	chr17	35771355	35800763	N/A	N/A	1	20.925	N/A
NM_153050.5.3	MTMR3	chr22	30366964	30375041	N/A	N/A	1	20.925	N/A
NM_002498.10.4	NEK3	chr13	52718050	52728127	N/A	N/A	1	20.925	N/A
NM_001105214.9.4	ASH2L	chr8	37967896	37976881	N/A	N/A	1	20.925	N/A
NM_005688.5.2	ABCC5	chr3	183705557	183732235	N/A	N/A	1	20.925	N/A
NM_001009814.17.15	VWA8	chr13	42385360	42393522	N/A	N/A	1	20.925	N/A
NM_001134937.12.11	FIP1L1	chr4	54292038	54294350	N/A	N/A	1	20.925	N/A

NM_001127660.3.2	MFN2	chr1	12049221	12052747	N/A	N/A	1	20.925	N/A
NM_014957.7.6	DENND3	chr8	142160932	142161936	N/A	N/A	1	20.925	N/A
NM_022366.4.3	TFB2M	chr1	246719874	246720836	N/A	N/A	1	20.925	N/A
NM_152279.4.2	ZNF585B	chr19	37680562	37698156	N/A	N/A	1	20.925	N/A
NM_018489.7.4	ASH1L	chr1	155365249	155429689	N/A	N/A	1	20.925	N/A
NM_015060.12.2	AVL9	chr7	32582752	32613030	N/A	N/A	1	20.925	N/A
NR_037193.7.2	-	chr11	62650379	62653080	N/A	N/A	1	20.925	N/A
NM_001144912.11.7	ATG7	chr3	11372813	11389509	N/A	N/A	1	20.925	N/A
NM_017437.9.2	CPSF2	chr14	92592465	92609638	N/A	N/A	1	20.925	N/A
NM_018230.9.6	NUP133	chr1	229625701	229634053	N/A	N/A	1	20.925	N/A
NM_001280539.4.3	RNF19A	chr8	101287180	101300495	N/A	N/A	1	20.925	N/A
NR_048537.9.6	-	chr20	48466115	48481381	N/A	N/A	1	20.925	N/A
NM_018126.4.3	TMEM33	chr4	41941212	41945831	N/A	N/A	1	20.925	N/A
NM_003972.11.6	BTAF1	chr10	93713493	93719911	N/A	N/A	1	20.925	N/A
NM_032012.8.2	TMEM245	chr9	111843118	111870850	N/A	N/A	1	20.925	N/A
NM_002372.20.18	MAN2A1	chr5	109181565	109191035	N/A	N/A	1	20.925	N/A
NM_018318.7.3	CCDC91	chr12	28458581	28544344	N/A	N/A	1	20.925	N/A
NM_003286.13.9	TOP1	chr20	39721111	39729993	N/A	N/A	1	20.925	N/A
NM_015914.11.7	TXNDC11	chr16	11778020	11792181	N/A	N/A	1	20.925	N/A
NM_024117.9.8	MAPKAP1	chr9	128230250	128246862	N/A	N/A	1	20.925	N/A
NM_001288999.7.5	GMIP	chr19	19750893	19751380	N/A	N/A	1	20.925	N/A
NM_006519.4.3	DYNLT1	chr6	159058159	159058926	N/A	N/A	1	20.925	N/A
NM_006519.4.2	DYNLT1	chr6	159058159	159062392	N/A	N/A	1	20.925	N/A
NM_016824.14.10	ADD3	chr10	111883774	111892158	N/A	N/A	1	20.925	N/A
NM_032206.23.15	NLRC5	chr16	57073692	57085533	N/A	N/A	1	20.925	N/A
NM_145206.5.2	VTI1A	chr10	114220282	114298089	N/A	N/A	1	20.925	N/A
NM_194282.5.3	LIN54	chr4	83867410	83900159	N/A	N/A	1	20.925	N/A
NM_001116.8.7	ADCY9	chr16	4029116	4033441	N/A	N/A	1	20.925	N/A
NM_001252078.8.7	USP15	chr12	62743001	62749256	N/A	N/A	1	20.925	N/A
NM_001114380.11.3	ITGAL	chr16	30486626	30506171	N/A	N/A	1	20.925	N/A
NM_014709.44.33	USP34	chr2	61486845	61520685	N/A	N/A	1	20.925	N/A
NM_130438.2.1	DYRK1A	chr21	38792600	38845182	N/A	N/A	2	41.85	N/A
NM_001130065.26.23	MYO9B	chr19	17308600	17311638	N/A	N/A	2	41.85	N/A

NM_017607.6.4	PPP1R12C	chr19	55610151	55614936	N/A	N/A	2	41.85	N/A
NM_001746.10.7	CANX	chr5	179136873	179147561	N/A	N/A	2	41.85	N/A
NM_001031623.7.4	ZNF451	chr6	56989531	56999668	N/A	N/A	2	41.85	N/A
NM_001198665.20.12	ARHGEF12	chr11	120310837	120319923	N/A	N/A	2	41.85	N/A
NM_006421.19.15	ARFGEF1	chr8	68163533	68172161	N/A	N/A	2	41.85	N/A
NM_003922.26.22	HERC1	chr15	63990926	64008672	N/A	N/A	2	41.85	N/A
NM_182641.17.16	BPTF	chr17	65924470	65925603	N/A	N/A	2	41.85	N/A
NM_002605.15.14	PDE8A	chr15	85657103	85658718	N/A	N/A	2	41.85	N/A
NM_003576.5.2	STK24	chr13	99127074	99171727	N/A	N/A	2	41.85	N/A
NM_001270660.21.20	38777	chr5	10415599	10417516	N/A	N/A	2	41.85	N/A
NM_018691.4.2	FAM114A2	chr5	153413350	153414527	N/A	N/A	2	41.85	N/A
NM_032142.21.2	CEP192	chr18	12999419	13059311	N/A	N/A	2	41.85	N/A
NM_138570.10.3	SLC38A10	chr17	79244717	79258695	N/A	N/A	2	41.85	N/A
NM_018202.4.3	TMEM57	chr1	25775298	25780873	N/A	N/A	2	41.85	N/A
NR_024448.10.8	-	chr22	24025911	24037704	N/A	N/A	2	41.85	N/A
NM_173163.9.8	NFATC3	chr16	68217142	68225678	N/A	N/A	2	41.85	N/A
NM_024947.11.9	PHC3	chr3	169831147	169840532	N/A	N/A	2	41.85	N/A
NM_015172.32.27	PRRC2C	chr1	171548492	171556890	N/A	N/A	2	41.85	N/A
NM_001242539.13.11	NCOA6	chr20	33315062	33324562	N/A	N/A	2	41.85	N/A
NM_001146037.7.6	SLC14A1	chr18	43319127	43319627	N/A	N/A	2	41.85	N/A
NM_017921.5.3	NPLOC4	chr17	79577235	79589304	N/A	N/A	2	41.85	N/A
NM_001271.38.35	CHD2	chr15	93552374	93563488	N/A	N/A	2	41.85	N/A
NM_001738.3.2	CA1	chr8	86253827	86264013	N/A	N/A	2	41.85	N/A
NM_017491.5.3	WDR1	chr4	10099334	10105610	N/A	N/A	2	41.85	N/A
NM_006586.3.2	CNPY3	chr6	42902212	42903408	N/A	N/A	2	41.85	N/A
NM_053067.3.2	UBQLN1	chr9	86297865	86301070	N/A	N/A	2	41.85	N/A
NM_145243.8.3	OMA1	chr1	58971731	59002413	N/A	N/A	2	41.85	N/A
NM_016567.7.2	BCCIP	chr10	127515159	127529899	N/A	N/A	2	41.85	N/A
NM_001042734.2.2	SEC24B	chr4	110384056	110384800	N/A	N/A	2	41.85	N/A
NR_002212.3.2	-	chr12	93788384	93789309	N/A	N/A	2	41.85	N/A
NM_003184.14.7	TAF2	chr8	120800577	120810086	N/A	N/A	2	41.85	N/A
NM_006267.23.21	RANBP2	chr2	109388156	109389502	N/A	N/A	2	41.85	N/A
NM_022743.8.6	SMYD3	chr1	246078831	246093239	N/A	N/A	2	41.85	N/A

NM_014676.7.6	PUM1	chr1	31465236	31468067	N/A	N/A	2	41.85	N/A
NM_025160.12.8	WDR26	chr1	224585798	224592272	N/A	N/A	2	41.85	N/A
NM_012335.8.5	MYO1F	chr19	8616623	8618321	N/A	N/A	2	41.85	N/A
NM_016551.7.6	TM7SF3	chr12	27135705	27143560	N/A	N/A	2	41.85	N/A
NM_139126.9.8	PPIL4	chr6	149846278	149847912	N/A	N/A	2	41.85	N/A
NM_005359.8.6	SMAD4	chr18	48584494	48586286	N/A	N/A	2	41.85	N/A
NM_004819.3.2	SYMPK	chr19	46357432	46357765	N/A	N/A	2	41.85	N/A
NM_001429.9.7	EP300	chr22	41531816	41536261	N/A	N/A	2	41.85	N/A
NM_015024.9.8	XPO7	chr8	21835280	21837714	N/A	N/A	2	41.85	N/A
NM_006642.9.6	SDCCAG8	chr1	243456392	243480195	N/A	N/A	2	41.85	N/A
NM_019004.3.2	ANKIB1	chr7	91924202	91936970	N/A	N/A	2	41.85	N/A
NM_001190879.5.2	ORC4	chr2	148730307	148739650	N/A	N/A	2	41.85	N/A
NM_198081.3.2	SCML4	chr6	108070887	108093590	N/A	N/A	2	41.85	N/A
NM_001130148.8.6	CCDC77	chr12	541031	547720	N/A	N/A	2	41.85	N/A
NM_174917.4.2	ACSF3	chr16	89164998	89169167	N/A	N/A	2	41.85	N/A
NM_017546.3.2	CNOT11	chr2	101874252	101879153	N/A	N/A	2	41.85	N/A
NM_001009999.4.2	KDM1A	chr1	23356961	23377013	N/A	N/A	2	41.85	N/A
NM_020784.6.2	TXNDC16	chr14	53003436	53011089	N/A	N/A	2	41.85	N/A
NM_022773.4.2	LMF1	chr16	960930	1004666	N/A	N/A	2	41.85	N/A
NM_198889.28.28	ANKRD17	chr4	73956383	73958017	N/A	N/A	2	41.85	N/A
NM_016589.4.2	TIMMDC1	chr3	119219541	119222868	N/A	N/A	2	41.85	N/A
NM_005085.32.31	NUP214	chr9	134090598	134098317	N/A	N/A	2	41.85	N/A
NM_001131007.32.30	KIAA0922	chr4	154547298	154553990	N/A	N/A	2	41.85	N/A
NR_110283.3.2	-	chr2	208102543	208105062	N/A	N/A	2	41.85	N/A
NM_001083617.5.2	RB1CC1	chr8	53596108	53599527	N/A	N/A	2	41.85	N/A
NM_133433.9.2	NIPBL	chr5	36953719	36976504	N/A	N/A	2	41.85	N/A
NM_020476.31.30	ANK1	chr8	41550165	41550719	N/A	N/A	2	41.85	N/A
NM_002149.3.3	HPCAL1	chr2	10559859	10560261	N/A	N/A	2	41.85	N/A
NM_005339.6.2	UBE2K	chr4	39739039	39779430	N/A	N/A	2	41.85	N/A
NM_001080432.7.5	FTO	chr16	53907697	53922863	N/A	N/A	2	41.85	N/A
NM_025137.36.34	SPG11	chr15	44859621	44862856	N/A	N/A	2	41.85	N/A
NM_139132.8.5	NUP98	chr11	3789810	3797251	N/A	N/A	2	41.85	N/A
NM_007110.7.3	TEP1	chr14	20871535	20874559	N/A	N/A	2	41.85	N/A

NM_014957.10.9	DENND3	chr8	142170730	142173528	N/A	N/A	2	41.85	N/A
NM_003045.5.2	SLC7A1	chr13	30104674	30127997	N/A	N/A	2	41.85	N/A
NM_145080.5.3	NSMCE1	chr16	27244321	27246620	N/A	N/A	2	41.85	N/A
NM_001110303.23.19	USP20	chr9	132637107	132640719	N/A	N/A	2	41.85	N/A
NM_001282681.6.2	GAPVD1	chr9	128064261	128074891	N/A	N/A	2	41.85	N/A
NM_000126.9.2	ETFA	chr15	76566752	76588078	N/A	N/A	2	41.85	N/A
NM_016293.10.9	BIN2	chr12	51685374	51689662	N/A	N/A	2	41.85	N/A
NR_073070.5.3	-	chr4	56277780	56284152	N/A	N/A	2	41.85	N/A
NM_001145666.22.19	GLG1	chr16	74493579	74497377	N/A	N/A	2	41.85	N/A
NM_014869.3.2	IQSEC1	chr3	12976947	12983365	N/A	N/A	2	41.85	N/A
NM_206921.3.3	CEP85L	chr6	118886691	118887479	N/A	N/A	2	41.85	N/A
NM_017550.3.2	MIER2	chr19	334399	336173	N/A	N/A	2	41.85	N/A
NM_001142640.12.11	TNRC6C	chr17	76075475	76079247	N/A	N/A	2	41.85	N/A
NM_005197.2.2	FOXN3	chr14	89878277	89878834	N/A	N/A	2	41.85	N/A
NM_012235.8.4	SCAP	chr3	47466974	47470160	N/A	N/A	2	41.85	N/A
NR_003655.7.5	-	chr7	44026199	44027846	N/A	N/A	2	41.85	N/A
NM_007011.9.6	ABHD2	chr15	89656955	89698765	N/A	N/A	2	41.85	N/A
NM_013236.9.7	ATXN10	chr22	46125304	46136418	N/A	N/A	2	41.85	N/A
NM_018441.7.4	PECR	chr2	216908626	216923699	N/A	N/A	2	41.85	N/A
NM_001167608.7.4	RHBDD1	chr2	227729319	227773594	N/A	N/A	2	41.85	N/A
NM_030650.5.2	KIAA1715	chr2	176844537	176860374	N/A	N/A	2	41.85	N/A
NM_001242906.14.5	ASCC2	chr22	30196979	30212062	N/A	N/A	2	41.85	N/A
NM_016396.7.6	CTDSPL2	chr15	44788577	44789336	N/A	N/A	2	41.85	N/A
NM_182540.9.3	INTS6L	chrX	134679347	134690225	N/A	N/A	2	41.85	N/A
NM_018230.21.19	NUP133	chr1	229593907	229599423	N/A	N/A	2	41.85	N/A
NM_130807.4.3	MOB3A	chr19	2076809	2078678	N/A	N/A	2	41.85	N/A
NM_001097615.8.5	POLR2J3	chr7	102180052	102182109	N/A	N/A	2	41.85	N/A
NM_001278610.7.7	MYBL2	chr20	42331129	42331543	N/A	N/A	2	41.85	N/A
NM_001127715.4.2	STXBP5	chr6	147527106	147560406	N/A	N/A	2	41.85	N/A
NM_001042573.3.2	ENGASE	chr17	77073511	77073946	N/A	N/A	2	41.85	N/A
NM_001105562.6.3	UBE4B	chr1	10155518	10165802	N/A	N/A	2	41.85	N/A
NM_153341.3.2	RNF19B	chr1	33413822	33415375	N/A	N/A	2	41.85	N/A
NM_014868.14.13	RNF10	chr12	121004627	121009094	N/A	N/A	2	41.85	N/A

NM_018124.2.2	RFWD3	chr16	74694829	74695349	N/A	N/A	2	41.85	N/A
NM_015355.8.4	SUZ12	chr17	30274635	30303633	N/A	N/A	2	41.85	N/A
NM_020477.20.16	ANK1	chr8	41561558	41571772	N/A	N/A	2	41.85	N/A
NM_001134338.5.2	RNF24	chr20	3925823	3955047	N/A	N/A	2	41.85	N/A
NM_018023.20.20	YEATS2	chr3	183503912	183504089	N/A	N/A	2	41.85	N/A
NM_001145819.6.4	SOX6	chr11	16205431	16256217	N/A	N/A	2	41.85	N/A
NM_145648.5.2	SLC15A4	chr12	129293332	129299615	N/A	N/A	2	41.85	N/A
NM_001278210.13.6	NUP153	chr6	17648037	17669777	N/A	N/A	2	41.85	N/A
NM_020773.3.2	TBC1D14	chr4	6925099	6969151	N/A	N/A	2	41.85	N/A
NM_001167971.5.4	TRMT2B	chrX	100296305	100297301	N/A	N/A	2	41.85	N/A
NM_001114091.8.5	CDC27	chr17	45232037	45235669	N/A	N/A	2	41.85	N/A
NM_153337.3.2	SNX20	chr16	50709680	50711446	N/A	N/A	2	41.85	N/A
NM_144600.3.2	FOPNL	chr16	15973660	15978062	N/A	N/A	2	41.85	N/A
NM_199340.7.5	LRRC37A3	chr17	62882203	62885870	N/A	N/A	2	41.85	N/A
NM_005819.7.4	STX6	chr1	180953812	180962561	N/A	N/A	2	41.85	N/A
NM_006838.7.4	METAP2	chr12	95879654	95889874	N/A	N/A	2	41.85	N/A
NM_017552.7.2	ATAD2B	chr2	24103508	24118840	N/A	N/A	2	41.85	N/A
NM_018489.5.5	ASH1L	chr1	155408117	155408859	N/A	N/A	2	41.85	N/A
NM_001494.5.2	GDI2	chr10	5827814	5842668	N/A	N/A	2	41.85	N/A
NM_014801.17.13	PCNX2	chr1	233313547	233344435	N/A	N/A	2	41.85	N/A
NM_001256183.4.3	ANKRD11	chr16	89371613	89383483	N/A	N/A	2	41.85	N/A
NM_206943.7.5	LTBP1	chr2	33359859	33413918	N/A	N/A	2	41.85	N/A
NM_001080975.13.9	REPS2	chrX	17080560	17095530	N/A	N/A	2	41.85	N/A
NM_003384.11.2	VRK1	chr14	97299803	97327072	N/A	N/A	2	41.85	N/A
NM_012425.7.4	RSU1	chr10	16794537	16806509	N/A	N/A	2	41.85	N/A
NM_012425.7.3	RSU1	chr10	16794537	16824083	N/A	N/A	2	41.85	N/A
NM_033419.3.2	PGAP3	chr17	37840849	37842272	N/A	N/A	2	41.85	N/A
NM_002874.9.2	RAD23B	chr9	110062421	110087285	N/A	N/A	2	41.85	N/A
NM_018850.14.13	ABCB4	chr7	87068982	87069718	N/A	N/A	2	41.85	N/A
NM_032156.8.2	CAPRIN2	chr12	30881581	30904067	N/A	N/A	2	41.85	N/A
NM_024649.11.9	BBS1	chr11	66288740	66291353	N/A	N/A	2	41.85	N/A
NM_206866.4.2	BACH1	chr21	30693541	30702014	N/A	N/A	2	41.85	N/A
NM_152778.12.7	MFSD8	chr4	128842678	128861152	N/A	N/A	2	41.85	N/A

NM_032341.8.5	DDI2	chr1	15964801	15978390	N/A	N/A	2	41.85	N/A
NM_025151.2.2	RAB11FIP1	chr8	37734626	37735069	N/A	N/A	2	41.85	N/A
NM_004996.22.21	ABCC1	chr16	16200594	16205439	N/A	N/A	2	41.85	N/A
NM_017645.9.2	HAUS6	chr9	19080476	19096767	N/A	N/A	2	41.85	N/A
NM_015239.8.2	AGTPBP1	chr9	88284399	88327481	N/A	N/A	2	41.85	N/A
NR_103492.20.16	-	chr2	233675953	233684695	N/A	N/A	2	41.85	N/A
NM_001288756.8.7	BABAM1	chr19	17387303	17387718	N/A	N/A	2	41.85	N/A
NM_152641.16.7	ARID2	chr12	46230371	46254732	N/A	N/A	2	41.85	N/A
NM_015578.6.4	LSM14A	chr19	34699833	34706566	N/A	N/A	2	41.85	N/A
NM_203343.10.9	EPB41	chr1	29362337	29365938	N/A	N/A	2	41.85	N/A
NM_001145808.5.3	ITGAM	chr16	31276715	31277468	N/A	N/A	2	41.85	N/A
NM_001257190.3.2	POM121	chr7	72361165	72361649	N/A	N/A	2	41.85	N/A
NM_017880.4.3	C2orf42	chr2	70406663	70409129	N/A	N/A	2	41.85	N/A
NM_198468.10.7	MMS22L	chr6	97702432	97717868	N/A	N/A	2	41.85	N/A
NM_080840.5.3	PTPRA	chr20	2928627	2945848	N/A	N/A	2	41.85	N/A
NM_018351.2.2	FGD6	chr12	95602618	95605043	N/A	N/A	2	41.85	N/A
NM_001130020.17.14	ATP6V0A1	chr17	40647643	40653322	N/A	N/A	2	41.85	N/A
NM_213655.25.23	WNK1	chr12	1003727	1006847	N/A	N/A	2	41.85	N/A
NM_001193431.11.8	PTPN22	chr1	114391161	114397671	N/A	N/A	2	41.85	N/A
NM_016166.9.3	PIAS1	chr15	68434283	68466230	N/A	N/A	2	41.85	N/A
NR_002824.7.3	-	chr15	23333848	23356220	N/A	N/A	2	41.85	N/A
NM_004481.3.2	GALNT2	chr1	230313963	230339036	N/A	N/A	2	41.85	N/A
NM_053051.13.13	CNTROB	chr17	7849045	7849304	N/A	N/A	2	41.85	N/A
NM_004459.9.6	BPTF	chr17	65882243	65890281	N/A	N/A	2	41.85	N/A
NM_018184.6.5	ARL8B	chr3	5215701	5216099	N/A	N/A	2	41.85	N/A
NM_001167671.5.4	LPP	chr3	188202379	188242575	N/A	N/A	2	41.85	N/A
NM_000081.48.46	LYST	chr1	235850248	235860572	N/A	N/A	2	41.85	N/A
NM_002664.8.5	PLEK	chr2	68613633	68621308	N/A	N/A	2	41.85	N/A
NM_000455.6.2	STK11	chr19	1218415	1221339	N/A	N/A	2	41.85	N/A
NM_016955.8.3	SEPSECS	chr4	25146395	25158596	N/A	N/A	2	41.85	N/A
NM_002025.3.3	AFF2	chrX	147743428	147744289	N/A	N/A	2	41.85	N/A
NM_175710.11.5	CR1L	chr1	207867697	207891036	N/A	N/A	2	41.85	N/A
NM_001278604.5.2	LARP1B	chr4	128995614	129003460	N/A	N/A	2	41.85	N/A

NM_014155.2.2	ZBTB44	chr11	130130750	130131824	N/A	N/A	2	41.85	N/A
NM_015285.16.15	WDR7	chr18	54423813	54426184	N/A	N/A	2	41.85	N/A
NM_002692.10.4	POLE2	chr14	50130032	50141145	N/A	N/A	2	41.85	N/A
NM_001282129.14.9	SSH2	chr17	27975161	27999147	N/A	N/A	2	41.85	N/A
NM_005121.16.13	MED13	chr17	60059558	60062451	N/A	N/A	2	41.85	N/A
NM_001031835.27.16	PHKB	chr16	47674945	47703328	N/A	N/A	2	41.85	N/A
NM_002750.4.1	MAPK8	chr10	49609654	49618211	N/A	N/A	2	41.85	N/A
NM_032482.7.4	DOT1L	chr19	2189730	2194576	N/A	N/A	2	41.85	N/A
NM_001105515.8.5	ABCC4	chr13	95858785	95863035	N/A	N/A	2	41.85	N/A
NM_001287811.10.9	DNAJC16	chr1	15890423	15890854	N/A	N/A	2	41.85	N/A
NM_018179.2.2	ATF7IP	chr12	14576842	14578407	N/A	N/A	2	41.85	N/A
NM_018056.5.4	TMEM39B	chr1	32542363	32542919	N/A	N/A	2	41.85	N/A
NM_138364.10.4	PRMT9	chr4	148563937	148594277	N/A	N/A	2	41.85	N/A
NM_001164163.14.10	PPP6R3	chr11	68334481	68343511	N/A	N/A	2	41.85	N/A
NM_001184801.4.3	UBR2	chr6	42559888	42562042	N/A	N/A	2	41.85	N/A
NM_018312.10.7	PPP6R3	chr11	68318588	68331900	N/A	N/A	2	41.85	N/A
NM_031417.4.3	MARK4	chr19	45766370	45766625	N/A	N/A	2	41.85	N/A
NM_001134649.7.3	EIF4E3	chr3	71739160	71759635	N/A	N/A	2	41.85	N/A
NM_015155.5.4	LARP4B	chr10	909682	910210	N/A	N/A	2	41.85	N/A
NM_001134937.15.10	FIP1L1	chr4	54280781	54310270	N/A	N/A	2	41.85	N/A
NM_014671.17.15	UBE3C	chr7	157013382	157018233	N/A	N/A	2	41.85	N/A
NM_003972.26.23	BTAF1	chr10	93754291	93768032	N/A	N/A	2	41.85	N/A
NM_005225.3.2	E2F1	chr20	32267560	32268222	N/A	N/A	2	41.85	N/A
NM_014798.4.3	PLEKHM1	chr17_ctg5_hap1	167528	170575	N/A	N/A	2	41.85	N/A
NM_004514.3.2	FOXK2	chr17	80521229	80526077	N/A	N/A	2	41.85	N/A
NM_001287821.18.15	ZWILCH	chr15	66828270	66839043	N/A	N/A	2	41.85	N/A
NM_017905.10.8	TMCO3	chr13	114174930	114193822	N/A	N/A	2	41.85	N/A
NM_022166.3.3	XYLT1	chr16	17352844	17353355	N/A	N/A	2	41.85	N/A
NM_018683.5.3	RNF114	chr20	48561918	48565892	N/A	N/A	2	41.85	N/A
NM_001135147.6.3	SLC39A8	chr4	103225473	103236987	N/A	N/A	2	41.85	N/A
NM_005121.5.4	MED13	chr17	60111147	60112969	N/A	N/A	2	41.85	N/A
NM_001135608.19.15	ARHGAP26	chr5	142434003	142513670	N/A	N/A	2	41.85	N/A
NM_014313.6.2	TMEM50A	chr1	25666964	25683344	N/A	N/A	2	41.85	N/A

NR_045796.21.19	-	chr20	47708582	47711500	N/A	N/A	2	41.85	N/A
NM_003185.14.2	TAF4	chr20	60572605	60589763	N/A	N/A	2	41.85	N/A
NM_000031.2.2	ALAD	chr9	116155726	116155914	N/A	N/A	2	41.85	N/A
NM_001170766.7.3	LCOR	chr10	98667021	98711953	N/A	N/A	2	41.85	N/A
NM_001145811.8.5	SOX6	chr11	16119154	16208501	N/A	N/A	2	41.85	N/A
NM_004788.10.9	UBE4A	chr11	118245610	118247409	N/A	N/A	2	41.85	N/A
NM_001109662.55.42	HECTD4	chr12	112638441	112666615	N/A	N/A	2	41.85	N/A
NM_003384.12.2	VRK1	chr14	97299803	97342457	N/A	N/A	2	41.85	N/A
NM_021729.9.8	VPS11	chr11	118947606	118948357	N/A	N/A	2	41.85	N/A
NM_001031711.9.8	ERGIC1	chr5	172359438	172362313	N/A	N/A	2	41.85	N/A
NM_001042544.25.23	LTBP4	chr19	41122794	41125398	N/A	N/A	2	41.85	N/A
NM_001242906.3.2	ASCC2	chr22	30221075	30228331	N/A	N/A	2	41.85	N/A
NM_002267.9.7	KPNA3	chr13	50296090	50299637	N/A	N/A	2	41.85	N/A
NM_001626.4.2	AKT2	chr19	40761064	40771258	N/A	N/A	2	41.85	N/A
NM_015950.6.3	MRPL2	chr6	43023282	43024183	N/A	N/A	2	41.85	N/A
NM_001136191.6.5	KANK2	chr19	11289020	11289396	N/A	N/A	2	41.85	N/A
NM_018593.3.2	SLC16A10	chr6	111493897	111498868	N/A	N/A	2	41.85	N/A
NM_007368.22.21	RASA3	chr13	114757960	114762215	N/A	N/A	2	41.85	N/A
NM_012138.4.3	AATF	chr17	35310185	35311207	N/A	N/A	2	41.85	N/A
NM_001193268.21.20	EML2	chr19	46116798	46117958	N/A	N/A	2	41.85	N/A
NM_001067.33.30	TOP2A	chr17	38547757	38548989	N/A	N/A	2	41.85	N/A
NM_015030.17.11	FRYL	chr4	48592675	48607850	N/A	N/A	2	41.85	N/A
NM_016525.4.4	UBAP1	chr9	34241182	34242106	N/A	N/A	2	41.85	N/A
NM_005151.13.6	USP14	chr18	192841	204692	N/A	N/A	2	41.85	N/A
NM_014826.33.31	CDC42BPA	chr1	227192694	227204751	N/A	N/A	2	41.85	N/A
NM_007345.3.2	ZNF236	chr18	74561481	74563895	N/A	N/A	2	41.85	N/A
NM_001261392.12.6	NOL10	chr2	10784445	10808849	N/A	N/A	2	41.85	N/A
NM_001114394.12.5	PAPD4	chr5	78936673	78964851	N/A	N/A	2	41.85	N/A
NM_001080491.13.10	USP6NL	chr10	11523768	11527910	N/A	N/A	2	41.85	N/A
NM_014053.3.2	FLVCR1	chr1	213037066	213046160	N/A	N/A	2	41.85	N/A
NM_020244.4.2	CHPT1	chr12	102107867	102110590	N/A	N/A	2	41.85	N/A
NM_025126.3.2	RNF34	chr12	121853961	121855714	N/A	N/A	2	41.85	N/A
NM_001198810.11.9	SLC43A1	chr11	57258696	57259335	N/A	N/A	2	41.85	N/A

NM_003126.3.2	SPTA1	chr1	158653160	158655137	N/A	N/A	2	41.85	N/A
NM_001127890.4.2	SPG21	chr15	65266939	65275931	N/A	N/A	2	41.85	N/A
NM_014845.10.2	FIG4	chr6	110036280	110064975	N/A	N/A	2	41.85	N/A
NM_003126.51.45	SPTA1	chr1	158582606	158589124	N/A	N/A	2	41.85	N/A
NM_001876.8.6	CPT1A	chr11	68562271	68566823	N/A	N/A	2	41.85	N/A
NM_017926.9.3	GPATCH2L	chr14	76633005	76662315	N/A	N/A	2	41.85	N/A
NM_001048183.8.7	PHACTR4	chr1	28800065	28802803	N/A	N/A	2	41.85	N/A
NM_032582.9.6	USP32	chr17	58342772	58348842	N/A	N/A	2	41.85	N/A
NM_001252049.6.2	PCMT1	chr6	150092297	150117636	N/A	N/A	2	41.85	N/A
NM_015473.11.8	HEATR5A	chr14	31849672	31856581	N/A	N/A	2	41.85	N/A
NM_021174.7.6	CCAR2	chr8	22464708	22465578	N/A	N/A	2	41.85	N/A
NM_001162384.11.8	ARHGEF2	chr1	155931451	155932974	N/A	N/A	2	41.85	N/A
NM_012406.11.6	PRDM4	chr12	108133159	108140201	N/A	N/A	2	41.85	N/A
NM_001079559.4.2	HNRNPUL2	chr11	62491058	62491898	N/A	N/A	2	41.85	N/A
NM_001286635.4.2	C6orf89	chr6	36862279	36870210	N/A	N/A	2	41.85	N/A
NM_024755.19.18	SLTM	chr15	59179173	59179739	N/A	N/A	2	41.85	N/A
NM_001282680.15.11	GAPVD1	chr9	128086076	128094908	N/A	N/A	2	41.85	N/A
NR_024054.3.2	-	chr5	69515744	69517842	N/A	N/A	2	41.85	N/A
NM_015197.4.2	PACS2	chr14	105814829	105821514	N/A	N/A	2	41.85	N/A
NM_015017.18.12	USP33	chr1	78183551	78191447	N/A	N/A	2	41.85	N/A
NM_145062.9.4	ZUFSP	chr6	116966876	116980087	N/A	N/A	2	41.85	N/A
NR_036680.9.3	-	chr7	32672154	32718742	N/A	N/A	2	41.85	N/A
NM_174905.6.4	FAM98C	chr19	38895547	38896275	N/A	N/A	2	41.85	N/A
NM_139067.25.23	SMARCC2	chr12	56563312	56563992	N/A	N/A	2	41.85	N/A
NM_014868.10.8	RNF10	chr12	121000747	121001746	N/A	N/A	2	41.85	N/A
NM_001080824.2.2	TRABD2A	chr2	85097348	85097909	N/A	N/A	2	41.85	N/A
NM_000333.5.2	ATXN7	chr3	63884074	63938159	N/A	N/A	2	41.85	N/A
NM_032025.10.10	EIF2A	chr3	150289744	150290316	N/A	N/A	2	41.85	N/A
NM_153211.6.2	TTC39C	chr18	21644103	21663045	N/A	N/A	2	41.85	N/A
NM_006540.10.9	NCOA2	chr8	71071739	71075089	N/A	N/A	2	41.85	N/A
NM_153211.6.5	TTC39C	chr18	21660548	21663045	N/A	N/A	2	41.85	N/A
NR_028473.4.4	-	chr11	61133516	61133688	N/A	N/A	2	41.85	N/A
NM_024773.4.3	KDM8	chr16	27224905	27226329	N/A	N/A	2	41.85	N/A

NM_152699.2.2	SENP5	chr3	196612021	196613565	N/A	N/A	2	41.85	N/A
NM_001256469.3.3	KAT6B	chr10	76602357	76603236	N/A	N/A	2	41.85	N/A
NR_037774.10.8	-	chr10	17746429	17747740	N/A	N/A	2	41.85	N/A
NM_001270764.6.2	CHST15	chr10	125780771	125806240	N/A	N/A	2	41.85	N/A
NM_002569.8.2	FURIN	chr15	91418811	91421534	N/A	N/A	2	41.85	N/A
NM_152896.8.6	UHRF2	chr9	6477621	6482099	N/A	N/A	2	41.85	N/A
NM_001193431.14.8	PTPN22	chr1	114377531	114397671	N/A	N/A	2	41.85	N/A
NM_001145819.8.7	SOX6	chr11	16119154	16133469	N/A	N/A	2	41.85	N/A
NM_001206947.12.2	PICALM	chr11	85707868	85742653	N/A	N/A	2	41.85	N/A
NM_198859.7.3	PRICKLE2	chr3	64132505	64148805	N/A	N/A	2	41.85	N/A
NM_145061.7.3	SKA3	chr13	21732060	21746643	N/A	N/A	2	41.85	N/A
NM_015601.15.9	HERC4	chr10	69748419	69773943	N/A	N/A	2	41.85	N/A
NM_152468.9.6	TMC8	chr17	76129486	76131090	N/A	N/A	2	41.85	N/A
NM_058246.8.6	DNAJB6	chr7	157174939	157178305	N/A	N/A	2	41.85	N/A
NM_003268.5.4	TLR5	chr1	223305816	223308206	N/A	N/A	2	41.85	N/A
NM_016940.4.2	RWDD2B	chr21	30380081	30380942	N/A	N/A	2	41.85	N/A
NM_001281749.15.12	SIK3	chr11	116734383	116744352	N/A	N/A	2	41.85	N/A
NM_001812.7.6	CENPC	chr4	68383873	68385220	N/A	N/A	2	41.85	N/A
NM_001128848.32.29	SMARCA4	chr19	11168933	11170863	N/A	N/A	2	41.85	N/A
NM_023034.17.15	WHSC1L1	chr8	38147992	38157108	N/A	N/A	2	41.85	N/A
NM_014708.36.34	KNTC1	chr12	123067251	123069537	N/A	N/A	2	41.85	N/A
NM_014801.19.13	PCNX2	chr1	233296028	233344435	N/A	N/A	2	41.85	N/A
NM_153188.6.5	TNPO1	chr5	72157634	72161556	N/A	N/A	2	41.85	N/A
NM_017673.16.14	SWT1	chr1	185183638	185200840	N/A	N/A	2	41.85	N/A
NM_004843.6.5	IL27RA	chr19	14153264	14153601	N/A	N/A	2	41.85	N/A
NM_152244.5.3	SNX11	chr17	46189392	46190763	N/A	N/A	2	41.85	N/A
NM_015866.5.3	PRDM2	chr1	14057494	14068652	N/A	N/A	2	41.85	N/A
NM_012411.16.13	PTPN22	chr1	114372213	114377061	N/A	N/A	2	41.85	N/A
NM_001286717.16.13	AGTPBP1	chr9	88233897	88248289	N/A	N/A	2	41.85	N/A
NM_013373.10.2	ZDHHC8	chr22	20126716	20131279	N/A	N/A	2	41.85	N/A
NM_000637.6.5	GSR	chr8	30557597	30560757	N/A	N/A	2	41.85	N/A
NM_144649.6.3	TMEM71	chr8	133740043	133769540	N/A	N/A	2	41.85	N/A
NM_030767.11.7	AKNA	chr9	117121873	117126899	N/A	N/A	2	41.85	N/A

NM_014063.4.2	DBNL	chr7	44089823	44092540	N/A	N/A	2	41.85	N/A
NR_110254.11.5	-	chr4	83857133	83867627	N/A	N/A	2	41.85	N/A
NM_001271052.14.13	ADGRE2	chr19	14862247	14863338	N/A	N/A	2	41.85	N/A
NM_001201407.5.4	ZNF778	chr16	89289564	89291210	N/A	N/A	2	41.85	N/A
NM_020784.9.2	TXNDC16	chr14	52977957	53011089	N/A	N/A	2	41.85	N/A
NM_003400.22.17	XPO1	chr2	61710091	61717911	N/A	N/A	2	41.85	N/A
NR_024386.4.3	-	chr17	62817884	62818511	N/A	N/A	2	41.85	N/A
NM_001316.8.7	CSE1L	chr20	47685251	47686834	N/A	N/A	2	41.85	N/A
NM_001128840.3.2	CACNA1D	chr3	53531178	53535747	N/A	N/A	2	41.85	N/A
NM_153645.6.6	NUP50	chr22	45574118	45574781	N/A	N/A	2	41.85	N/A
NM_013411.6.5	AK2	chr1	33478807	33480195	N/A	N/A	2	41.85	N/A
NM_001271010.6.2	FLI1	chr11	128628009	128651918	N/A	N/A	2	41.85	N/A
NR_037619.4.2	-	chr16	66642211	66643906	N/A	N/A	2	41.85	N/A
NM_005121.15.13	MED13	chr17	60061531	60062451	N/A	N/A	2	41.85	N/A
NM_206943.9.5	LTBP1	chr2	33359859	33447218	N/A	N/A	2	41.85	N/A
NM_014708.12.9	KNTC1	chr12	123030722	123032516	N/A	N/A	2	41.85	N/A
NM_014109.21.20	ATAD2	chr8	124349864	124351686	N/A	N/A	2	41.85	N/A
NM_001098823.2.2	TMEM91	chr19	41884185	41884424	N/A	N/A	2	41.85	N/A
NM_152879.3.2	DGKD	chr2	234296902	234299129	N/A	N/A	3	62.775	N/A
NM_015226.21.11	CLEC16A	chr16	11114049	11220003	N/A	N/A	3	62.775	N/A
NM_007124.32.28	UTRN	chr6	144808683	144814592	N/A	N/A	3	62.775	N/A
NM_001013836.3.2	MAD1L1	chr7	2269618	2270362	N/A	N/A	3	62.775	N/A
NM_003932.5.3	ST13	chr22	41236629	41244373	N/A	N/A	3	62.775	N/A
NM_003550.3.2	MAD1L1	chr7	2269618	2270399	N/A	N/A	3	62.775	N/A
NR_003655.3.2	-	chr7	44054204	44056122	N/A	N/A	3	62.775	N/A
NM_005493.12.6	RANBP9	chr6	13632601	13644961	N/A	N/A	3	62.775	N/A
NM_198531.8.2	ATP9B	chr18	76856475	76936907	N/A	N/A	3	62.775	N/A
NM_139241.10.5	FGD4	chr12	32751430	32764217	N/A	N/A	3	62.775	N/A
NM_001199381.3.2	RNF145	chr5	158621723	158630664	N/A	N/A	3	62.775	N/A
NM_001121.9.4	ADD3	chr10	111876016	111882050	N/A	N/A	3	62.775	N/A
NM_002372.8.5	MAN2A1	chr5	109091029	109110666	N/A	N/A	3	62.775	N/A
NM_001247996.14.4	ASAP1	chr8	131164981	131370389	N/A	N/A	3	62.775	N/A
NM_003120.4.3	SPI1	chr11	47380394	47381591	N/A	N/A	3	62.775	N/A

NM_002372.4.2	MAN2A1	chr5	109049220	109065214	N/A	N/A	3	62.775	N/A
NM_002887.5.2	RARS	chr5	167915606	167921655	N/A	N/A	3	62.775	N/A
NM_020119.3.2	ZC3HAV1	chr7	138768525	138774505	N/A	N/A	3	62.775	N/A
NM_002558.7.2	P2RX1	chr17	3806495	3808661	N/A	N/A	3	62.775	N/A
NM_020716.5.4	GRAMD1B	chr11	123464789	123465546	N/A	N/A	3	62.775	N/A
NM_153369.3.2	MFSD4B	chr6	111583459	111585149	N/A	N/A	3	62.775	N/A
NM_001270940.9.8	XPO6	chr16	28163979	28167848	N/A	N/A	3	62.775	N/A
NM_006788.6.5	RALBP1	chr18	9524591	9525849	N/A	N/A	3	62.775	N/A
NM_017988.4.2	SCYL2	chr12	100676720	100691953	N/A	N/A	3	62.775	N/A
NM_003370.7.4	VASP	chr19	46024579	46026076	N/A	N/A	3	62.775	N/A
NM_001033555.10.6	SPECC1	chr17	20149238	20163607	N/A	N/A	3	62.775	N/A
NM_152493.8.7	ZNF362	chr1	33760537	33760906	N/A	N/A	3	62.775	N/A
NM_018489.5.4	ASH1L	chr1	155408117	155429689	N/A	N/A	3	62.775	N/A
NM_018590.3.2	CSGALNACT2	chr10	43650344	43654379	N/A	N/A	3	62.775	N/A
NM_014643.3.3	ZNF516	chr18	74153200	74155167	N/A	N/A	3	62.775	N/A
NM_005720.5.4	ARPC1B	chr7	98985661	98987635	N/A	N/A	3	62.775	N/A
NM_015642.9.9	ZBTB20	chr3	114069120	114070725	N/A	N/A	3	62.775	N/A
NM_001244713.4.2	RAD23B	chr9	110062421	110068928	N/A	N/A	3	62.775	N/A
NM_015443.2.2	KANSL1	chr17_ctg5_hap1	616744	618122	N/A	N/A	3	62.775	N/A
NM_017772.11.5	TBC1D22B	chr6	37250657	37284606	N/A	N/A	3	62.775	N/A
NM_018442.4.2	DCAF6	chr1	167921037	167944253	N/A	N/A	3	62.775	N/A
NM_003922.24.22	HERC1	chr15	64004973	64008672	N/A	N/A	3	62.775	N/A
NM_001276471.8.7	CORO1C	chr12	109046047	109048186	N/A	N/A	3	62.775	N/A
NR_027028.4.4	-	chr5	21491429	21491617	N/A	N/A	3	62.775	N/A
NM_017742.5.2	ZCCHC2	chr18	60206913	60217693	N/A	N/A	3	62.775	N/A
NM_032012.14.13	TMEM245	chr9	111812562	111812972	N/A	N/A	3	62.775	N/A
NM_014494.5.3	TNRC6A	chr16	24762046	24788679	N/A	N/A	3	62.775	N/A
NM_015020.13.7	PHLPP2	chr16	71697801	71713438	N/A	N/A	3	62.775	N/A
NM_006699.4.2	MAN1A2	chr1	117944807	117957453	N/A	N/A	3	62.775	N/A
NM_001284238.4.3	GTDC1	chr2	144966169	144969146	N/A	N/A	3	62.775	N/A
NM_171982.3.3	TRIM35	chr8	27151596	27151827	N/A	N/A	3	62.775	N/A
NM_001260.12.10	CDK8	chr13	26974589	26975761	N/A	N/A	3	62.775	N/A
NM_173165.2.2	NFATC3	chr16	68155889	68157024	N/A	N/A	3	62.775	N/A

NM_001286188.7.3	SCAF8	chr6	155095122	155116273	N/A	N/A	3	62.775	N/A
NM_002582.5.4	PARN	chr16	14720962	14721193	N/A	N/A	3	62.775	N/A
NM_003410.5.4	ZFX	chrX	24190831	24197887	N/A	N/A	3	62.775	N/A
NM_007368.4.2	RASA3	chr13	114806475	114839312	N/A	N/A	3	62.775	N/A
NM_021117.11.5	CRY2	chr11	45883610	45893784	N/A	N/A	3	62.775	N/A
NM_032440.7.5	LCOR	chr10	98703869	98711953	N/A	N/A	3	62.775	N/A
NM_001077351.5.2	RBM23	chr14	23375403	23380612	N/A	N/A	3	62.775	N/A
NM_020823.6.3	TMEM181	chr6	159001971	159010814	N/A	N/A	3	62.775	N/A
NM_006327.6.2	TIMM23	chr10	51606987	51620382	N/A	N/A	3	62.775	N/A
NM_017844.4.3	ANKMY1	chr2	241492330	241494472	N/A	N/A	3	62.775	N/A
NM_178566.8.4	ZDHHC21	chr9	14639893	14674383	N/A	N/A	3	62.775	N/A
NM_001206703.7.2	SP100	chr2	231307651	231314970	N/A	N/A	3	62.775	N/A
NM_001142289.10.9	MGRN1	chr16	4721391	4723658	N/A	N/A	3	62.775	N/A
NM_013438.4.2	UBQLN1	chr9	86294689	86301070	N/A	N/A	3	62.775	N/A
NM_018091.14.11	ELP3	chr8	28013458	28019595	N/A	N/A	3	62.775	N/A
NM_182480.4.2	COQ6	chr14	74420137	74422631	N/A	N/A	3	62.775	N/A
NM_001243439.4.4	SPECC1	chr17	20107645	20109225	N/A	N/A	3	62.775	N/A
NM_001286612.7.5	REPS1	chr6	139264649	139265759	N/A	N/A	3	62.775	N/A
NM_001282750.5.2	SUCO	chr1	172520651	172526934	N/A	N/A	3	62.775	N/A
NM_001163735.12.8	MYO19	chr17	34869271	34871833	N/A	N/A	3	62.775	N/A
NR_073135.13.12	-	chr22	26853824	26854543	N/A	N/A	3	62.775	N/A
NM_005177.17.15	ATP6V0A1	chr17	40650941	40653322	N/A	N/A	3	62.775	N/A
NM_005437.8.8	NCOA4	chr10	51584615	51585599	N/A	N/A	3	62.775	N/A
NM_001080547.4.2	SPI1	chr11	47380394	47397283	N/A	N/A	3	62.775	N/A
NM_007318.5.2	PSEN1	chr14	73614502	73640415	N/A	N/A	3	62.775	N/A
NM_001286724.5.2	FAM120A	chr9	96233422	96261168	N/A	N/A	3	62.775	N/A
NM_012215.8.5	MGEA5	chr10	103559998	103567658	N/A	N/A	3	62.775	N/A
NM_001287815.14.13	UBAP2L	chr1	154223516	154224129	N/A	N/A	3	62.775	N/A
NM_003591.18.13	CUL2	chr10	35305119	35320547	N/A	N/A	3	62.775	N/A
NM_020475.29.29	ANK1	chr8	41551415	41551620	N/A	N/A	3	62.775	N/A
NM_001130047.7.2	TBCEL	chr11	120916382	120930794	N/A	N/A	3	62.775	N/A
NM_181050.5.3	AXIN1	chr16	354303	364683	N/A	N/A	3	62.775	N/A
NM_198889.29.28	ANKRD17	chr4	73950965	73958017	N/A	N/A	3	62.775	N/A

NM_018482.20.17	ASAP1	chr8	131130406	131138335	N/A	N/A	3	62.775	N/A
NM_006909.17.16	RASGRF2	chr5	80419460	80422982	N/A	N/A	3	62.775	N/A
NM_018638.6.3	ETNK1	chr12	22811947	22826594	N/A	N/A	4	83.7	N/A
NM_004544.9.7	NDUFA10	chr2	240929490	240946787	N/A	N/A	4	83.7	N/A
NM_015308.4.3	FNBP4	chr11	47774467	47776216	N/A	N/A	4	83.7	N/A
NR_038272.7.3	-	chr2	179400458	179407088	N/A	N/A	4	83.7	N/A
NM_000052.13.10	ATP7A	chrX	77268375	77275895	N/A	N/A	4	83.7	N/A
NM_015093.5.5	TAB2	chr6	149699153	149700654	N/A	N/A	4	83.7	N/A
NM_001164273.9.6	MGA	chr15	41999925	42005694	N/A	N/A	4	83.7	N/A
NM_053043.5.2	RBM33	chr7	155457868	155473602	N/A	N/A	4	83.7	N/A
NM_001130913.3.2	ZNF720	chr16	31733946	31734674	N/A	N/A	4	83.7	N/A
NM_078483.10.2	SLC36A1	chr5	150838348	150859050	N/A	N/A	4	83.7	N/A
NM_001243403.18.11	CLEC16A	chr16	11114049	11154879	N/A	N/A	4	83.7	N/A
NM_033111.6.3	N4BP2L2	chr13	33091993	33101669	N/A	N/A	4	83.7	N/A
NM_001112801.1.1	SLC8A1	chr2	40655612	40657444	N/A	N/A	4	83.7	N/A
NM_006699.5.2	MAN1A2	chr1	117944807	117963271	N/A	N/A	4	83.7	N/A
NM_032433.7.6	ZNF333	chr19	14815865	14817585	N/A	N/A	4	83.7	N/A
NR_003187.7.6	-	chr7	74578130	74578824	N/A	N/A	4	83.7	N/A
NM_001142464.4.3	ECSIT	chr19	11623870	11625036	N/A	N/A	4	83.7	N/A
NM_012421.5.2	RLF	chr1	40654726	40668286	N/A	N/A	4	83.7	N/A
NM_018062.7.2	FANCL	chr2	58425728	58459247	N/A	N/A	4	83.7	N/A
NM_138782.21.20	FCHO2	chr5	72370568	72373320	N/A	N/A	4	83.7	N/A
NM_005493.9.6	RANBP9	chr6	13639794	13644961	N/A	N/A	4	83.7	N/A
NM_015071.17.13	ARHGAP26	chr5	142416760	142437312	N/A	N/A	4	83.7	N/A
NM_001166006.2.2	EPB41	chr1	29313942	29314417	N/A	N/A	4	83.7	N/A
NM_207292.4.3	MBNL1	chr3	152132729	152150709	N/A	N/A	4	83.7	N/A
NM_015106.8.5	RAD54L2	chr3	51664287	51668051	N/A	N/A	4	83.7	N/A
NM_001080541.2.2	MGA	chr15	41961025	41962156	N/A	N/A	4	83.7	N/A
NM_001282858.21.21	GON4L	chr1	155734790	155736500	N/A	N/A	4	83.7	N/A
NM_000876.18.15	IGF2R	chr6	160467529	160469575	N/A	N/A	4	83.7	N/A
NM_182838.5.2	SLC35E2	chr1	1666128	1671143	N/A	N/A	4	83.7	N/A
NM_001282529.8.6	UBAP2	chr9	33953282	33963789	N/A	N/A	5	104.62	N/A
NM_005128.21.20	DOPEY2	chr21	37619814	37620866	N/A	N/A	5	104.62	N/A

NM_001042572.5.3	CHD2	chr15	93467550	93472321	N/A	N/A	5	104.62	N/A
NM_005990.18.14	STK10	chr5	171479932	171488272	N/A	N/A	5	104.62	N/A
NM_001135022.10.7	ELMOD3	chr2	85595808	85598685	N/A	N/A	5	104.62	N/A
NM_001243248.8.7	NPRL3	chr16	148142	150507	N/A	N/A	5	104.62	N/A
NM_144649.7.3	TMEM71	chr8	133734285	133769540	N/A	N/A	5	104.62	N/A
NM_021231.2.2	CACTIN	chr19	3623685	3624160	N/A	N/A	5	104.62	N/A
NM_012398.5.4	PIP5K1C	chr19	3660963	3661999	N/A	N/A	5	104.62	N/A
NM_018454.10.5	NUSAP1	chr15	41648236	41669502	N/A	N/A	5	104.62	N/A
NM_001146055.4.2	SNCA	chr4	90743396	90756843	N/A	N/A	5	104.62	N/A
NM_017626.5.4	DNAJB12	chr10	74100546	74100928	N/A	N/A	5	104.62	N/A
NM_001001548.14.6	CD36	chr7	80292305	80303463	N/A	N/A	5	104.62	N/A
NM_021937.4.3	EEFSEC	chr3	127980970	127983624	N/A	N/A	6	125.55	N/A
NM_001114403.3.2	UPK3BL	chr7	102280650	102281220	N/A	N/A	6	125.55	N/A
NM_018248.9.8	NEIL3	chr4	178274461	178281831	N/A	N/A	6	125.55	N/A
NM_001289003.8.6	PHLPP2	chr16	71712657	71715808	N/A	N/A	6	125.55	N/A
NM_001278162.2.2	HIPK3	chr11	33307958	33309057	N/A	N/A	6	125.55	N/A
NM_001172221.9.8	SCMH1	chr1	41536266	41541123	N/A	N/A	6	125.55	N/A
NM_032341.6.5	DDI2	chr1	15964801	15970145	N/A	N/A	6	125.55	N/A
NM_001100400.9.5	PDS5A	chr4	39915230	39927553	N/A	N/A	6	125.55	N/A
NM_015358.14.13	MORC3	chr21	37734480	37736557	N/A	N/A	7	146.47	N/A
NM_001177387.3.2	ATXN7	chr3	63898263	63898901	N/A	N/A	7	146.47	N/A
NM_018248.8.8	NEIL3	chr4	178274461	178274882	N/A	N/A	7	146.47	N/A
NM_005177.17.16	ATP6V0A1	chr17	40652724	40653322	N/A	N/A	7	146.47	N/A
NM_017508.12.8	SOX6	chr11	16036487	16119234	N/A	N/A	7	146.47	N/A
NM_003922.27.22	HERC1	chr15	63988322	64008672	N/A	N/A	7	146.47	N/A
NM_173163.3.2	NFATC3	chr16	68155889	68160513	N/A	N/A	7	146.47	N/A
NM_032233.6.2	SETD3	chr14	99924615	99932150	N/A	N/A	8	167.4	N/A
NM_020728.13.9	ESYT2	chr7	158552176	158557544	N/A	N/A	8	167.4	N/A
NM_001286375.12.10	ITGAX	chr16	31373156	31374074	N/A	N/A	8	167.4	N/A
NM_001242925.3.3	TMCC2	chr1	205238077	205239012	N/A	N/A	8	167.4	N/A
NM_001114380.13.12	ITGAL	chr16	30507417	30507887	N/A	N/A	9	188.32	N/A
NM_138782.21.17	FCHO2	chr5	72354259	72373320	N/A	N/A	9	188.32	N/A
NM_001282681.14.13	GAPVD1	chr9	128099296	128099870	N/A	N/A	9	188.32	N/A

NM_014787.4.2	DNAJC6	chr1	65830317	65831879	N/A	N/A	10	209.25	N/A
NM_004448.12.5	ERBB2	chr17	37866065	37872192	N/A	N/A	10	209.25	N/A
NM_001206947.12.9	PICALM	chr11	85707868	85714494	N/A	N/A	10	209.25	N/A
NM_001167608.8.4	RHBDD1	chr2	227729319	227779067	N/A	N/A	12	251.1	N/A
**NM_015024.9.6**	**XPO7**	**chr8**	**21832180**	**21837714**	**N/A**	**N/A**	**13**	**272.02**	**N/A**
NM_001199691.6.2	*TMEM56-RWDD3*	*chr1*	*95609446*	*95639445*	*N/A*	*N/A*	*13*	*272.02*	*N/A*
NR_027774.3.3	*-*	*chr17_ctg5_hap1*	*167528*	*168155*	*N/A*	*N/A*	*14*	*292.95*	*N/A*
**NM_001166693.4.3**	**AFF1**	**chr4**	**87967317**	**87968746**	**N/A**	**N/A**	**15**	**313.87**	**N/A**
**NM_001143971.2.2**	**CDYL**	**chr6**	**4891946**	**4892613**	**N/A**	**N/A**	**15**	**313.87**	**N/A**
**NM_022763.6.5**	**FNDC3B**	**chr3**	**171965322**	**171969331**	**N/A**	**N/A**	**16**	**334.8**	**N/A**
**NM_201559.3.3**	**FOXO3**	**chr6**	**108984657**	**108986092**	**N/A**	**N/A**	**17**	**355.72**	**N/A**
