# Supplementary figures and images for: circRNAs expressed in human peripheral blood are associated with human aging phenotypes, cellular senescence and mouse lifespan
Source: GeroScience. 2019 Dec 6;42(1):183–99. doi: 10.1007/s11357-019-00120-z (PMC7031184; doi:10.1007/s11357-019-00120-z)

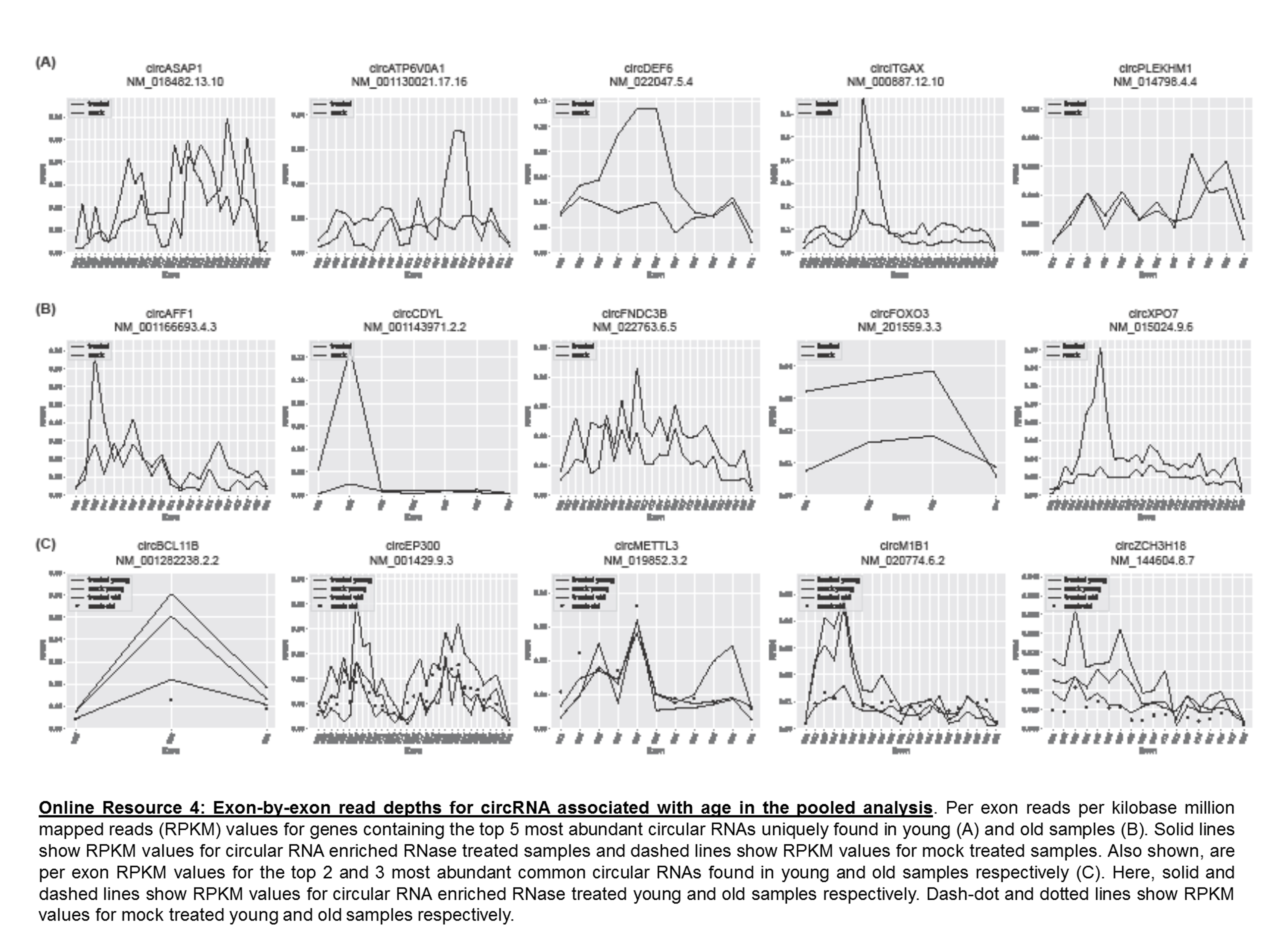

Supplement: Supplementary file 4 — (PNG 6293 kb) [file 11357_2019_120_Fig4_ESM.png]

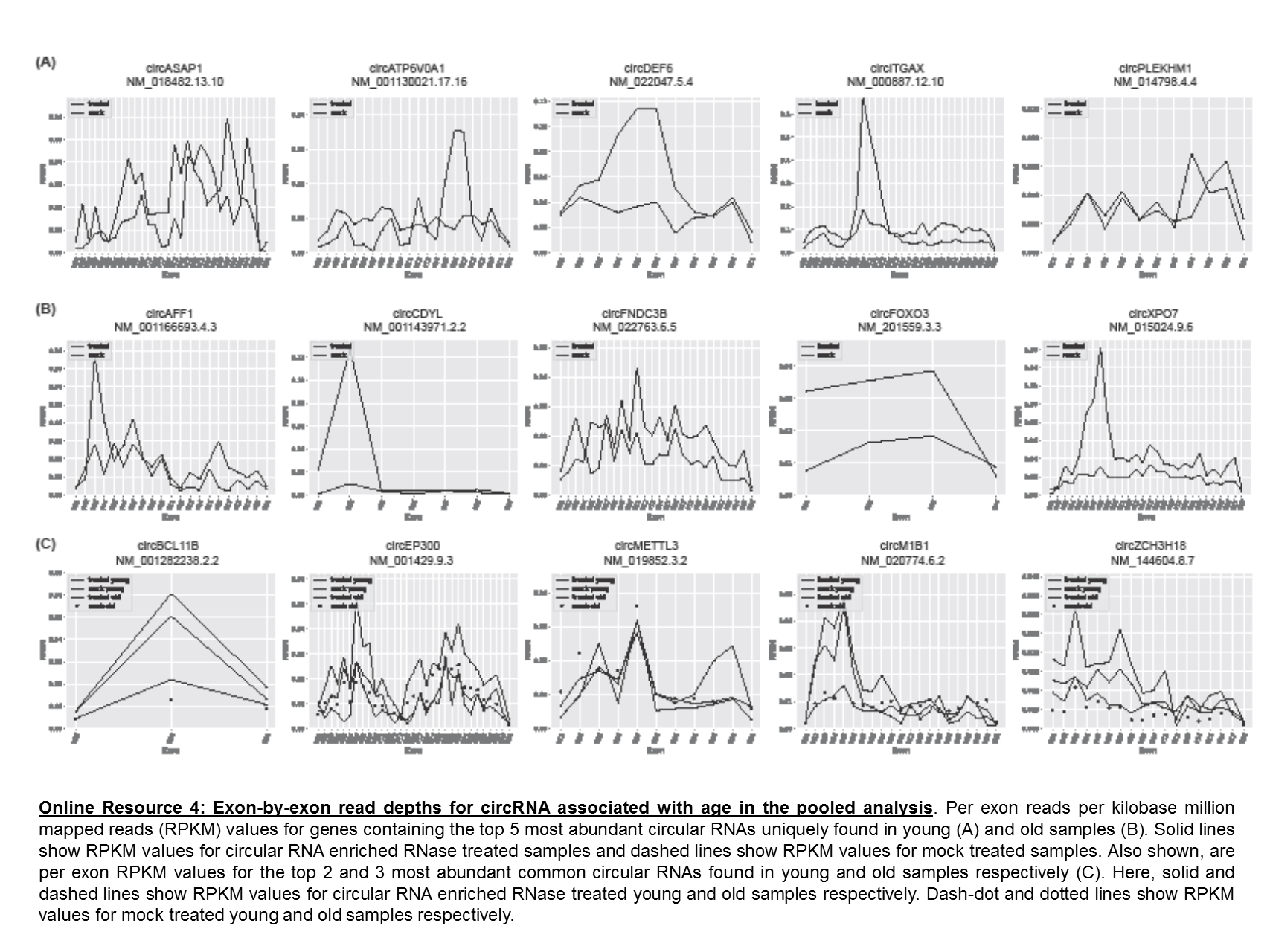

Supplement: Supplementary file 5 — High Resolution Image (TIF 1267 kb) [file 11357_2019_120_MOESM4_ESM.tif]
